# ﻿Fungal frontiers in toxic terrain: Revealing culturable fungal communities in Serpentine paddy fields of Taiwan

**DOI:** 10.3897/imafungus.16.155308

**Published:** 2025-06-27

**Authors:** Kai-Wen Cheng, Jiue-in Yang, Piroonporn Srimongkol, Marc Stadler, Aphichart Karnchanatat, Hiran A. Ariyawansa

**Affiliations:** 1 Department of Plant Pathology and Microbiology, National Taiwan University, Taipei City, Taiwan; 2 Department of Nematology, University of California, Riverside, CA 92521, USA; 3 The Institute of Biotechnology and Genetic Engineering, Chulalongkorn University, Bangkok, Thailand; 4 High-Value Food from Mushrooms and Bioactive Plants in the Green Economy Value Chain Research Group, The Institute of Biotechnology and Genetic Engineering, Chulalongkorn University, 254 Phayathai Road, Pathumwan, Bangkok, 10330, Thailand; 5 Department Microbial Drugs, Helmholtz Centre for Infection Research GmbH (HZI), Braunschweig, Germany; 6 Center of Excellence in Bioconversion and Bioseparation for Platform Chemical Production, The Institute of Biotechnology and Genetic Engineering, Chulalongkorn University, 254 Phayathai Road, Pathumwan, Bangkok, 10330, Thailand

**Keywords:** 11-new species, heavy metal, phylogeny, serpentine soil, taxonomy

## Abstract

Serpentine soils are predominantly distributed along the Circum-Pacific margin and the Mediterranean, including eastern Taiwan. These soils are characterized by high levels of heavy metals, including nickel and chromium, and a low calcium-to-magnesium ratio, creating a unique environment that fosters microorganisms with specialized traits. In this study, culture-dependent isolation methods were used to elucidate the composition of culturable fungal communities in serpentine-characterized paddy fields in eastern Taiwan. A total of 154 fungal isolates were isolated from serpentine paddy fields in eastern Taiwan. These isolates were grouped into 79 strains based on colony morphology and were subsequently evaluated through morphological and multi-locus phylogenetic analyses. The results revealed that 60% of the strains belong to the class *Dothideomycetes*, followed by 21% in *Sordariomycetes* and 19% in *Eurotiomycetes*. At the genus level, *Westerdykella* was the dominant genus, presenting 35% of the total of isolated strains, followed by *Pyrenochaetopsis* (20%), *Talaromyces* (19%), and *Pseudorhypophila* (8%). The study reports 11 novel species: *Cylindrotrichumformosanum***sp. nov.**, *Dimorphisetaformosana***sp. nov.**, *D.serpentinicola***sp. nov.**, *Parasarocladiumformosum***sp. nov.**, *Phialoparvumformosanum***sp. nov.**, *Poaceascomaserpentinum***sp. nov.**, *Pseudorhypophilaformosana***sp. nov.**, *Sarocladiumformosanum***sp. nov.**, *S.serpentinicola***sp. nov.**, *Talaromycestaiwanensis***sp. nov.**, and *Westerdykellaformosana***sp. nov.** Additionally, 11 known species are reported for the first time in Taiwan: *Pseudothielaviaterricola*, *Pseudoxylomycesaquaticus*, *Pyrenochaetopsisoryzicola*, *Py.paucisetosa*, *Setophaeosphaeriamicrospora*, *Talaromycesadpressus*, *T.thailandensis*, *Westerdykellaaquatica*, *W.capitulum*, *W.dispersa*, and *W.globosa*. In addition, this study presents the first documented asexual morph within the genus *Poaceascoma*, represented by *P.serpentinum*. These discoveries will be valuable for future evaluations of the potential uses and functions of these species as bioremediation agents.

## ﻿Introduction

Serpentinite is a metamorphic rock produced by the hydrothermal transformation of ultramafic rocks and contains serpentine minerals (Alexander 2004). Serpentine soils, despite covering only about 1% exposed surface of the Earth, hold significant environmental and ecological importance for several reasons (Oze et al. 2008; Kumarathilaka et al. 2014). They are widespread across regions of the Circum-Pacific margin and the Mediterranean Sea (Kumarathilaka et al. 2014). Simultaneously, these environments exhibit low levels of essential plant nutrients, including calcium, potassium, nitrogen, and phosphorus (Cheng et al. 2009). Furthermore, during serpentinization (i.e., the hydration of originally anhydrous ultramafic rocks), calcium content decreases, which leads to a low calcium-to-magnesium ratio (McGahan et al. 2008; Cheng et al. 2009). Collectively, these conditions impose significant stress on most plant life. Serpentines are generally unstable types of soil (Bonifacio et al. 1997) and have the potential to release significant amounts of heavy metals, particularly chromium and nickel, into the environment during the weathering process (Hseu et al. 2015).

The toxicity caused by high concentrations of nickel and chromium in serpentinite soils, referred to as the “serpentine syndrome,” results in poor plant productivity and endemism (Fernández et al. 1999; Oze et al. 2008). Serpentine sites are ecologically significant due to their exceptionally high proportion of endemic species uniquely adapted to these extreme conditions (Daghino et al. 2012). A recent study conducted in Italy demonstrated that the fungal genera *Aspergillus*, *Penicillium*, and *Cladosporium* are dominant in serpentine soils (Daghino et al. 2012). Notably, fungal groups such as *Aspergillus* and *Penicillium*, classified within *Eurotiomycetes*, are frequently reported from environments with high levels of heavy metals (Gadd 2007; Roccotiello et al. 2010). Microbial communities vary according to precipitation, soil texture, and weathering processes. For instance, it has been reported that fungal community structure differs between high and low precipitation conditions, with fungal diversity being lowest in drier environments. (Solano-Arguedas et al. 2022; Botha et al. 2024). Additionally, microbes such as fungi in serpentine environments generally exhibit stronger resistance to heavy metals, showing their adaptability to extreme conditions (Gonçalves and Martins-Loução 2009). Therefore, researchers have shown great interest in exploring the acclimation and survival strategies of microorganisms in serpentine environments, aiming to use these microbes as bioremediators to reduce heavy metal concentrations in agricultural fields (Hou et al. 2020).

In Taiwan, serpentinites and serpentine soils are mainly found in Yilan County (Nan’ao), Hualien County (Fonglin, Ruisui, Shoufeng, Wanrung, Yuli), and Taitung County (Beinan, Chishang, Donghe, Guanshan) (Liu et al. 2007; Cheng et al. 2009; Hsieh 2020). Their elevated chromium and nickel content may pose potential risks to local crops, the environment, and groundwater (Hseu and Lai 2017). The mean total Ni concentration in the serpentine fields was 472 mg/kg, significantly exceeding the natural background levels and soil control standards in Taiwan (Hseu and Lai 2017). Recent studies have repeatedly reported the remarkable diversity of the mycobiota in Taiwan including the discovery of novel species (Ariyawansa et al. 2020; Chuang et al. 2024; Hsu et al. 2024). Although several studies have been carried out on the bacterial community’s structure in these soil types in Taiwan (Koner et al. 2023; Kumar et al. 2023), no comprehensive studies have focused on the culturable fungal communities in serpentine environments. Hence, the present work aimed at the characterization of the culturable fungal communities in serpentine-characterized paddy fields in eastern Taiwan using a polyphasic taxonomic approach.

## ﻿Materials and methods

### ﻿Sample collection and fungal isolation

**Figure 1.**
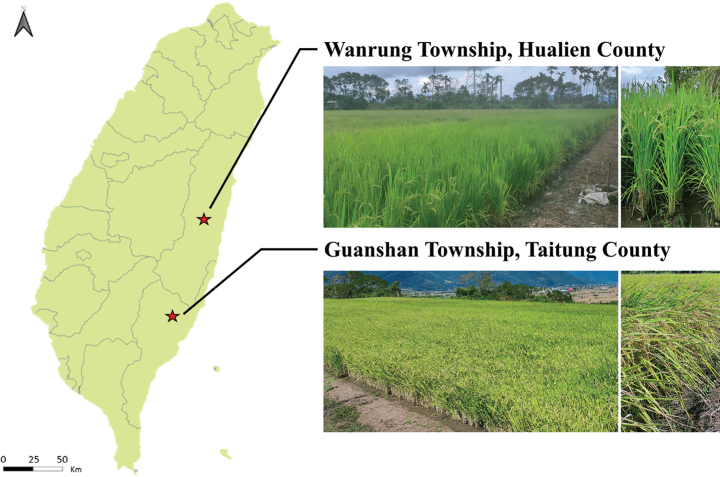
Sampling site location.

A total of six serpentine soil sampling sites from a previous study (Hsieh 2020) were surveyed from rice fields in eastern Taiwan during November 2022 (Table 1 and Fig. 1). According to Hsieh (2020), the concentrations of nickel and chromium in serpentine soils in the surveyed area can reach up to 675 mg/kg and 864 mg/kg, respectively, compared to 57 mg/kg and 150 mg/kg in non-serpentine soils. From each site, 50 soil samples were randomly collected at depths of 5 to 15 cm. For each sample, 10 g of soil was mixed with 50 ml of sterilized water in a 50 ml centrifuge tube (LabServ^®^). The tubes were thoroughly vortexed and subjected to serial dilution. Finally, 10^-4^ dilution solutions were plated onto three different culture media in 9-cm Petri dishes. To obtain the various fungi with different nutrient requirements, three types of culture media were used: half-strength potato dextrose agar (½ PDA), half-strength malt extract agar (½ MEA), and 1.5% water agar (1.5% WA). In total, five plates were used for each type of medium (Khan et al. 2019; Latz et al. 2020). The media were prepared at half-strength concentrations to prevent the overly rapid growth of certain fungi (Su et al. 2012). Additionally, 100 mg/L ampicillin was added to inhibit the growth of non-fungal organisms (Ellegaard-Jensen et al. 2013). Fungal mycelial growth was observed at 36 hours, and single hyphal tip isolation was performed twice using a 27G×½” needle (Terumo AGANI^TM^). The isolated single colonies were then maintained on PDA at room temperature (25 °C ± 1 °C) for further studies.

**Table 1.** Details of the Sampling locations.

Sample name	Location	GPS coordination
SR1	Wanrung Township, Hualien County	23°42'40.3"N, 121°24'48.2"E
SR2	Wanrung Township, Hualien County	23°42'41.2"N, 121°24'41.8"E
SR3	Guanshan Township, Taitung County	23°02'18.2"N, 121°11'25.0"E
SR4	Guanshan Township, Taitung County	23°02'17.6"N, 121°11'26.3"E
SR5	Guanshan Township, Taitung County	23°02'14.8"N, 121°11'22.6"E
SR6	Guanshan Township, Taitung County	23°02'12.8"N, 121°11'22.0"E

### ﻿Morphological examination of fungal colonies and reproductive structures

Morphological characterization was conducted on PDA and included assessments of colony size, colony color, hyphal growth direction, and pigment diffusion observed in the culture medium. Isolates displaying similar morphological traits, grown on the same medium and collected from the same field, were considered identical; only one representative per group was selected for molecular identification (Watanabe 2002). Colonial characteristics of the fungal strains were recorded after incubation on PDA at 25 °C for 14 days. For strains identified as *Talaromyces*, five additional media, namely, Czapek yeast autolysate agar (CYA), CYA supplemented with 5% NaCl (CYAS), malt extract agar (MEA), oatmeal agar (OA), and yeast extract sucrose agar (YESA) were also used to observe colony characteristics, which were likewise recorded after 7 and 14 days of incubation at 25 °C. In addition, CYA and MEA were used to record the colony size after 7 days of incubation at 25 °C, 30 °C, and 37 °C (Yilmaz et al. 2014). PDA and 1.5% WA supplemented with sterile carnation leaves (*Dianthuscaryophyllus*) were used to induce sporulation of selected fungal strains (Fisher et al. 1982). Microscopic characteristics were observed under a dissecting microscope (Leica SAPO, Germany) and a compound microscope (Olympus BX51, Japan). Photographs were captured using a Leica MC190 HD camera (Leica Microsystems, Wetzlar, Germany) equipped with a microscope. Sexual and asexual stages of fungal isolates observed in culture were imaged using differential interference contrast and measured using cellSense Standard software (Olympus). Voucher specimens were deposited in the herbarium of the Department of Plant Pathology and Microbiology, National Taiwan University Herbarium (NTUPPMH. Living cultures are stored at the Department of Plant Pathology and Microbiology, National Taiwan University Culture Collection (NTUPPMCC).

### ﻿DNA extraction, PCR amplification and sequencing

Genomic DNA was extracted from the 7- to 10-day-old mycelium using the EasyPure Genomic DNA Spin kit (Bioman Scientific Co., LTD., Taiwan) following the manufacturer’s protocol. PCR amplification was performed in 25 μl reaction mixture containing 12.5 μl of 2X Taq PCR Mix-RED (Bioman Scientific Co., LTD., Taiwan), 9.5 μl of ddH_2_O, 1 μl of each forward and reverse primer, and 1 μl of fungal DNA template. PCR reactions for amplification of each locus along with their respective primer pairs (See Suppl. material 1: table S1). The PCR products were examined on 1% agarose electrophoresis gel stained with BioGreen^TM^ Safe DNA Gel buffer (Bioman Scientific Co., LTD., Taiwan). Purification and sequencing were performed at Genomics Inc (New Taipei City, Taiwan) by Sanger sequencing technology. All sequences generated in this study were deposited in GenBank (See Suppl. material 1: table S2) and typification details were registered in MycoBank (Crous et al. 2004).

### ﻿Strain selection and Phylogenetic analyses

NCBI BLASTn was initially used to identify the closest matches per each strain, using ITS region to determine the family/genus level classification. Based on the BLASTn results, additional loci were sequenced, as listed in Suppl. material 1: table S1. Furthermore, for each strain, additional related sequences and DNA sequences from ex-type and additional verified strains were retrieved according to their genus by following recently published research articles. Key references and GenBank accession numbers for each locus are detailed in Suppl. material 1: table S2.The multiple Sequence alignments were obtained using MAFFT version 7 (https://mafft.cbrc.jp/alignment/server/) and manually adjusted in MEGA version 7 (Tsai et al. 2021). To understand the evolutionary relationships of the isolates obtained in this study, phylogenetic analyses were performed using maximum likelihood (ML) for both individual and multi-locus datasets and Bayesian inference (BI) for multi-locus analyses. ML analysis with 1000 bootstraps was conducted using W-IQ-TREE (http://iqtree.cibiv.univie.ac.at/) (Trifinopoulos et al. 2016). For BI, the best evolutionary model for each partition was determined using MrModeltest version 2.3 (Nylander 2004), and details were provided in See Suppl. material 1: table S3. Bayesian phylogenetic trees were generated using MrBayes version 3.2.7 (Ronquist et al. 2012). The Bayesian analysis was conducted for a total of ten million generations, automatically stopping once the mean standard deviation of split frequencies fell below 0.01. Tree samples were recorded at intervals of one thousand generations. The MCMC heated chain was set to a temperature of 0.15. To confirm when each run reached stationarity and assess the need for additional runs, the distribution of log-likelihood values was examined in Tracer v1.5 (http://beast.community/tracer). ML bootstrap values (MLB) of 70% or higher and Bayesian posterior probabilities (BPPs) of 0.95 or higher were provided at each node, whereas nodes with bootstrap values below 70% or BPPs below 0.95 were considered unresolved. FigTree v. 1.4.0 (http://tree.bio.ed.ac.uk/software/figtree/) was used to visualize the resulting phylogenetic trees and modified by using Adobe Illustrator v. 2021.

## ﻿Results

### ﻿The fungal communities of serpentine soils in Taiwan

**Figure 2.**
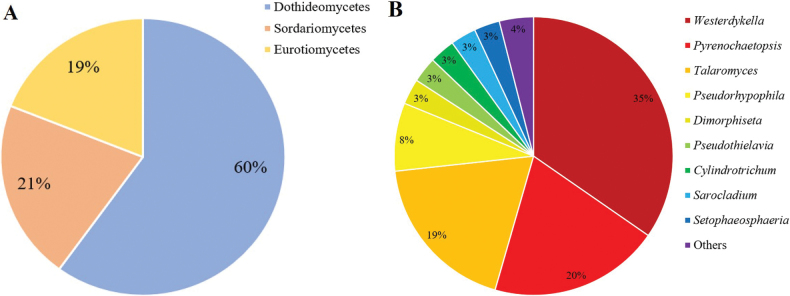
Fungal communities in six serpentine paddy soil samples, based on a total of 79 fungal strains. **A** Relative abundance at class level; **B** Relative abundance at genus level.

Using the soil suspension-plating method on ½ PDA, ½ MEA, and 1.5% water agar, a total of 154 fungal isolates were obtained. After morphological characterization, these isolates were grouped into 79 strains. Among the three media, ½ PDA yielded the highest number of strains. All 79 strains identified in this study were classified in *Ascomycota*, with 60% categorized in *Dothideomycetes*, 21% in *Sordariomycetes*, and 19% in *Eurotiomycetes* (Fig. 2A). *Westerdykella* was identified as the most abundant fungal genus accounting for 35% of the total strains, followed by *Pyrenochaetopsis* (20%), *Talaromyces* (19%), and *Pseudorhypophila* (8%) (Fig. 2B). In addition to these main genera, the study also identified several other fungal genera, including, *Dimorphiseta*, *Parasarocladium*, *Phialoparvum*, *Poaceascoma*, *Pseudothielavia*, *Pseudoxylomyces*, *Cylindrotrichum*, *Sarocladium*, and *Setophaeosphaeria*.

### ﻿Taxonomy

The phylogenetic placements and comprehensive descriptions of 33 strains belonging to 11 novel species (*Cylindrotrichumformosanum*, *Dimorphisetaformosana*, *D.serpentinicola*, *Parasarocladiumformosum*, *Phialoparvumformosanum*, *Poaceascomaserpentinum*, *Pseudorhypophilaformosana*, *Sarocladiumformosanum*, *S.serpentinicola*, *Talaromycestaiwanensis* and *Westerdykellaformosana*), as well as 46 strains belonging to 11 species (*Pseudothielaviaterricola*, *Pseudoxylomycesaquaticus*, *Pyrenochaetopsisoryzicola*, *Py.paucisetosa*, *Setophaeosphaeriamicrospora*, *Talaromycesadpressus*, *T.thailandensis*, *Westerdykellaaquatica*, *W.capitulum*, *W.dispersa*, and *W.globosa*) that are newly recorded from Taiwan or serpentine environments, are provided below.

#### ﻿*Dothideomycetes* O.E. Erikss & Winka

***Pleosporales* Luttrell ex M.E. Barr**

***Lentitheciaceae* Y. Zhang ter, C.L. Schoch, J. Fourn, Crous & K.D. Hyde**

##### ﻿ Poaceascoma

Taxon classificationAnimaliaPleosporalesLentitheciaceae

Phookamsak & K.D. Hyde

CFFACE0C-CF02-5DFC-802F-6276B8D9F9B3

###### Notes.

The genus *Poaceascoma*, first introduced by Phookamsak and Hyde (2015) in Phookamsak et al. (2015), as saprobic fungal group on *Poaceae* and *Po.helicoides* was designated as the type of the genus. Currently, 17 species are recognized in MycoBank (Accession date: March 10, 2025) for *Poaceascoma*. *Poaceascoma* species are usually characterized by semi-immersed to erumpent, globose to subglobose ascomata with short to long papillae, often surrounded by a turf-like structure (Phookamsak et al. 2015). Ascus fissitunicate, bitunicate, elongate-cylindrical usually contain eight filiform, hyaline, multi-septate ascospores. *Poaceascoma* species are mainly reported from Thailand, but have recently also been recorded from Australia, China, Hungary, South Korea and Taiwan (Hyde et al. 2018; Imrefi et al. 2024; Liu et al. 2025). These species are mainly reported from dead stems or roots of herbaceous plants (*Poaceae*) or submerged wood in freshwater ecosystems (Phookamsak et al. 2015; Luo et al. 2016; Crous et al. 2020; Boonmee et al. 2021; Zang et al. 2023).

##### ﻿ Poaceascoma serpentinum

Taxon classificationAnimaliaPleosporalesLentitheciaceae

K.W. Cheng & H.A. Ariyaw. sp. nov.

27D1C231-121F-5112-9041-23D882669F67

MB858705

Fig. 3

###### Typification.

TAIWAN • Guanshan Township, Taitung County, 23°02'17.6"N, 121°11'26.3"E, serpentine soil in rice field, 3^rd^ November 2022, K.W. Cheng, holotype, NTUPPMH 22-216 (Permanently preserved in a metabolically inactive state), ex-holotype NTUPPMCC 22-222.

###### Etymology.

Named after the serpentine soil from which the species was isolated.

###### Description.

***Sexual morph*** undetermined. ***Asexual morph Conidiophores*** submerged in WA, hyaline, flexuous, rarely straight, septate, sometimes branched, 10–25 µm. ***Conidiogenous cells*** hyaline to pale brown, holoblastic, monoblastic, terminal, occasionally intercalary, subcylindrical to swollen. ***Conidia*** hyaline to pale brown when immature, brown to dark brown when mature, ellipsoidal to broadly ellipsoidal or ovoid, finely verrucose, 3–5 septate, 31.3–48.6 µm × 12–15.7 µm (x̄ = 37.8 × 14 µm, L/W ratio = 2.72, n = 30). ***Chlamy­dospores*** brown to dark brown, dumb-bell-shaped, terminal, straight or sometimes curved, occasionally branched, 108–163 µm in length, 8–15 µm in width.

###### Culture characteristics.

Colony exhibits slow growth, reaching 35 mm diam with pale gray, fluffy to floccose surface and smooth margins. Reverse side of the colony showed a central brownish color that gradually fades into a lighter beige ring toward the edges.

###### Notes.

**Figure 3.**
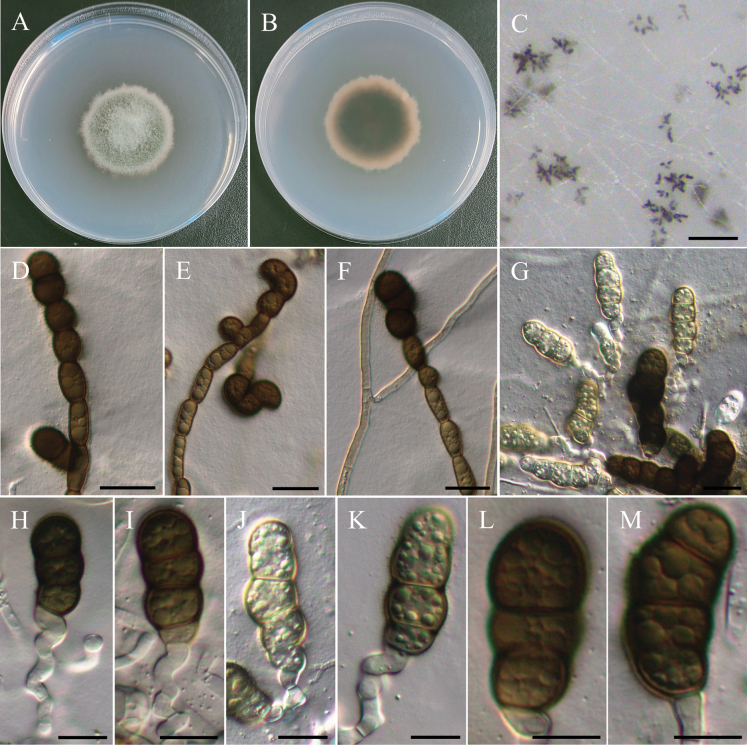
Morphology of *Poaceascomaserpentinum*NTUPPMCC 22-222. **A, B** 14-days-old colony on PDA; **C, G–M** Immature and mature conidia; **D–F** Chlamydospores. Scale bars: 0.2 mm (**C**); 20 µm (**D–G**); 10 µm (**H–M**).

**Figure 4.**
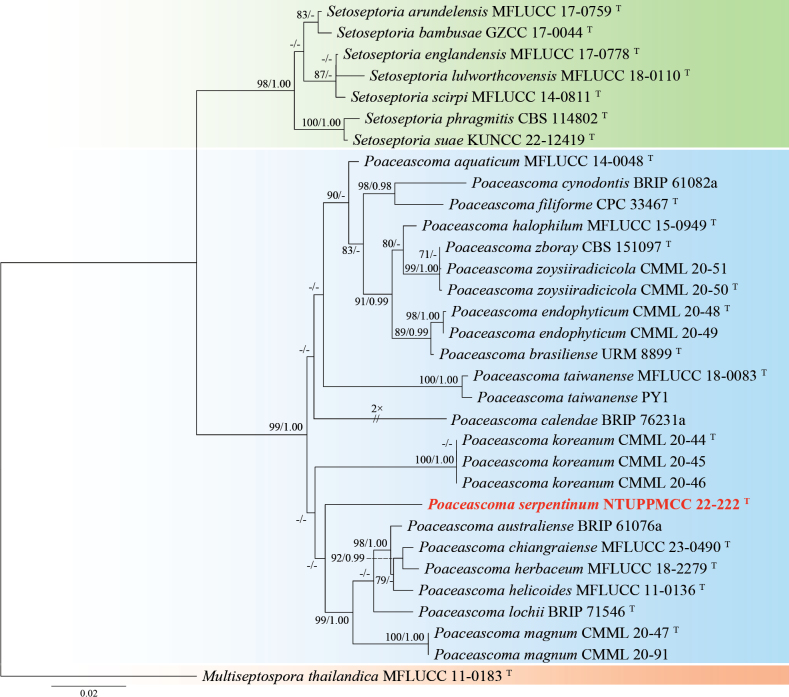
Maximum likelihood (ML) phylogenetic tree based on a concatenated dataset of ITS, LSU, SSU, and *tef-1*. In total, 32 strains representing 26 taxa were included in the concatenated dataset, with 3357 characters (ITS 606 bp, LSU 840 bp, SSU 1021 bp, and *tef-1* 890 bp) including alignment gaps. The tree was rooted with *Multiseptosporathailandica* MFLUCC 11-0183. MLB ≥ 70% and BPPs ≥ 0.95 were shown at each node; values lower than these thresholds are indicated by a hyphen (–). The scale bar indicates the number of estimated substitutions per site. The strains introduced in this study are in red and novel species are in bold. The ex-type strains are marked with ^T^.

This study describes *Poaceascomaserpentinum* as a novel fungal species based on a single strain (NTUPPMCC 22-222) isolated from serpentine soil. In our phylogenetic tree, *Poaceascomaserpentinum* forms a distinct clade within the genus *Poaceascoma* (Fig. 4). Moreover, *Po.serpentinum* exhibits significant genetic divergence from its closest relatives, the ex-type strain of *Po.koreanum* (CMML 20-44) and *Po.magnum* (CMML 20-47) with 83.6% and 87.4% identity in the ITS region (414/495 bp, including 24 gaps; 414/492, including 10 gaps). For *tef-1*, the identities are 840/890 (94.4%) and 849/890 (95.4%), respectively. Previously, species in this genus have been described solely based on their sexual stage or chlamydospore-like structures with no documented asexual stage (Zang et al. 2023; Liu et al. 2025). However, in the present study, we observed only the asexual stage of NTUPPMCC 22-222 but did not observe any sexual stage of the fungus even when carnation leaves were used as the substrate (Fig. 3). As a result, morphological comparisons between NTUPPMCC 22-222 and its closely related species are not possible. While this study establishes *Po.serpentinum* as a distinct species, future studies should aim to recover additional isolates from similar environments to further validate its phenotypic variation and ecological distribution. Notably, this study is also the first to document the asexual morphology of a *Poaceascoma* species.

#### ﻿*Longipedicellataceae* Phukhams, J. Bhat & K.D. Hyde

##### ﻿ Pseudoxylomyces

Taxon classificationAnimaliaPleosporalesLongipedicellataceae

Kaz. Tanaka & K. Hiray.

064234E6-F106-5CCE-8440-652919F59E40

###### Notes.

The genus *Pseudoxylomyces* was introduced by Tanaka and Hiray (2015) as a saprobic genus in habitat on submerged wood and typified with *Ps.elegans* (Goh et al. 1997; Tanaka et al. 2015). Currently, only two species are recognized in MycoBank (Accession date: March 10, 2025) for *Pseudoxylomyces*. To date, species of *Pseudoxylomyces* have only been described based on their asexual morphs. These species are characterized by brown, septate conidiophores that may be branched or absent and by holoblastic conidiogenous cells. The conidia are usually solitary, yellowish or orange brown to dark brown, broadly ellipsoidal or fusiform with several transverse septa of thick-walled, with paler end cells and without sheath or appendages (Tanaka et al. 2015; Dong et al. 2020). However, this group has a wide distribution and has been reported from Australia, Brazil, Hong Kong, India, Japan, Seychelles, Thailand and USA (Dong et al. 2020). The majority of the isolates reported for this genus were derived from the submerged wood in the aquatic environment (Dong et al. 2020).

##### ﻿ Pseudoxylomyces aquaticus

Taxon classificationAnimaliaPleosporalesLongipedicellataceae

W. Dong, H. Zhang & K.D. Hyde (2020)

F25A3AAE-047C-55DC-8D2A-D3EEA8B1F49F

MB557916

Fig. 5

###### Description.

***Sexual morph*** undetermined. ***Asexual morph*** Conidia produced on carnation leaves and on the surface or submerged in WA. ***Conidiophores*** short (7–13.5 µm) or absent. ***Conidiogenous cells*** holoblastic. ***Conidia*** solitary, orange-brown when immature, turn brown in mature, occasionally with paler end cells in pale brown, broadly fusiform, most 5 thick and obvious septa (few 3–4 septa), rough, thick-walled, 37.6–50 µm × 11.8–17 µm (x̄ = 44 × 14.5 µm, L/W ratio = 3.05, n = 20).

###### Culture characteristics.

Colony reaching 30 mm diam with dark grayish-brown in the center, pale brown to gray in margin, velvety, rough surface, entire edge, and similar to reverse side of the colony.

###### Material examined.

TAIWAN • Wanrung Township, Hualien County, 23°42'40.3"N, 121°24'48.2"E, serpentine soil in rice field, 2^nd^ November 2022, K.W. Cheng, living culture NTUPPMCC 22-223.

###### Notes.

**Figure 5.**
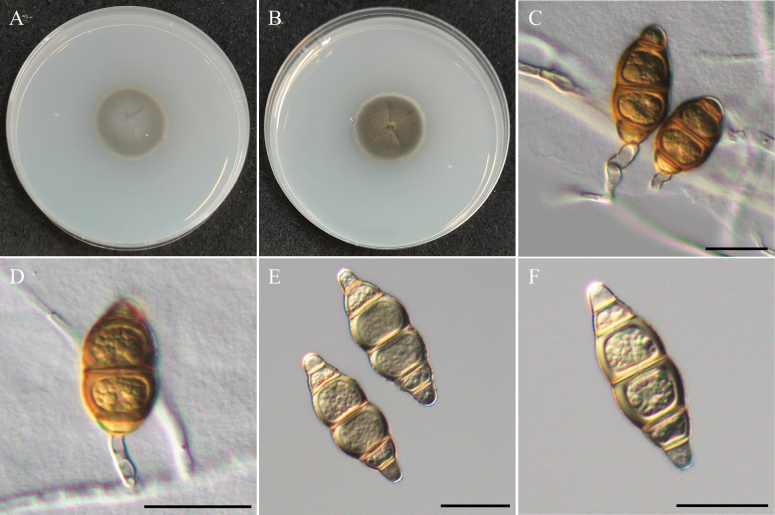
Morphology of *Pseudoxylomycesaquaticus*NTUPPMCC 22-223. **A, B** 14-days-old colony on PDA; **C, D** Immature conidia and conidiophores; **E, F** Conidia. Scale bars: 20 µm (**C–F**).

**Figure 6.**
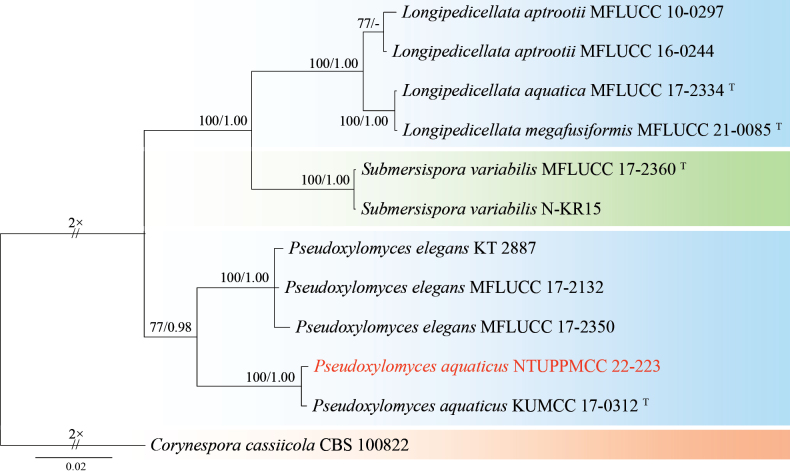
Maximum likelihood (ML) phylogenetic tree based on a concatenated dataset of ITS, LSU, SSU, and *tef-1*. In total, 12 strains representing seven taxa were included in the concatenated dataset, with 2920 characters (ITS 526 bp, LSU 845 bp, SSU 643 bp, and *tef-1* 906 bp) including alignment gaps. The tree was rooted with *Corynesporacassiicola* CBS 100822. MLB ≥ 70% and BPPs ≥ 0.95 were shown at each node; values lower than these thresholds are indicated by a hyphen (–). The scale bar indicates the number of estimated substitutions per site. The strains introduced in this study are in red and novel species are in bold. The ex-type strains are marked with ^T^.

*Pseudoxylomycesaquaticus* was previously documented on submerged wood in Thailand. Our study recovered a single strain (NTUPPMCC 22-223) that clustered in a strongly supported clade (100%/1.00) with the ex-type strain (KUMCC 17-0312) established by Dong et al. (2020), confirming its identity as *Ps.aquaticus* (Fig. 6). In this study, we observed the immature conidia with 3 to 4 septa showing more orange-brown in color (Fig. 5C, D), a feature not described in previous study (Dong et al. 2020). In addition, this is the first discovery of the *Pseudoxylomyces* species in Taiwan.

#### ﻿*Pyrenochaetopsidaceae* Valenz.-Lopez, Crous, J.F. Cano, Guarro & Stchigel

##### ﻿ Pyrenochaetopsis

Taxon classificationAnimaliaPleosporalesPyrenochaetopsidaceae

Gruyter, Aveskamp & Verkley

1B7CBAFD-7FB9-54BC-935E-9D212876DBCD

###### Notes.

The genus *Pyrenochaetopsis* was introduced by de Gruyter et al. (2010), as a saprobe on *Poaceae* and typified with *Py.leptospora*. Currently, 27 species are recognized in MycoBank (Accession date: March 10, 2025) for *Pyrenochaetopsis*. *Pyrenochaetopsis* species have been described based on either their sexual or asexual stages or both. The asexual stage is characterized by olivaceous to olivaceous-black or pale brown to brown, solitary to confluent, superficial or submerged, globose to subglobose pycnidial conidiomata that may have a non-papillate or papillate ostiolar neck. The pycnidial wall, composed of textura angularis or textura globulosa, is pseudoparenchymatous and often bears setae. Conidiogenous cells are phialidic, hyaline, and born on acropleurogenous conidiophores. The conidia are aseptate, hyaline, ovoid to cylindrical or oblong, smooth-walled and guttulate (de Gruyter et al. 2010; Valenzuela-Lopez et al. 2018; Samarakoon et al. 2024). The sexual stage is represented by brown to dark brown or dark grey to black, globose to subglobose, solitary or scattered, superficial or immersed to semi-immersed ascomata with short papillate ostiole covered with reddish-brown setae, and a pseudoparenchymatous peridium composed of dark brown, ***textura angularis*** to ***textura prismatica***. Asci are cylindric-clavate, fissitunicate, bitunicate and eight-spored. Ascospores are hyaline to pale brown, yellowish-brown, or yellowish-gray, fusiform to oblong in shape, smooth-walled, and three- to four-septate (Mapook et al. 2020; Phookamsak et al. 2022; Absalan et al. 2024). These species are widely distributed around the world and can be found in diverse ecological niches, functioning as saprobes, endophytes, or pathogens (de Gruyter et al. 2010; Surono et al. 2023). However, most of the species are associated with plant debris, soil, or dung while some have also been discovered on opportunistic infections in nematode cysts or human tissues (Valenzuela-Lopez et al. 2018).

##### ﻿ Pyrenochaetopsis paucisetosa

Taxon classificationAnimaliaPleosporalesPyrenochaetopsidaceae

N. Valenzuela-Lopez, J.F. Cano, J. Guarro & A.M. Stchigel (2018)

07D4131E-E4B9-5BDB-9D2E-6F760E4AB9D1

MB819766

Fig. 7

###### Description.

***Sexual morph*** undetermined. ***Asexual morph Conidiomata*** 128–219 µm, pycnidial, globose to subglobose, dark brown, ostiolate, with dark brown, septate setae, superficial on WA and carnation leaves. ***Pycnidial wall*** textura angularis, septate setae, brown, pseudoparenchymatous cells. ***Conidiogenous cells*** hyaline, phialidic, smooth-walled, 3.6–5.0 µm × 2.9–4.1 µm (x̄ = 4.1 × 3.5 µm, n = 30). ***Conidia*** hyaline, cylindrical to ellipsoidal, aseptate, with 2 small guttules, 3.7–4.7 µm × 1.5–2.1 µm (x̄ = 4.2 × 1.8 µm, L/W ratio = 2.4, n = 30).

###### Culture characteristics.

Colony exhibit slow growth, reaching 28 mm diam with pale gray, floccose surface, smooth margins, and medium-gray on the reverse side of the colony.

###### Material examined.

TAIWAN • Wanrung Township, Hualien County, 23°42'41.2"N, 121°24'41.8"E, serpentine soil in rice field, 2^nd^ November 2022, K.W Cheng, living culture NTUPPMCC 22-227.

###### Notes.

**Figure 7.**
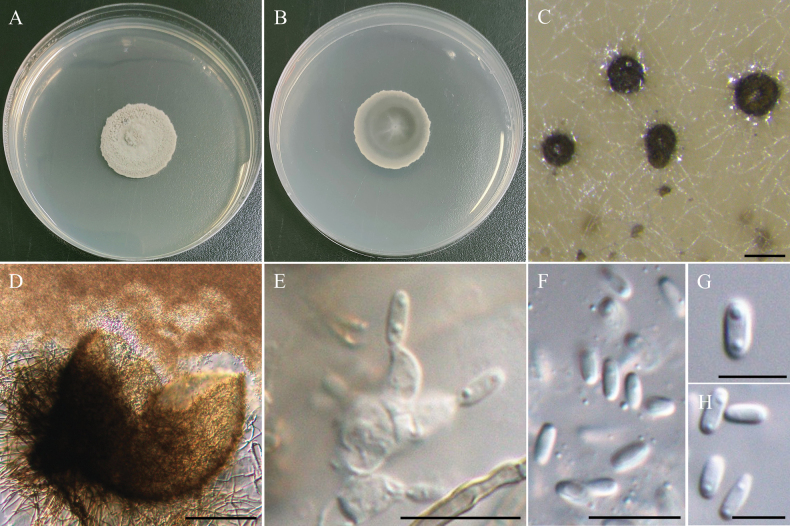
Morphology of *Pyrenochaetopsispaucisetosa*NTUPPMCC 22-227. **A, B** 14-days-old colony on PDA; **C** Conidiomata; **D** Squashed conidiomata; **E** Conidiogenous cells; **F–H** Conidia. Scale bars: 0.2 mm (**C**); 0.1 mm (**D**); 10 µm (**E, F**); 5 µm (**G, H**).

**Figure 8.**
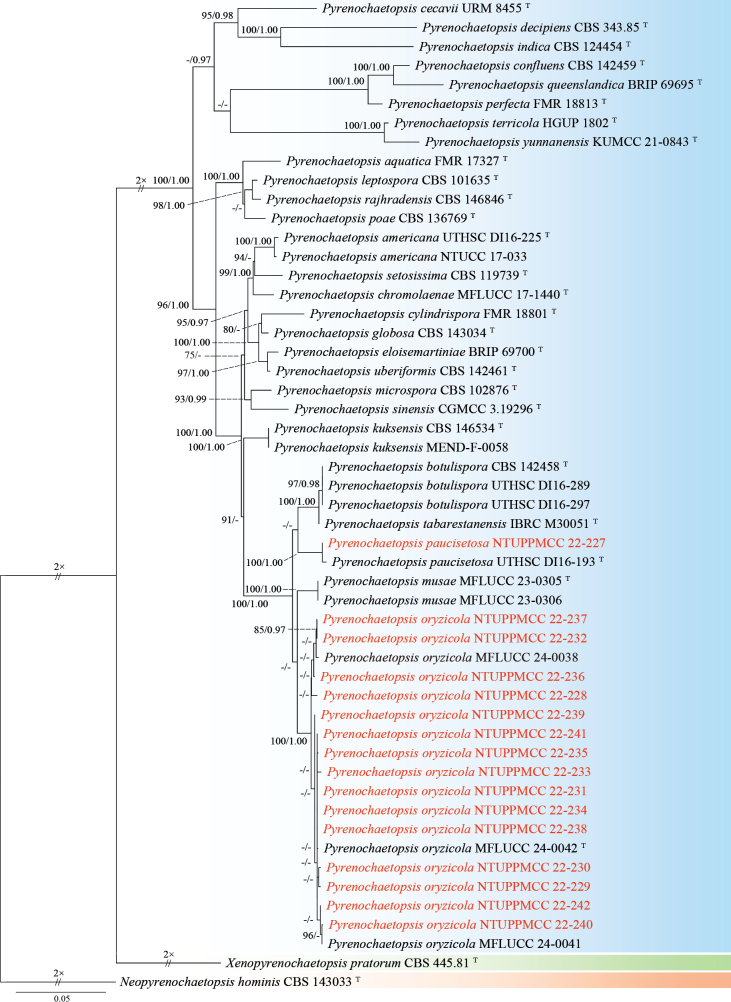
Maximum likelihood (ML) phylogenetic tree based on a concatenated dataset of ITS, LSU, *rpb2*, and *tub2*. In total, 52 strains representing 29 taxa were included in the concatenated dataset, with 2453 characters (ITS 451 bp, LSU 845 bp, *rpb2* 815 bp, and *tub2* 342 bp) including alignment gaps. The tree was rooted with *Neopyrenochaetopsishominis* CBS 143033. MLB ≥ 70% and BPPs ≥ 0.95 were shown at each node; values lower than these thresholds are indicated by a hyphen (–). The scale bar indicates the number of estimated substitutions per site. The strains introduced in this study are in red and novel species are in bold. The ex-type strains are marked with ^T^.

In the present study, strain NTUPPMCC 22-227 identified as *Pyrenochaetopsispaucisetosa*, clustering in a strongly supported clade (100%/1.00) with the type strain of *Py.paucisetosa* (UTHSC DI16-193) in multi-gene phylogeny analysis (Fig. 8; Valenzuela-Lopez et al. 2018). Previous reports indicate that *Py.paucisetosa* has been isolated from a human toe nail in USA (Valenzuela-Lopez et al. 2018) and from freshwater sediment in Korea (Goh et al. 2020). The culture characteristics of NTUPPMCC 22-227 on PDA were similar to those of the ex-holotype of *Py.paucisetosa* (UTHSC DI16-193). However, the conidia of *Py.paucisetosa*NTUPPMCC 22-227 in our study were more elongated than previous reports (x̄ = 4.2 × 1.8 µm versus 3.6 × 1.9 µm) (Fig. 7; Valenzuela-Lopez et al. 2018; Goh et al. 2020). Furthermore, this is the first record of *Py.paucisetosa* in Taiwan, as well as its first occurrence in paddy field soil.

##### ﻿ Pyrenochaetopsis oryzicola

Taxon classificationAnimaliaPleosporalesPyrenochaetopsidaceae

S. Absalan, S. Lumyong & K.D. Hyde (2024)

7BF74E62-A94B-5CEF-ADFB-8866F5297F48

MB902535

Fig. 9

###### Description.

***Sexual morph*** undetermined. ***Asexual morph*** Sporulation difficult on PDA and MEA, conidiomata produced on carnation leaves and on the surface or submerged in WA. ***Conidiomata*** 140–196 µm, pycnidial, brown, globose to subglobose, ostiolate, superficial on WA and carnation leaves, with dark brown, septate setae. ***Pycnidial wall*** textura angularis to globulosa, brown, pseudoparenchymatous cells. ***Conidiogenous cells*** hyaline, phialidic, smooth-walled, and hard to distinguish from the pycnidial wall 3.3–5.6 µm × 3.0–4.6 µm (x̄ = 4.4 × 3.5 µm, n = 30). ***Conidia*** hyaline, cylindrical to ellipsoidal, aseptate, with 2 small but obvious guttules, 3.6–5.1 µm × 1.7–2.5 µm (x̄ = 4.3 × 2.2 µm, L/W ratio = 2.03, n = 50).

###### Culture characteristics.

Colony reaching 45 mm diam with greenish-gray, flat, velvety to floccose surface and smooth margins, and similar to reverse side of the colony.

###### Material examined.

TAIWAN • Wanrung Township, Hualien County, 23°42'40.3"N, 121°24'48.2"E, serpentine soil in rice field, 2^nd^ November 2022, K.W Cheng, living culture NTUPPMCC 22-228 to 242.

###### Notes.

**Figure 9.**
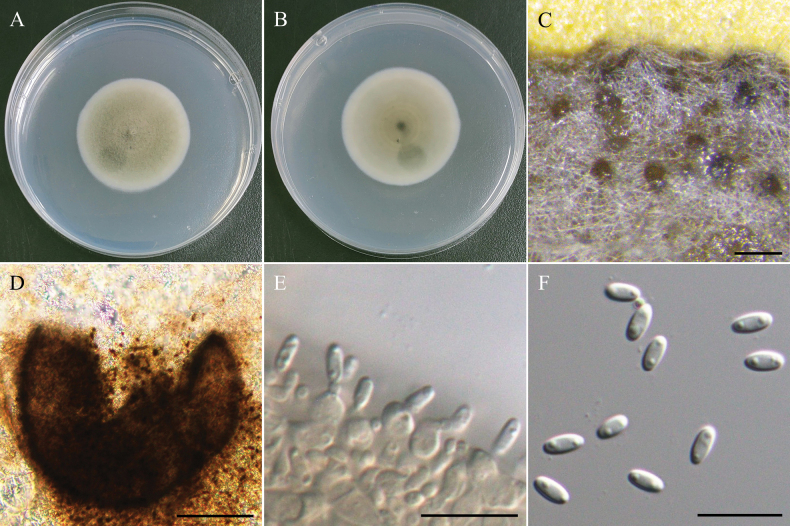
Morphology of *Pyrenochaetopsisoryzicola*NTUPPMCC 22-229. **A, B** 14-days-old colony on PDA; **C** Conidiomata; **D** Squashed conidiomata; **E** Conidiogenous cells; **F** Conidia. Scale bars: 0.5 mm (**C**); 0.1 mm (**D**); 10 µm (**E, F**).

*Pyrenochaetopsisoryzicola* was originally reported from dead panicles of *Oryzasativa* in paddy fields in Thailand (Absalan et al. 2024). In the present, in both single- and multi-gene phylogenetic analyses showed that strains from NTUPPMCC 22-228 to 242 isolated in this study, grouped with the clade containing the ex-type strain of *Py.oryzicola* (MFLUCC 24-0042) (Fig. 8). Consequently, these strains were identified as *Pyrenochaetopsisoryzicola*. Their culture characteristics on PDA, along with conidial morphology, were consistent with those of the epitype of *Py.oryzicola* (MFLU 24-0319). However, the conidiogenous cells of *Py.oryzicola*NTUPPMCC 22-229 isolated in our study were larger than those reported in the previous study (x̄ = 4.5 × 3.5 µm versus 1.5 × 1.0 µm) (Fig. 9; Absalan et al. 2024).

#### ﻿*Phaeosphaeriaceae* M.E. Barr

##### ﻿ Setophaeosphaeria

Taxon classificationAnimaliaPleosporalesPhaeosphaeriaceae

Crous & Y. Zhang ter

35E4AD70-AAE7-567A-88C8-68C211AFEFAD

###### Notes.

Crous and Zhang (2014) introduced the genus *Setophaeosphaeria* to accommodate *Se.hemerocallidis* isolated from leaf of *Hemerocallisfulva* (Crous et al. 2014). Currently, eight species are recognized in MycoBank (Accession date: March 10, 2025) for *Setophaeosphaeria*. *Setophaeosphaeria* species have been recorded from both sexual and asexual stage. Conidiomata are pycnidial, brown, globose, immersed or erumpent with central ostiole. Pycnidial wall is brown with 2–3 or 6–8 layers of textura angularis, pale brown or brown. Setae brown or pale brown, septate, unbranched, flexuous, smooth with obtuse ends. Conidiophores are reduced to conidiogenous cells. Conidiogenous cells are hyaline, ampulliform, smooth, proliferating several times percurrently at apex, lining the inner cavity. Conidia are hyaline, cylindrical or subcylindrical, smooth, guttulate, aseptate with obtuse ends (Crous et al. 2014, 2017, 2018b; Zhang et al. 2020). Ascomata are globose, immersed on host, subepidermal with central ostiole consists of 2–3 layers of brown textura angularis peridium. Asci are eight-spored, bitunicate, subcylindrical to narrowly ellipsoidal, stipitate containing pale brown or hyaline, fusoid-ellipsoidal, aseptate or septate, smooth ascospores (Crous et al. 2014, 2018b). *Setophaeosphaeria* species are widely distributed and have been reported from Australia, Brazil, China, Italy, Netherlands, and South Korea (Crous et al. 2017, 2018b; Choi et al. 2024; Liu et al. 2024). However, most of these strains were isolated from the leaf spots and branch dieback (Liu et al. 2024).

##### ﻿ Setophaeosphaeria microspora

Taxon classificationAnimaliaPleosporalesPhaeosphaeriaceae

Z.F. Zhang & L. Cai (2020)

E096DEC3-32C2-54D1-89D0-2AB1B69CE621

MB556393

Fig. 10

###### Description.

***Sexual morph*** undetermined. ***Asexual morph Conidiomata*** 170–240 µm, pycnidial, brown, globose, ostiolate, submerged or superficial on PDA. ***Setae*** brown, straight to slightly curved, thick-walled, smooth, septate, up to 160–250 µm long, 3.5–4.0 µm wide at broadest part. ***Pycnidial wall*** textura angularis to globulosa, brown to dark brown, multi-layers. ***Conidiogenous cells*** hyaline, subglobose, smooth-walled, 3.3–4.5 µm × 2.3–3.5 µm (x̄ = 3.9 × 2.9 µm, n = 15). ***Conidia*** hyaline, cylindrical, obtuse ends, aseptate, with 2 small but obvious guttules, 3.2–4.0 µm × 1.3–1.7 µm (x̄ = 3.7 × 1.4 µm, L/W ratio = 2.54, n = 30).

###### Culture characteristics.

Colony reaching 35 mm diam with dark grayish-green in center, beige in margin, velvety, entire edge, and similar to reverse side of the colony.

###### Material examined.

TAIWAN • Guanshan Township, Taitung County, 23°02'12.8"N, 121°11'22.0"E, serpentine soil in rice field, 2^nd^ November 2022, K.W Cheng, living culture NTUPPMCC 22-225 and NTUPPMCC 22-226.

###### Notes.

**Figure 10.**
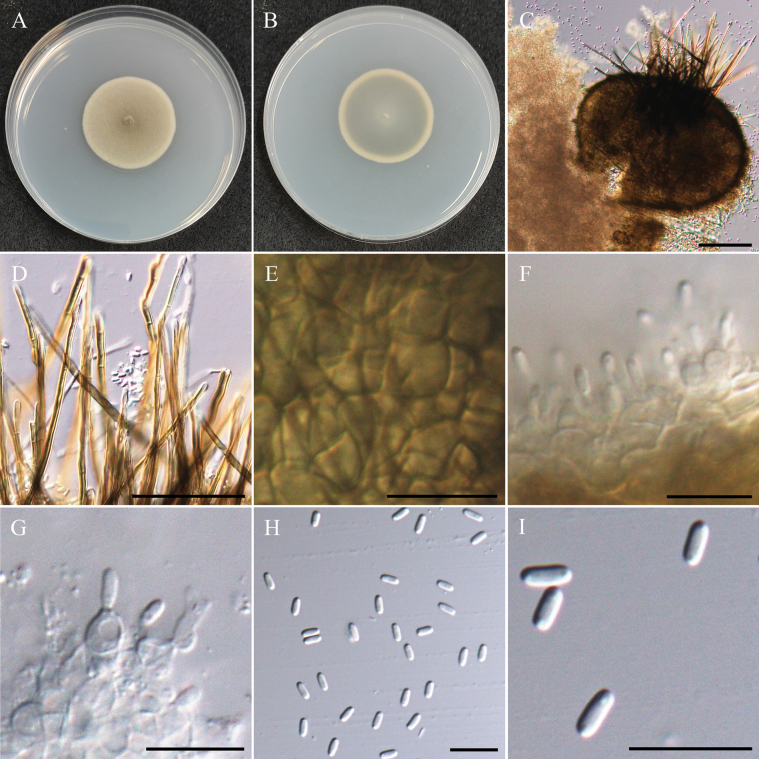
Morphology of *Setophaeosphaeriamicrospora*NTUPPMCC 22-225. **A, B** 14-days-old colony on PDA; **C** Squashed Conidiomata; **D** Setae; **E** Pycnidial wall; **F, G** Conidiogenous cells; **H, I** Conidia. Scale bars: 0.1 mm (**C**); 50 µm (**D**); 10 µm (**E–I**).

**Figure 11.**
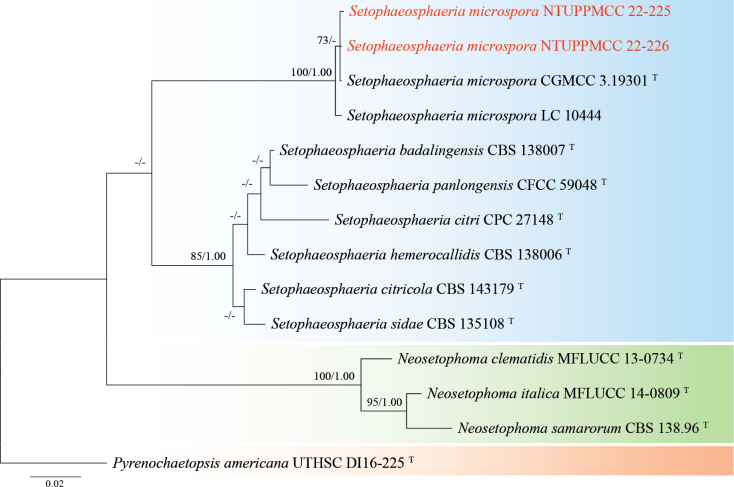
Maximum likelihood (ML) phylogenetic tree based on a concatenated dataset of ITS, LSU, and *tub2*. In total, 14 strains representing 11 taxa were included in the concatenated dataset, with 1534 characters (ITS 482 bp, LSU 773 bp, and *tub2* 279 bp) including alignment gaps. The tree was rooted with *Pyrenochaetopsisamericana* UTHSC DI16-225. MLB ≥ 70% and BPPs ≥ 0.95 were shown at each node; values lower than these thresholds are indicated by a hyphen (–). The scale bar indicates the number of estimated substitutions per site. The strains introduced in this study are in red and novel species are in bold. The ex-type strains are marked with ^T^.

The strains named NTUPPMCC 22-225 and NTUPPMCC 22-226 isolated in the present study clustered with ex-type strain *Setophaeosphaeriamicrospora* CGMCC 3.19301 with high statistical support, confirming their identification as *Se.microspora* (Fig. 11). However, *Se.microspora* (NTUPPMCC 22-225) exhibited smaller conidiogenous cells than the type strain CGMCC 3.19301 (3.5–4.5 µm × 2.5–3.5 µm versus 7.0–10.0 µm × 2.5–4.0 µm) (Fig. 10; Zhang et al. 2020). This study represents the first report of *Setophaeosphaeria* in Taiwan.

#### ﻿*Sporormiaceae* Munk

##### ﻿ Westerdykella

Taxon classificationAnimaliaPleosporalesSporormiaceae

Stolk

6A2EB641-729F-54AF-A652-725D154AD082

###### Notes.

Stolk (1955) introduced *Westerdykella* and typified the genus with *W.ornata*, which was isolated from soil in Mozambique. Currently, 14 *Westerdykella* species are listed in MycoBank (Accession date: March 10, 2025), which have been recorded worldwide on a wide range of substrates including dung, plant debris, soil, and water (Chethana et al. 2021). However, there have also been rare reports of *W.dispersa* isolated from neutropenic and critically burned patients in hospitals (Sue et al. 2014; Lipovy et al. 2018). Most species in *Westerdykella* have been described based on the presence of the sexual morph. However, some species, such as *W.dispersa*, form both the sexual and asexual morphs in the same culture medium (Clum 1955). *Westerdykella* species form superficial or submerged, globose to subglobose, or globose to irregular-elongate, olive to olive-black, or brown to dark brown, conidiomata with ostiole. The conidia are hyaline, globose to oval or pyriform, and born on simple, short conidiophores (Rai and Tewari 1963; Zimowska 2007; Chethana et al. 2021). They form superficial or submerged, globose to subglobose in ascomata sexual stage. The peridium of the ascoma is single-layered, consisting of brown textura angularis. Asci are globose to subglobose or pyriform, hyaline when immature, becoming brown at maturity. Ascospores vary in shapes (reniform, globose, or ellipsoidal) and are hyaline to pale brown or brown, guttulate, with ascospore segments separating as soon as they become visible (Rai and Tewari 1963; Ito and Nakagiri 1995; Ebead et al. 2012; Song et al. 2020; Goh et al. 2021).

##### ﻿ Westerdykella aquatica

Taxon classificationAnimaliaPleosporalesSporormiaceae

H.Y. Song & D.M. Hu (2020)

017F0F72-09EB-5F4D-B2D4-F2934622AEDD

MB825645

Fig. 12

###### Description.

***Sexual morph Cleistothecia*** superficial or submerged on central region of PDA, 150–291 µm diam, globose to subglobose, glabrous, dirty gray when immature, black when mature. ***Peridium*** single-layered, brown, translucent, membranous, angular cells. ***Asci*** subglobose to ovoid, hyaline when immature, brown when mature, 32-spored, 12.2–16.3 µm × 10.7–14.7 µm (x̄ = 14.3 × 12.6 µm, L/W ratio = 1.15, n = 30). ***Ascospores*** ellipsoidal, smooth, subhyaline to light brown, 1 to 2 guttules, no germ-slits, 4.7–5.9 µm × 2.2–3.3 µm (x̄ = 5.1 × 2.8 µm, L/W ratio = 1.87, n = 50). ***Asexual morph*** undetermined.

###### Culture characteristics.

Colony exhibits rapid growth, reaching 90 mm daim with a slightly diffused edge, flat and fluffy, predominantly creamy white, with a central region transitioning to pale yellow, reverse yellow to dark yellow in the central region due to the presence of cleistothecia.

###### Material examined.

TAIWAN • Guanshan Township, Taitung County, 23°02'17.6"N, 121°11'26.3"E, serpentine soil in rice field, 3^rd^ November 2022, K.W. Cheng, living culture NTUPPMCC 22-248 to 251.

###### Notes.

**Figure 12.**
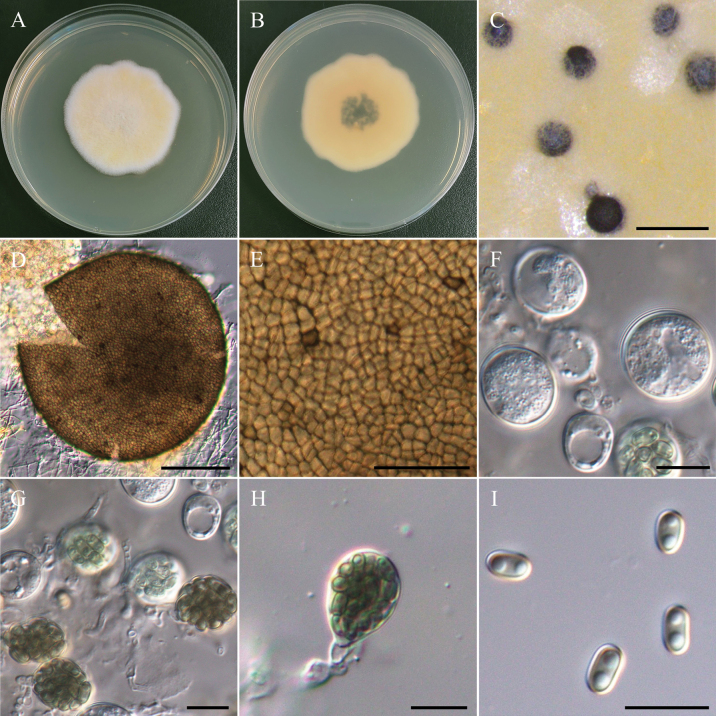
Morphology of *Westerdykellaaquatica*NTUPPMCC 22-251. **A, B** 14-days-old colony on PDA; **C** Ascomata; **D** Squashed ascomata; **E** Peridium; **F, G** Immature asci; **H** Ascus; **I** Ascospores. Scale bars: 0.5 mm (**C**); 50 µm (**D**); 25 µm (**E**); 10 µm (**F–I**).

**Figure 13.**
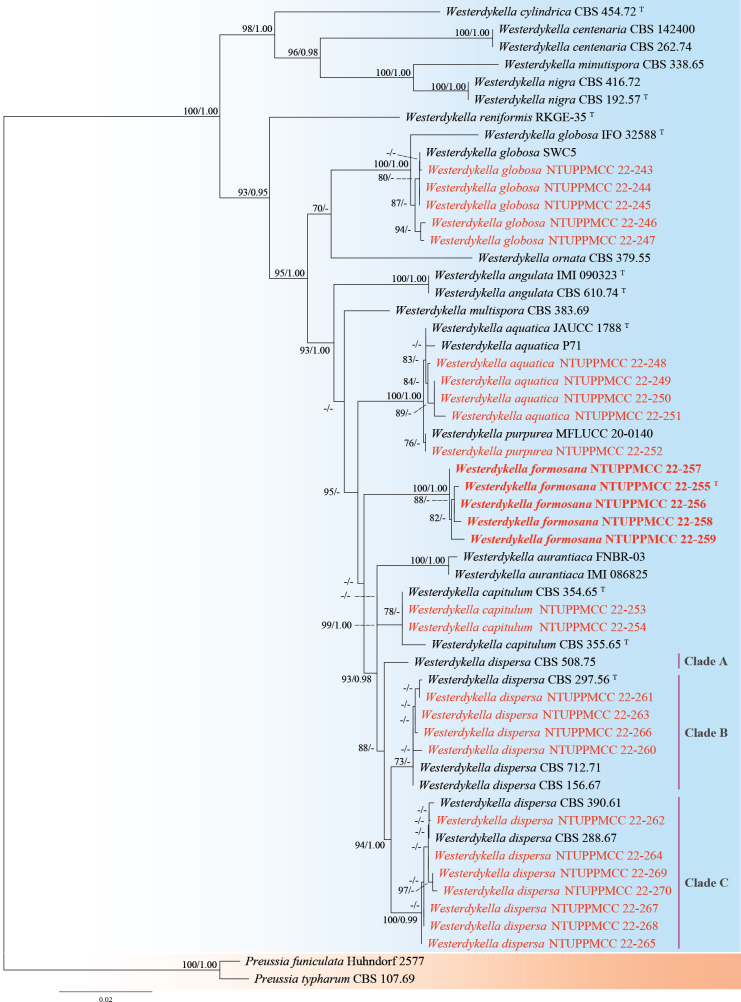
Maximum likelihood (ML) phylogenetic tree based on a concatenated dataset of ITS, LSU, and *tub2*. In total, 56 strains representing 17 taxa were included in the concatenated dataset, with 2314 characters (ITS 459 bp, LSU 865 bp, and *tub2* 990 bp) including alignment gaps. The tree was rooted with *Preussiafuniculata* Huhndorf 2577 and *P.typharum* CBS 107.69. MLB ≥ 70% and BPPs ≥ 0.95 were shown at each node; values lower than these thresholds are indicated by a hyphen (–). The scale bar indicates the number of estimated substitutions per site. The strains introduced in this study are in red and novel species are in bold. The ex-type strains are marked with ^T^.

*Westerdykellaaquatica* has been reported from rice field mud and stems of *Acoruscalamus* in China (Song et al. 2020), river sediment in Korea (Goh et al. 2021), and *Polygonumacuminatum* Kunth root in Brazil (Pietro-Souza et al. 2017; Senabio et al. 2023). In the present study, multi-gene phylogeny indicated that our strains NTUPPMCC 22-248 to 251 grouped with the clade representing *W.aquatica* (Fig. 13). Especially, similar to previous studies (Song et al. 2020; Goh et al. 2021), only the sexual stage was observed for all the strains identified as *W.aquatica* in the present study (Fig. 12). Distinguishing *W.aquatica* from its phylogenetically closely related species, *W.purpurea* based solely on sexual-stage morphology can be challenging. This is the first report of *W.aquatica* in Taiwan.

##### ﻿ Westerdykella capitulum

Taxon classificationAnimaliaPleosporalesSporormiaceae

J.de Gruyter, M.M. Aveskamp & J.Z. Groenewald (2012)

7EB7A158-2868-5E5B-8E0E-9BBE44F8474F

MB564801

Fig. 14

###### Description.

***Sexual morph*** undetermined. ***Asexual morph Conidiomata*** 95–198 µm, globose, subglobose to irregular due to fusion of two or more, glabrous, dark brown, ostiolate, mostly superficial, some submerged in PDA. ***Conidia*** borne terminally in camel brown gelatinous mass, ellipsoidal, some globose, smooth, hyaline, 1 to 2 guttules, 3.0–4.1 µm × 2.4–3.4 µm (x̄ = 3.5 × 2.8 µm, L/W ratio = 1.3, n = 50.

###### Culture characteristics.

Colony exhibit rapid growth, reaching 90 mm daim with a uniform surface and smooth margins, forming a concentric pattern. The central region appears light grayish-brown due to dense conidiomata, while the edges exhibit a translucent beige hue.

###### Material examined.

TAIWAN • Wanrung Township, Hualien County, 23°42'40.3"N, 121°24'48.2"E, serpentine soil in rice field, 2^nd^ November 2022, K.W. Cheng, living culture NTUPPMCC 22-253 and NTUPPMCC 22-254.

###### Notes.

**Figure 14.**
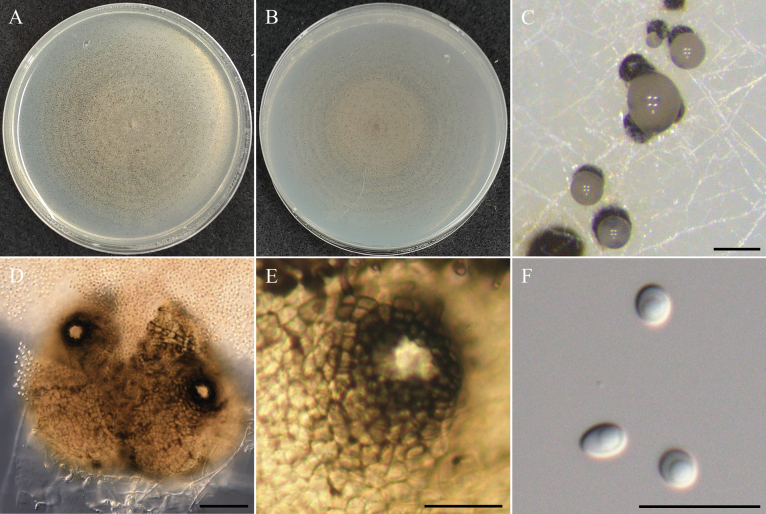
Morphology of *Westerdykellacapitulum*NTUPPMCC 22-253. **A, B** 14-days-old colony on PDA; **C** Conidiomata; **D** Squashed conidiomata; **E** Ostiolate; **F** Conidia. Scale bars: 0.2 mm (**C**) 20 µm; (**D, E**); 10 µm (**F**).

*Westerdykellacapitulum* has been reported from various environments, including saline soil in India (Pawar et al. 1967), root of motherwort (*Leonuruscardiaca*) in Poland (Zimowska 2007), and mudflat in Korea (Genomic data) (Heo et al. 2019). In our study, two strains (NTUPPMCC 22-253 and 22-254) were recovered and clustered in a strongly supported clade (99%/1.00) with reference strains CBS 354.65 and CBS 355.65, confirming their identity as *W.capitulum* (Fig. 13). Morphological characters of the representative strain of *W.capitulum*NTUPPMCC 22-253 used in this study are similar to *W.capitulum* reported by de Gruyter et al. (2012). Similar to previous studies, only the asexual stage was observed for strains identified as *W.capitulum* in the present study (Fig. 14; Pawar et al. 1967; de Gruyter and Noordeloos 1992; Zimowska 2007). This is the first report of *W.capitulum* in Taiwan.

##### ﻿ Westerdykella dispersa

Taxon classificationAnimaliaPleosporalesSporormiaceae

K. Cejp & A.A. Milko (1964)

1F657000-3D13-5D14-9BF7-97E68981B779

MB341019

Fig. 15

###### Description.

***Sexual morph Cleistothecia*** 187–296 µm diam, globose to subglobose, glabrous, dark brown to black when mature, superficial or submerged in PDA. ***Peridium*** single-layered, light brown, translucent, membranous, angular cells. ***Asci*** subglobose to ovoid, hyaline when immature, brown when mature, 32-spored, 11.0–13.6 µm × 9.7–11.8 µm (x̄ = 12.3 × 10.8 µm, L/W ratio = 1.14, n = 30). ***Ascospores*** ellipsoidal, smooth, subhyaline to light brown, 2 guttules, no germ-slits, 1.8–2.7 µm × 3.7–4.5 µm (x̄ = 2.3 × 4.0 µm, L/W ratio = 1.82, n = 50). ***Asexual morph Conidiomata*** abundant on PDA at 25 °C, 7 days post-inoculation, 44–88 µm, globose, subglobose to irregular due to fusion of two or more, glabrous, brown, ostiolate, superficial. ***Conidia*** borne terminally in camel brown gelatinous mass, ellipsoidal, subglobose, some pyriform, smooth, hyaline, 0 to 2 guttules, 1.8–2.7 µm × 3.1–4.0 µm (x̄ = 2.1 × 3.3 µm, L/W ratio = 1.62, n = 50).

###### Culture characteristics.

Colony exhibit rapid growth, reaching 80 mm daim with slightly diffuse and beige margins, yellow to pale orange pigmentation radiating outward in a concentric ring pattern, texture velvety to slightly cottony. The cleistothecia and abundant conidiomata caused the center to appear black or dark yellow.

###### Material examined.

TAIWAN • Guanshan Township, Taitung County, 23°02'12.8"N, 121°11'22.6"E, serpentine soil in rice field, 3^rd^ November 2022, K.W. Cheng, living culture NTUPPMCC 22-260 to 270.

###### Notes.

**Figure 15.**
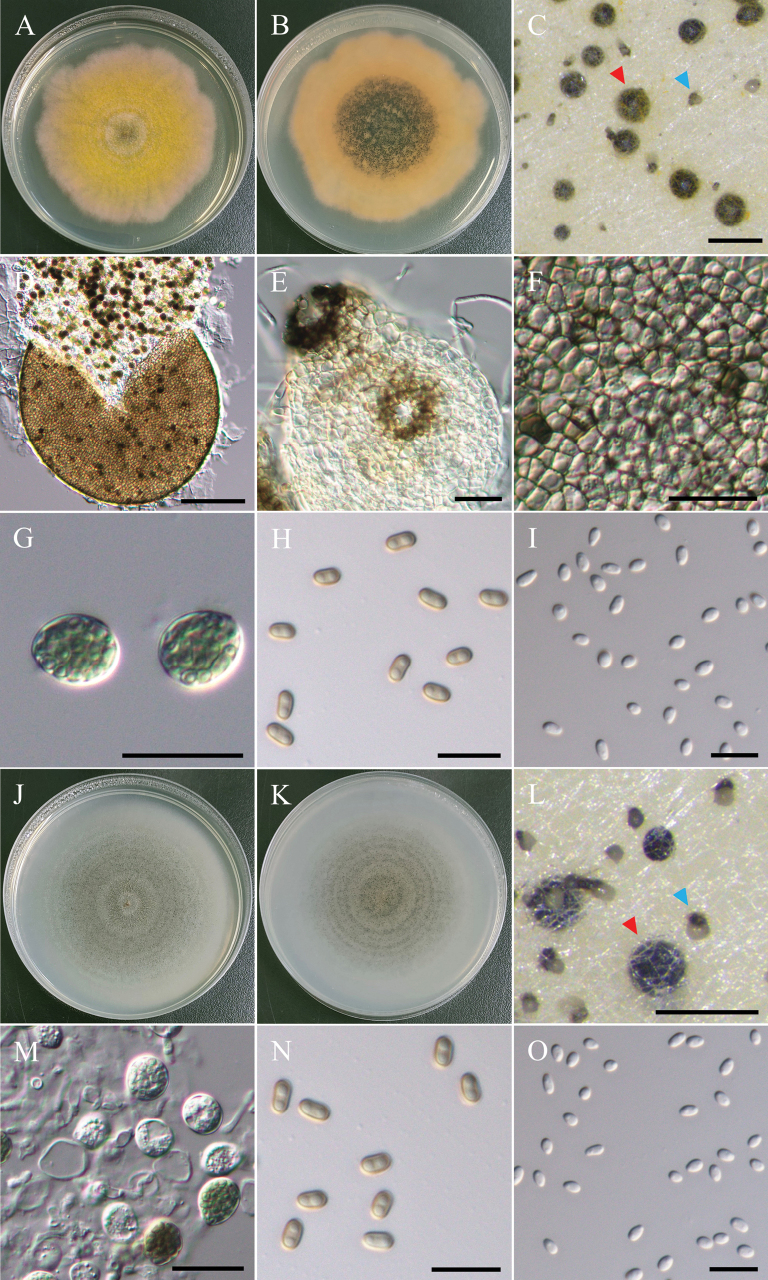
Morphology of *Westerdykelladispersa*NTUPPMCC 22-266. **A, B** 14-days-old colony on PDA; **C** Ascomata (red arrow) and conidiomata (blue arrow); **D** Squashed ascomata; **E** Conidiomata and ostiolate; **F** Peridium; **G** Asci; **H** Ascospores; **I** Conidia. Morphology of *Westerdykelladispersa*NTUPPMCC 22-269. **J, K** 14-days-old colony on PDA; **L** Ascomata (red arrow) and conidiomata (blue arrow); **M** Ascospores; **N** Conidia. Scale bars: 0.5 mm (**C, L**); 0.1 mm (**D**); 20 µm (**E–G, M**); 10 µm (**H, I, N, O**).

*Westerdykelladispersa* have a global distribution and have been isolated from diverse substrates, including soil from the Netherlands and Nigeria (Arenal et al. 2007), freshwater ecosystem sediments in Brazil and Korea (da Silva et al. 2003; Goh et al. 2021), marine sediments in China and Spain (Xu et al. 2017; Guerra-Mateo et al. 2024), as endophytes of *Phragmitesaustralis* in Italy (Angelini et al. 2012), and in rare cases, isolated from a neutropenic patient (Clum 1955; Sue et al. 2014). In the multi-locus phylogenetic analysis conducted in this study, strains identified as *W.dispersa* were separated into three distinct clusters designated as Clades A, B, and C (Fig. 13). Strains NTUPPMCC 22-260, 22-261, 22-263 and 22-266 grouped with the ex-type strain of *W.dispersa* (CBS 297.56) in Clade B. Two other representative strains, CBS 390.61 and CBS 288.67, along with our strains NTUPPMCC 22-262, 22-264, 22-265, and 22-267 to 22-270, formed a separate clade (Clade C), which is sister to the main *W.dispersa* lineage. Additionally, strain CBS 508.75 formed a basal clade (also referred to as Clade A) relative to Clades B and C, complicating the delineation of precise species boundaries. When comparing sequence identity, the representative strain NTUPPMCC 22-269, which clusters in Clade C, showed 97.6% similarity (925/948 bp) in the *tub2* gene to the ex-type strain of *W.dispersa* (CBS 297.56). This strain also formed a strongly supported clade (80% bootstrap support) sister to the clade containing the ex-type strain in single gene phylogeny of *tub2* (See Suppl. material 2: fig. S1). However, no significant genetic divergence was observed between these strains in the ITS and LSU regions (ITS: 433/437 bp, identities 99.1%, including 3 gaps; LSU: 824/830 bp, identities 99.4%, including 4 gaps). As shown in Fig. 15, although PDA cultures exhibited slight variation in colony coloration (NTUPPMCC 22-266 representing Clade B and NTUPPMCC 22-269 representing Clade C; yellow to pale orange versus grayish-white to pale gray-green), other micromorphological features did not show notable differences (See Suppl. material 1: table S4). Additionally, relying solely on culture characteristics is not a reliable method for delineating fungal species (Jeewon and Hyde 2016). Therefore, based on molecular similarity, phylogenetic placement, and morphological consistency, we tentatively identify strains in three clades as *W.dispersa*. The observed genetic variation may be related to their geographical origin or the specific habitats from which these strains were isolated. Consistent with previous studies (Sue et al. 2014), both sexual and asexual stages were observed in culture for *W.dispersa* in the present study. This report represents the first record of *W.dispersa* in Taiwan.

##### ﻿ Westerdykella formosana

Taxon classificationAnimaliaPleosporalesSporormiaceae

K.W. Cheng & H.A. Ariyaw. sp. nov.

18B540A8-218D-54ED-8688-CCB24B8A4436

MB858706

Fig. 16

###### Typification.

TAIWAN • Wanrung Township, Hualien County, 23°42'40.3"N, 121°24'48.2"E, serpentine soil in rice field, 2^nd^ November 2022, K.W Cheng, holotype, NTUPPMH 22-218 (Permanently preserved in a metabolically inactive state), ex-holotype NTUPPMCC 22-255, ex-isotype NTUPPMCC 22-256 to 259.

###### Etymology.

Named after Formosa, the former name of Taiwan, where the type specimen was collected.

###### Description.

***Sexual morph Cleistothecia*** 250–430 µm diam, non-ostiolate, globose, glabrous, mostly superficial, some submerged in PDA, dirty gray when immature, black when mature. ***Peridium*** single-layered, brown, translucent, membranous, angular cells. ***Asci*** subglobose to globose, hyaline when immature, 32-spored, 15.7–21.0 µm × 14.6–18.4 µm (x̄ = 18.3 × 16.3 µm, L/W ratio = 1.1, n = 30). ***Ascospores*** ellipsoidal, smooth, subhyaline to light brown, 1 to 3 guttules, no germ-slits, 3.4–6.4 µm × 1.8–3.2 µm (x̄ = 5.2 × 2.6 µm, L/W ratio = 2.01, n = 50). ***Asexual morph*** undetermined.

###### Culture characteristics.

Colony exhibits rapid growth, reaching 80 mm diam with flat, sparse aerial mycelium, creamy white, surface and margins smooth, pale gray in central region due to the presence of cleistothecia.

###### Notes.

**Figure 16.**
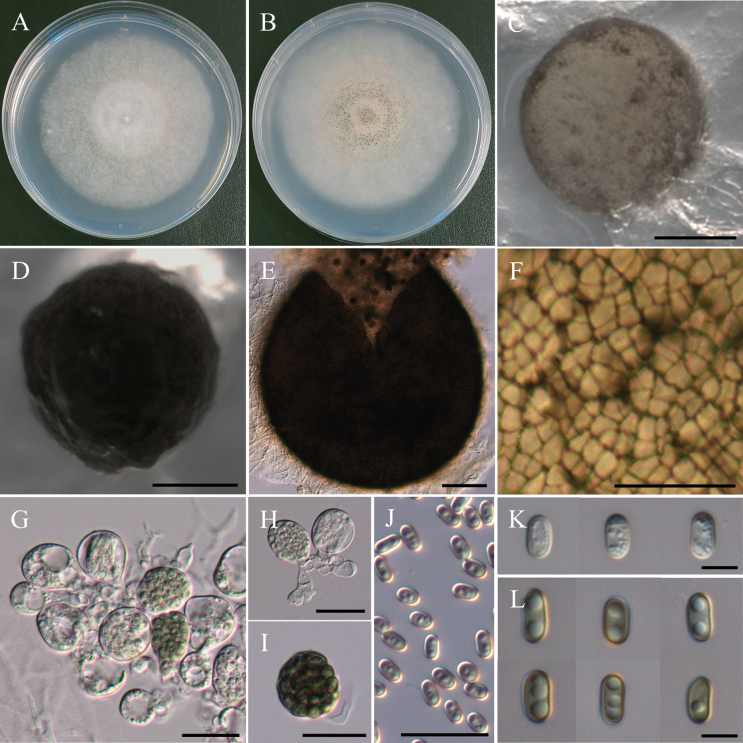
Morphology of *Westerdykellaformosana*NTUPPMCC 22-255. **A, B** 14-days-old colony on PDA; **C** Ascomata; **D** Immature ascoma; **E** Squashed ascomata; **F** Peridium; **G, H** Immature ascus; **I** Ascus; **J, L** Ascospore; **K** Immature ascospore. Scale bars: 100 µm (**C, E**); 200 µm (**D**); 20 µm (**F–J**); 5 µm (**K, L**).

*Westerdykellaformosana* forms a distinct clade in our phylogenetic analysis (Fig. 13 and Suppl. material 2: figs S1, S2). The ex-type strain of *W.formosana* (NTUPPMCC 22-255) exhibits significant genetic divergence from its closest relative, the ex-type strain of *W.aquatica* (JAUCC 1788), with 94.7% identity in the ITS region (392/414 bp, including 1 gap), and from the representative strain PY1 of *W.aquatica* in the *tub2* gene (96.7% identity; 857/886 bp). Morphologically, *W.formosana* produces larger asci but smaller ascospores compared to its phylogenetically closest relative, *W.aquatica* (x̄ = 18.3 × 16.3 µm versus 15.3 × 14.1 µm; x̄ = 5.2 × 2.6 µm versus 6.5 × 2.9 µm). Additionally, *W.formosana* lacks the yellow hue observed in PDA cultures of W.aquatica (Fig. 16; Song et al. 2020). Based on molecular, morphological, and cultural differences, we propose our five strains (NTUPPMCC 22-255 to 22-259) as a novel species, *Westerdykellaformosana*. Further morphological comparisons with other *Westerdykella* species are provided in Suppl. material 1: table S4.

##### ﻿ Westerdykella globosa

Taxon classificationAnimaliaPleosporalesSporormiaceae

T. Ito & A. Nakagiri (1995)

54AEEA1A-954B-5DE4-8663-451136174EE2

MB415330

Fig. 17

###### Description.

***Sexual morph Cleistothecia*** clustered in submerged regions in PDA, 106–185 µm diam, globose to subglobose, dirty gray when immature, dark brown to black when mature. ***Asci*** subglobose to ovoid, hyaline when immature, greenish brown when mature, 32-spored, edges slightly irregular due to crowding of mature ascospores, 26.9–40.2 µm × 20.8–29.8 µm (x̄ = 32.7 × 24.5 µm, L/W ratio = 1.35, n = 30). ***Ascospores*** mostly globose, some subglobose, smooth, yellowish-brown, 1 to 2 guttules, 5.2–6.8 µm × 5.4–6.9 µm (x̄ = 6.1 × 5.9 µm, L/W ratio = 1.03, n = 50). ***Asexual morph*** undetermined.

###### Culture characteristics.

Colony reaching 50 mm diam with slightly diffused edge, predominantly creamy white and partly fluffy, with only the cleistothecia cluster region turning dark brown.

###### Material examined.

TAIWAN • Guanshan Township, Taitung County, 23°02'14.8"N, 121°11'22.6"E, serpentine soil in rice field, 3^rd^ November 2022, K.W. Cheng, living culture NTUPPMCC 22-243 to 247.

###### Notes.

**Figure 17.**
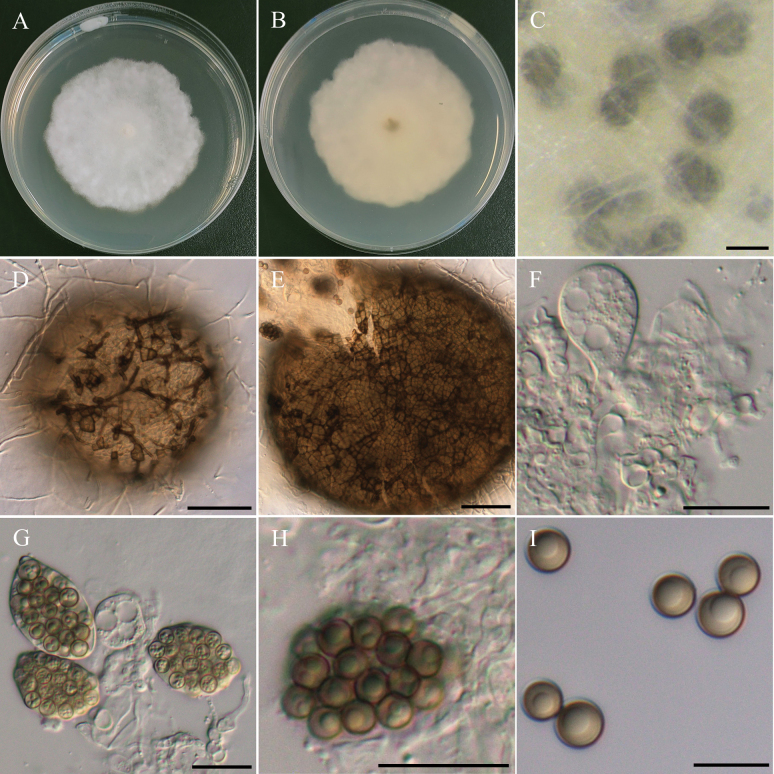
Morphology of *Westerdykellaglobosa*NTUPPMCC 22-246. **A, B** 14-days-old colony on PDA; **C** Ascomata; **D** Immature ascomata; **E** Squashed ascomata; **F, G** Immature asci; **H** Ascus; **I** Ascospores. Scale bars: 0.1 mm (**C**); 50 µm (**D**); 20 µm (**F–H**); 10 µm (**I**).

*Preussiaglobosa* was synonymized under *Westerdykellaglobosa* by Ito (1995). This species has been reported from various environments, including soil from a stream bank and stored wheat grains in India (Rai and Tewari 1963; Kumari et al. 2019), paddy soil in Japan (Ito and Nakagiri 1995), and soil cultivated with *Ganodermalucidum* in China (Zaheer et al. 2024). Our study recovered five strains (NTUPPMCC 22-243–247) that clustered in a strongly supported clade (100%/1.00) with the ex-type strain of *W.globosa* (IFO 32588) (Rai and Tewari 1963), confirming their identity as *W.globosa* (Fig. 13). The strains isolated in the present study share similar morphologies with *W.globosa* in producing globose, brown mature ascospores. Notably, consistent with previous studies, only the sexual stage was observed for strains identified as *W.globosa* in the present study. However, it is worthy to note that the asci of our strains are larger than previously reported (27–40 µm × 21–30 µm versus 20–24 µm × 14–17 µm) (Fig. 17; Ito and Nakagiri 1995). This is the first report of *Westerdykellaglobosa* in Taiwan.

#### ﻿*Eurotiomycetes* O.E. Eriksson & Winka

***Eurotiales* G.W. Martin ex Benny & Kimbrough**

***Trichocomaceae* E. Fischer**

##### ﻿ Talaromyces

Taxon classificationAnimaliaEurotialesTrichocomaceae

C.R. Benjamin

106CF642-4683-50F8-976E-B5FF1CB41DBB

###### Notes.

The genus *Talaromyces* was first established by Benjamin (1955) and used to accommodate sexual stages of some *Penicillium* species. Currently, *Talaromyces* is the largest genus in the family *Trichocomaceae*, which is recorded in over 170 accepted species classified into 8 sections in Mycobank (Accession date: March 10, 2025). *Talaromyces* has a global distribution and has been reported from a wide range of substrates including air, indoor environments, plant materials, food products, dung, but mostly from soils (Hyde et al. 2024; Visagie et al. 2024). Some *Talaromyces* species play a key role as endophytes, helping plants against pathogens and promoting plant growth (Naraghi et al. 2012; Hashem et al. 2023; Nicoletti et al. 2023b). Additionally, while some species can cause diseases in humans, others show activity against human cancer cell lines (Chan et al. 2016; Zhai et al. 2016; Nicoletti et al. 2023a). In genus *Talaromyces*, specifically within the section Talaromyces, both asexual and sexual morphs have been recorded in some species, exhibiting considerable morphological diversity. In the asexual morph, most species possess bi-verticillate conidiophores, although some exhibit both bi-verticillate and mono-verticillate conidiophores (Nguyen et al. 2023).

##### ﻿ Talaromyces adpressus

Taxon classificationAnimaliaEurotialesTrichocomaceae

A.J. Chen, J.C. Frisvad & R.A. Samson (2016)

51C5D6B6-70E2-5C1F-B954-2F084068DDC1

MB817397

Fig. 18

###### Description.

***Sexual morph*** undetermined. ***Asexual morph Conidiophores*** arose from aerial hyphae, or roping hyphal aggregations, hyaline, straight, bi-verticillate. ***Metulae*** 9.6–15.5 µm × 2.4–3.0 µm. ***Phialides*** 3–5, flask-shaped, 8.5–10.8 µm × 2.1–2.6 µm. ***Conidia*** globose to subglobose, hyaline, few pale green, 2.2–2.5 µm × 1.6–2.1 µm (x̄ = 2.3 × 1.9 µm, L/W ratio = 1.20, n = 25).

###### Medium dependent growth in 7/14 days at 25 °C (mm).

• CYA 23–25/33–38; CYAS No growth; MEA 55–58/80–85; OA 43–46/85–90; PDA 38–42/60–65; YESA 43–45/62–65.

###### Temperature dependent growth in 7 days (mm).

• CYA/MEA 20 °C 28–30/30–33; 30 °C 30–33/51–56; 37 °C 16–18/16–19.

###### Culture characteristics.

CYA, 25 °C, 14 days, sulcate, margin entire and white to buff, floccose, sporulation sparse, pale gray to grayish-green, soluble pigments absent, exudates clear droplets; reverse in coffee brown to caramel. MEA, 25 °C, 14 days, margin slightly irregular and beige, floccose to funiculose, sporulation dense, grayish-green, soluble pigments absent, exudates clear droplets; reverse in light yellowish brown. OA, 25 °C, 14 days, margin entire and pale gray, floccose to funiculose, sporulation dense, dark green, soluble pigments absent, exudates clear droplets; reverse in buff. PDA, 25 °C, 14 days, margin irregular and whitish, floccose to funiculose, sporulation dense, olive green, soluble pigments absent, exudates clear droplets; reverse in cream-buff. YESA, 25 °C, 14 days, margin slightly irregular, floccose, sporulation moderately dense, warm beige, soluble pigments and exudates absent; reverse in light gold to metallic gold.

###### Material examined.

TAIWAN • Guanshan Township, Taitung County, 23°02'12.8"N, 121°11'22.0"E, serpentine soil in rice field, 2^nd^ November 2022, K.W Cheng, living culture NTUPPMCC 22-271.

###### Notes.

**Figure 18.**
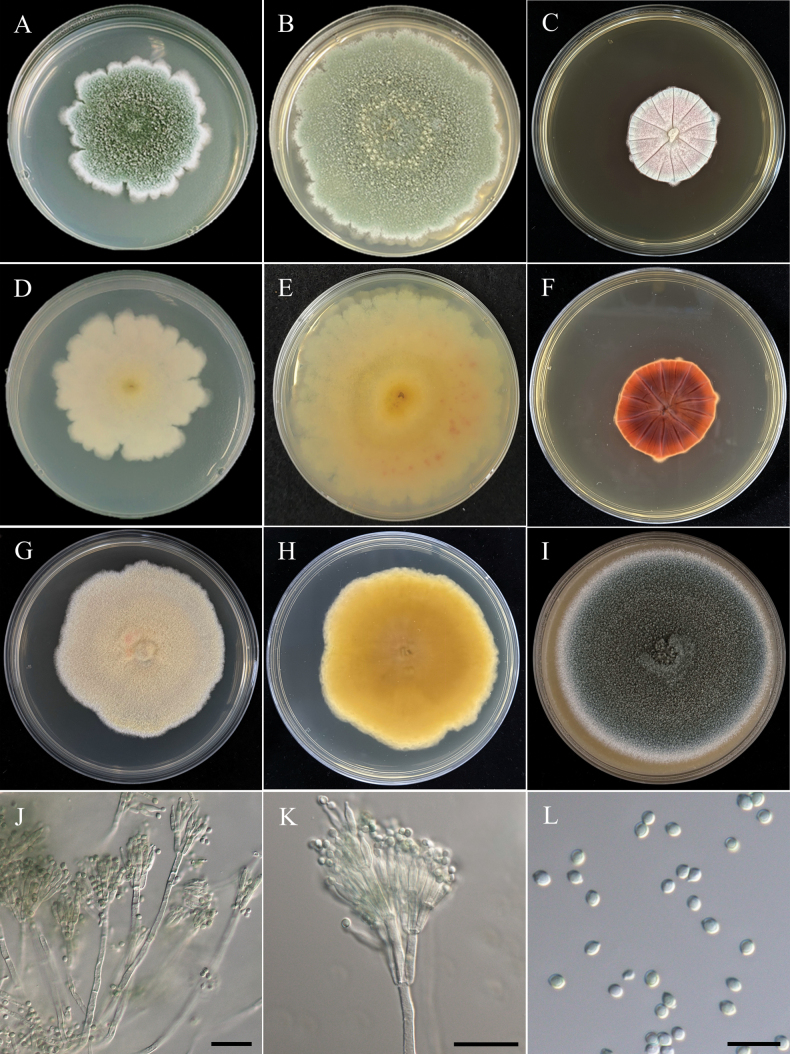
Morphology of *Talaromycesadpressus*NTUPPMCC 22-271. **A, D** 14-days-old colony on PDA; **B, E** 14-days-old colony on MEA; **C, F** 14-days-old colony on CYA; **G, H** 14-days-old colony on YESA; **I** 14-days-old colony on OA; **J, K** Conidiophores, phialides, and conidiogenous cells; **L** Conidia. Scale bars: 20 µm (**J, K**); 10 µm (**L**).

**Figure 19.**
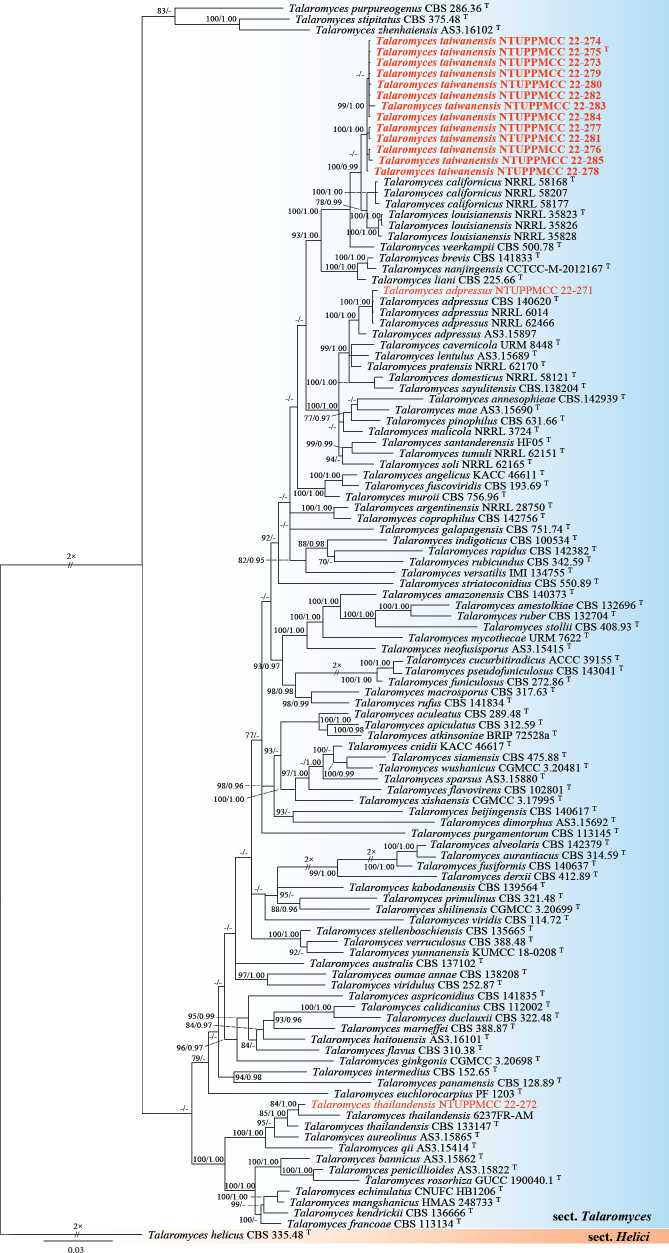
Maximum likelihood (ML) phylogenetic tree based on a concatenated dataset of ITS, *cmd*A, *rpb2*, and *tub2*. Species representing section Talaromyces including 113 strains representing 98 taxa were included in the concatenated dataset, with 2127 characters (ITS 536 bp, *cmd*A 336 bp, *rpb2* 843 bp, and *tub2* 412 bp) including alignment gaps. The tree was rooted with *Talaromyceshelicus* CBS 335.48 (Section Helici). MLB ≥ 70% and BPPs ≥ 0.95 were shown at each node; values lower than these thresholds are indicated by a hyphen (–). The scale bar indicates the number of estimated substitutions per site. The strains introduced in this study are in red and novel species are in bold. The ex-type strains are marked with ^T^.

In the present study, our strain (NTUPPMCC 22-271) clustered within the clade containing ex-type strain, along with other representative strains of *T.adpressus* with strong statistical support (100%/1.00) (Fig. 19). *T.adpressus*NTUPPMCC 22-271 exhibited similar asexual morph to the ex-type strain of *T.adpressus* (CBS 140620), with bi-verticillate conidiophores, phialides 3–5, and subglobose conidia (Fig. 18); however, our strain (NTUPPMCC 22-271) did not grow on CYAS (Chen et al. 2016). *T.adpressus* has been reported from a wide range of substrates including sea sand, indoor air, peanut, *Heteroderazeae* cysts, *Oryzacoarctata* as endophytic fungi (Chen et al. 2016; Peterson and Jurjevic 2019; Airin et al. 2023; Lee et al. 2023; Mo et al. 2024). However, this study represents the first discovery of *T.adpressus* in Taiwan.

##### ﻿ Talaromyces taiwanensis

Taxon classificationAnimaliaEurotialesTrichocomaceae

K.W. Cheng & H.A. Ariyaw. sp. nov.

36206A46-EE1B-5423-A3D6-251729ECA32B

MB858707

Fig. 20

###### Typification.

TAIWAN • Wanrung Township, Hualien County, 23°42'40.3"N, 121°24'48.2"E, serpentine soil in rice field, 2^nd^ November 2022, K.W Cheng, holotype, NTUPPMH 22-219 (Permanently preserved in a metabolically inactive state), ex-holotype NTUPPMCC 22-275, ex-isotype NTUPPMCC 22-273 to 274, 276 to 285.

###### Etymology.

Named after Taiwan, the country where the type specimen was collected.

###### Description.

***Sexual morph*** undetermined. ***Asexual morph Conidiophores*** arose from aerial hyphae, or roping hyphal aggregations, hyaline, smooth but some slightly rough, straight, most mono-verticillate or bi-verticillate, occasionally formed subterminal side branches of mono-verticillate, 5–130 µm. ***Metulae*** 10.5–16.5 µm × 2.8–3.2 µm. ***Phialides*** most 3–5, flask-shaped, 8.2–15.6 µm × 2.0–2.8 µm, rarely mono-phialides up to 18.2 µm × 3.5 µm. ***Conidia*** globose to subglobose, few pyriform, rough surfaces and walls, hyaline in immature, pale green to green in mature, 3.3–4.6 µm × 2.9–4.0 µm (x̄ = 3.8 × 3.5 µm, L/W ratio = 1.1, n = 50).

###### Medium dependent growth in 7/14 days at 25 °C (mm).

• CYA 13–15/30–33; CYAS No growth; MEA 58–60/80–85; OA 48–50/88–90; PDA 52–57/72–75; YESA 37–40/65–72.

###### Temperature dependent growth in 7 days (mm).

• CYA/MEA 20 °C 30–32/38–40; 30 °C 19–21/55–58; 37 °C 15–16/25–28.

###### Culture characteristics.

CYA, 25 °C, 14 days, obvious sulcate, margin entire, floccose, sporulation none, pale gray to beige at center, soluble pigments absent, exudates clear small droplets; reverse in yellowish brown and caramel at center. MEA, 25 °C, 14 days, margin slightly irregular and beige, floccose to funiculose, sporulation dense, grayish-green, soluble pigments absent, exudates clear droplets; reverse in cream-buff. OA, 25 °C, 14 days, margin entire and beige, floccose to funiculose, sporulation dense, olive green, soluble pigments absent, exudates clear small droplets; reverse in buff. PDA, 25 °C, 14 days, margin entire and whitish, floccose to funiculose, sporulation dense, green, soluble pigments absent, exudates clear droplets; reverse in cream-buff. YESA, 25 °C, 14 days, margin slightly irregular, floccose, sporulation moderately dense, warm beige, soluble pigments absent, exudates clear small droplets; reverse in warm beige to copper brown.

###### Notes.

**Figure 20.**
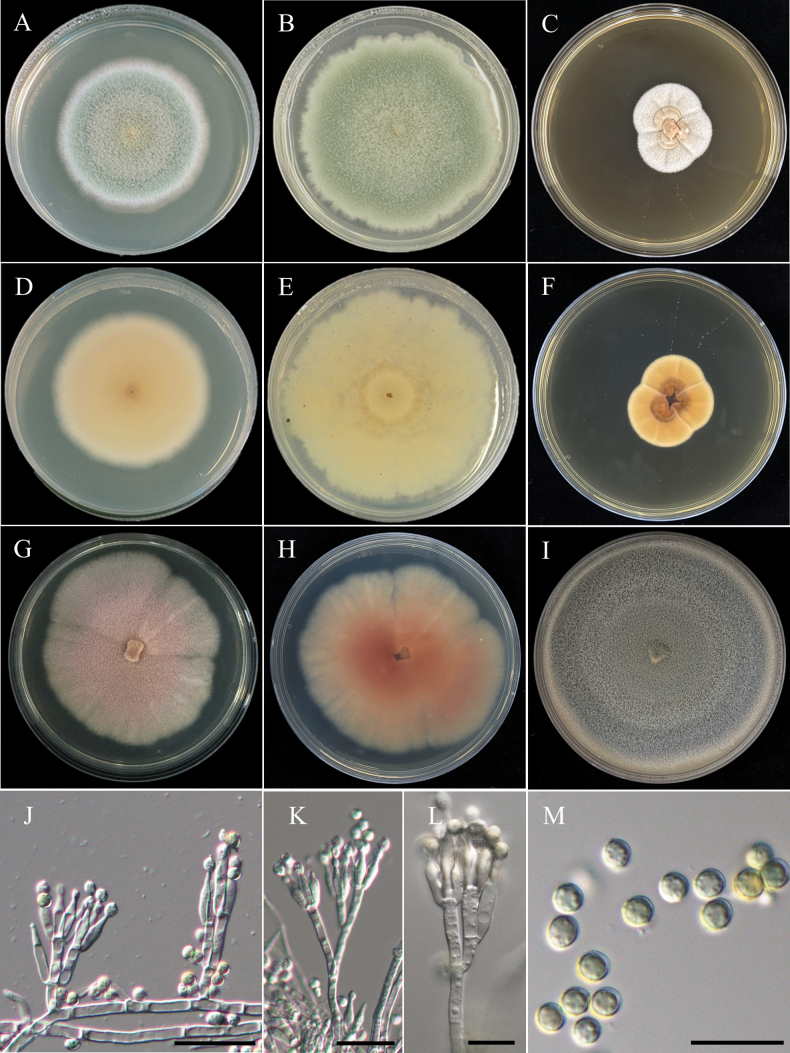
Morphology of *Talaromycestaiwanensis*NTUPPMCC 22-275. **A, D** 14-days-old colony on PDA; **B, E** 14-days-old colony on MEA; **C, F** 14-days-old colony on CYA; **G, H** 14-days-old colony on YESA; **I** 14-days-old colony on OA; **J–L** Conidiophores, phialides, and conidiogenous cells; **M** Conidia. Scale bars: 20 µm (**J, K**); 10 µm (**L, M**).

*Talaromycestaiwanensis* forms a strongly supported clade (100%/0.99) with its sister species *T.californicus* and *T.louisianensis* in the multi-locus phylogeny (Fig. 19). The ex-type strain of *T.taiwanensis* (NTUPPMCC 22-275) shows over 98.5% sequence similarity across the ITS, *rpb2*, and *tub2* regions when compared to the ex-type strains of *T.californicus* (NRRL 58168) and *T.louisianensis* (NRRL 35823). However, a small genetic variation is observed in the *cmd*A region, where *T.taiwanensis* exhibits 97.6% identity (325/333 bp, including 1 gap) to these sister species. Morphologically, *T.taiwanensis* displays conidial structure similar to *T.californicus* characterized by mono-verticillate and single phialides, a rare phenotypic feature in the T. sect. of Talaromyces (Fig. 20; Peterson and Jurjevic 2019). However, *T.taiwanensis*NTUPPMCC 22-275 formed smaller conidia to its phylogenetically closely related species *T.californicus* and *T.louisianensis* (x̄ = 3.0–5.0 µm × 3.0–4.0 µm versus 4.0–6.0 µm × 4.0–7.0 µm and versus 3.5–5.0 µm × 3.5–5.0 µm). Furthermore, *T.taiwanensis* demonstrates significantly slower growth (13–15 mm) compared to *T.californicus* (25–40 mm) and *T.louisianensis* (35–39 mm) and lacks sporulation on CYA (Peterson and Jurjevic 2019). Furthermore, these two species were isolated from air in USA and our samples were from serpentine soil in Taiwan (Peterson and Jurjevic 2019).

##### ﻿ Talaromyces thailandensis

Taxon classificationAnimaliaEurotialesTrichocomaceae

L. Manoch, T. Dethoup & N. Yilmaz (2013)

BF3CBA26-6412-5AA1-B31E-AF423F9F16A9

MB801737

Fig. 21

###### Description.

***Sexual morph Ascomata*** solitary or clustered, superficial, globose to subglobose, yellow hyphae covered, 220–470 µm diam. ***Asci*** subglobose to ovoid, hyaline, 8.6–12.7 µm × 7.9–10.1 µm (x̄ = 10.9 × 9.6 µm, L/W ratio = 1.14, n = 15). ***Ascospores*** ellipsoidal, spiny, thick walled, 4.2–5.0 µm × 2.8–3.5 µm (x̄ = 4.6 × 3.2 µm, L/W ratio = 1.47, n = 25). ***Asexual morph Conidiophores*** straight, bi-verticillate, smooth walled and long stipes, up to 300 µm. ***Metulae*** 9.1–14.0 µm × 2.5–2.8 µm. ***Phialides*** 3–6, flask-shaped, 8.1–12.5 µm × 2.2–2.5 µm. ***Conidia*** globose to subglobose, smooth walled, hyaline to pale brownish green, 2.0–2.8 µm × 1.6–2.4 µm (x̄ = 2.4 × 2.0 µm, L/W ratio = 1.20, n = 25).

###### Medium dependent growth in 7/14 days at 25 °C (mm).

• CYA 35–38/58–61; CYAS 15–17/31–33; MEA 40–42/70–75; OA 39–42/85–90; PDA 38–41/70–75; YESA 30–33/43–48.

###### Temperature dependent growth in 7 days (mm).

• CYA/MEA 20 °C 32–34/31–33; 30 °C 40–44/37–42; 37 °C No growth/No growth.

###### Culture characteristics.

CYA, 25 °C, 14 days, margin entire and light yellow, floccose, sporulation dense, yellowish orange, soluble pigments absent, exudates clear droplets; reverse in orange and copper brown at center. MEA, 25 °C, 14 days, margin entire and whitish, floccose, sporulation dense, grayish-green, soluble pigments absent, exudates clear droplets; reverse in cream-buff. OA, 25 °C, 14 days, margin entire and golden yellow, floccose to funiculose, sporulation dense, orange to grayish-green and wine red at center, soluble pigments absent, exudates clear droplets; reverse in buff. PDA, 25 °C, 14 days, margin entire and light yellow, floccose, sporulation dense, yellowish orange to grayish-green, soluble pigments absent, exudates clear droplets; reverse in cream-buff. YESA, 25 °C, 14 days, slightly sulcate, margin irregular, floccose, sporulation moderately dense, yellowish orange, soluble pigments and exudates absent; reverse in orange-brown.

###### Material examined.

TAIWAN • Guanshan Township, Taitung County, 23°02'17.6"N, 121°11'26.3"E, serpentine soil in rice field, 3^rd^ November 2022, K.W. Cheng, living culture NTUPPMCC 22-272.

###### Notes.

**Figure 21.**
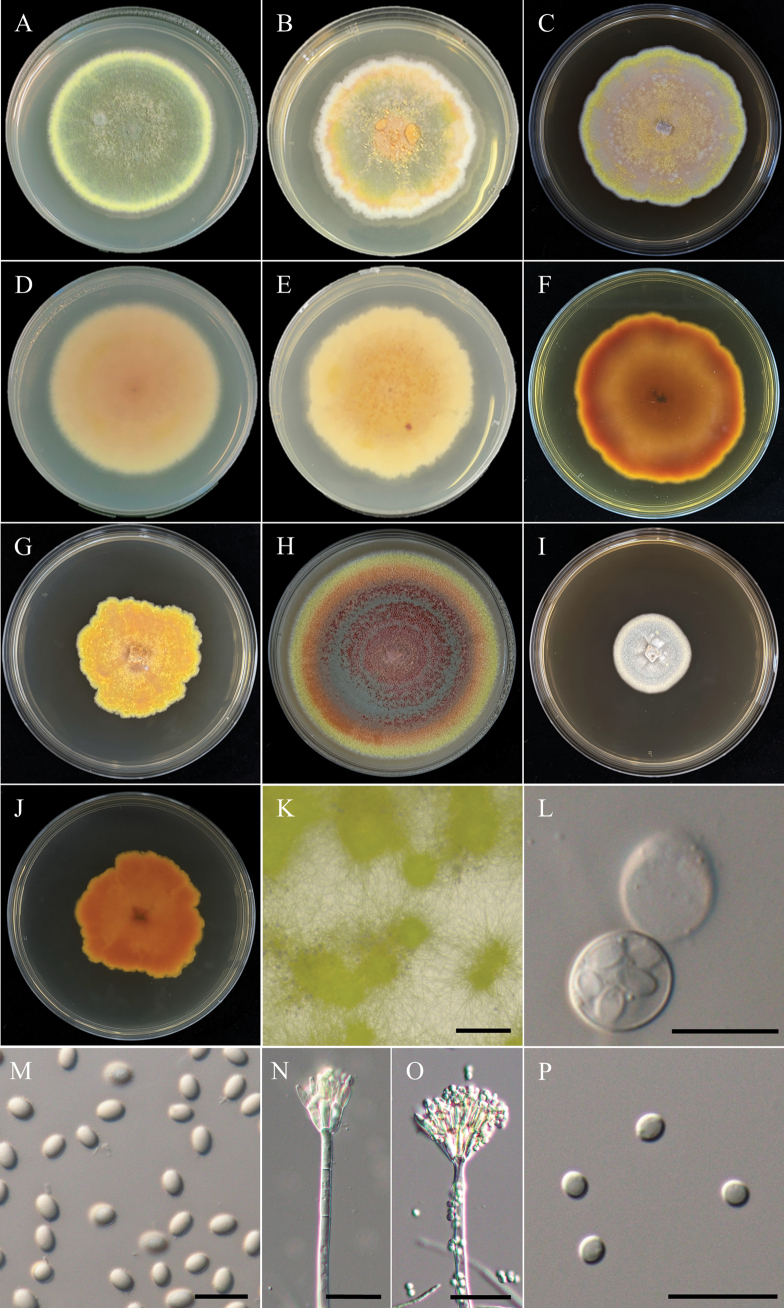
Morphology of *Talaromycesthailandensis*NTUPPMCC 22-272. **A, D** 14-days-old colony on PDA; **B, E** 14-days-old colony on MEA; **C, F** 14-days-old colony on CYA; **G, J** 14-days-old colony on YESA; **H** 14-days-old colony on OA; **I** 14-days-old colony on CYAS; **K** Ascomata and conidiophores; **L** Asci; **M** Ascospores; **N, O** Conidiophores, phialides, and conidiogenous cells; **P** Conidia. Scale bars: 0.5 mm (K); 10 µm (**L, N–P**); 5 µm (**M**).

In the present study, our strain (NTUPPMCC 22-272) clustered within the clade containing ex-type strain, along with other representative strains of *T.thailandensis* with strong statistical support (85%/1.00) (Fig. 19). *T.thailandensis* (NTUPPMCC 22-272) displayed morphological features similar to the ex-type strain (CBS 133147) as reported by Manoch et al. (2013) and Yilmaz et al. (2014). Typically, these species exhibit ellipsoidal, spiny, thick-walled ascospores (sexual stage), bi-verticillate conidiophores with 3–6 phialides, and subglobose conidia (asexual stage), along with an absence of growth at 37 °C. However, our strain (NTUPPMCC 22-272) differs by presenting a distinctive red hue on OA (Fig. 21). *T.thailandensis* has been reported previously in Thailand as soil-derived fungus (Manoch et al. 2013; Ningsih et al. 2024). However, this study represents the first discovery of *T.thailandensis* in Taiwan.

#### ﻿*Sordariomycetes* O.E. Eriksson & Winka

***Glomerellales* Chadef. ex Réblová, W. Gams & K.A. Seifert**

***Reticulascaceae* Réblová & W. Gams**

##### ﻿ Cylindrotrichum

Taxon classificationAnimaliaGlomerellalesReticulascaceae

Bonorden

4FD23AB3-42DB-5518-9F67-CB6A8E949474

###### Notes.

*Cylindrotrichumoligospermum* was originally used by (Corda) Bonord. to establish the genus *Cylindrotrichum*, while *Reticulascus* was introduced as a new genus with *Reticulascusclavatus* as the type species, and *Cylindrotrichumclavatum* as its asexual morph. Later, *C.oligospermum*, including *C.hennebertii*, was recombined and treated as a synonym of *R.tulasneorum* (Réblová et al. 2011). However, Réblová et al. (2016) recommended the continued use of *Cylindrotrichum* over *Reticulascus* due to its wider usage and greater number of associated names. Additionally, *Blastophorumaquaticum* was synonymized with *Cylindrotrichumaquaticum* by Luo et al. (2019). The asexual morph of *Cylindrotrichum* is characterized by the absence of setae. Conidiophores are cylindrical, straight to flexuous, solitary or in clusters, mononematous, and macronematous. Conidiogenous cells are usually monophialidic, rarely polyphialidic, with a hyaline to subhyaline collarette. Conidia are hyaline, cylindrical, slightly tapering with an obtuse apex, septate, guttulate, and smooth-walled (Luo et al. 2019; Crous et al. 2022). The sexual morph lacks stroma and produces superficial ascomata that are subglobose to conical, brown, and occur solitarily or in gregarious groups. The ostiolum is periphysate. Asci are eight-spored, cylindrical-clavate, unitunicate, and short-stipitate. Ascospores are hyaline, ellipsoidal to fusiform, and septate (Réblová et al. 2011; Luo et al. 2019). Cylindrotrichum (Reticulascus) strains has been reported from Australia, China, Czech Republic, France, Germany, and Netherlands as a saprobe on dead wood (Réblová et al. 2011; Luo et al. 2019; Crous et al. 2022).

##### ﻿ Cylindrotrichum formosanum

Taxon classificationAnimaliaGlomerellalesReticulascaceae

K.W. Cheng & H.A. Ariyaw. sp. nov.

21C9070A-9051-5969-BCB5-E2A6835EC301

MB858714

Fig. 22

###### Typification.

TAIWAN • Guanshan Township, Taitung County, 23°02'12.8"N, 121°11'22.0"E, serpentine soil in rice field, 2^nd^ November 2022, K.W Cheng, holotype, NTUPPMH 22-220 (Permanently preserved in a metabolically inactive state), ex-holotype NTUPPMCC 22-287, ex-isotype NTUPPMCC 22-286.

###### Etymology.

Named after Formosa, the former name of Taiwan, where the type specimen was collected.

###### Description.

***Sexual morph*** undetermined. ***Asexual morph*** Conidia produced on carnation leaves and WA. ***Conidiophores*** solitary, subcylindrical, light brown, unbranched, straight to slightly flexuous, and thick-walled. ***Conidiogenous cells*** subcylindrical, light brown to light gray, terminal or intercalary, with flared collarettes. ***Conidia*** solitary or aggregated in clusters, subcylindrical to slightly curved, with an obtuse apex, guttules, hyaline, smooth-walled, and 0 to 3-septate, 13.4–19.9 µm × 3.8–6.1 µm (x̄ = 16.3 × 4.6 µm, L/W ratio = 3.6, n = 50). ***Chlamydospores*** rusty copper-brown, circular to slightly ellipsoidal, clustered 1–3 on PDA and WA. Single chlamydospores 6.1–8.9 µm × 5.9–8.4 µm (x̄ = 7.3 × 7.2 µm, L/W ratio = 1.0, n = 50).

###### Culture characteristics.

Colony reaching 60 mm diam with flat, spreading, gray margin and dark gray blue in the center, reverse similar.

###### Notes.

**Figure 22.**
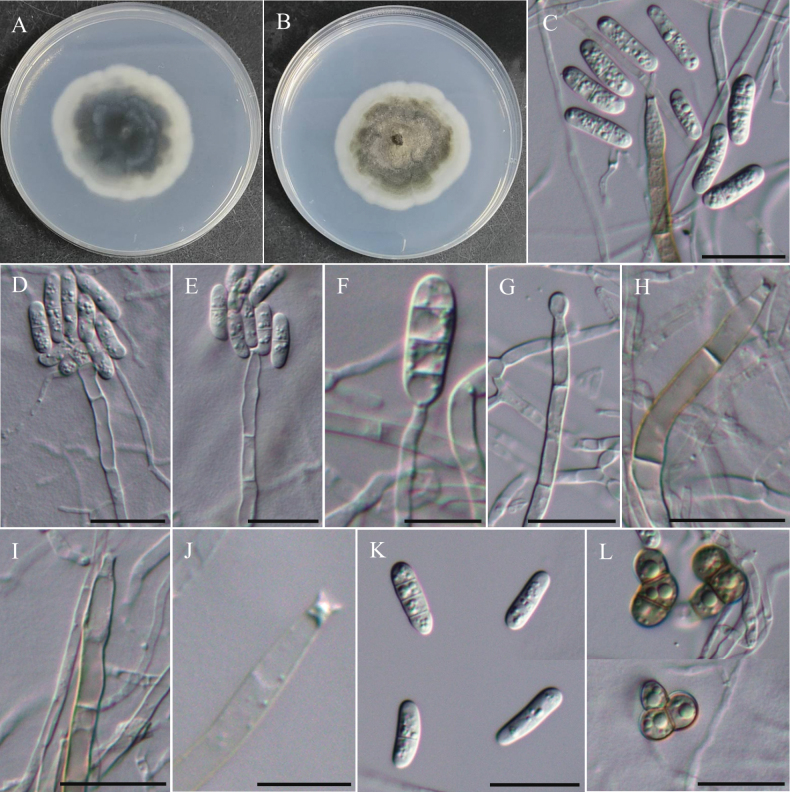
Morphology of *Cylindrotrichumformosanum*NTUPPMCC 22-287. **A, B** 14-days-old colony on PDA; **C–G** Conidiophores and conidiogenous cells giving rise to conidia; **H**, **I** Conidiophore; **J** Conidiogenous cells; **K** Conidia; **L** Chlamydospores. Scale bars: 20 µm (**C–E, G–I, K, L**); 10 µm (**F**, **J**).

**Figure 23.**
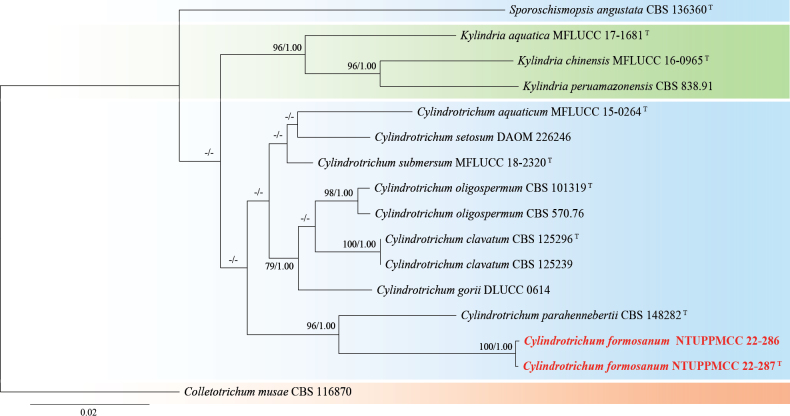
Maximum likelihood (ML) phylogenetic tree based on a concatenated dataset of ITS, LSU, and SSU. In total, 16 strains representing 13 taxa were included in the concatenated dataset, with 2055 characters (ITS 475 bp, LSU 848 bp, and SSU 732 bp) including alignment gaps. The tree was rooted with *Colletotrichummusae* CBS 116870. MLB ≥ 70% and BPPs ≥ 0.95 were shown at each node; values lower than these thresholds are indicated by a hyphen (–). The scale bar indicates the number of estimated substitutions per site. The strains introduced in this study are in red and novel species are in bold. The ex-type strains are marked with ^T^.

*Cylindrotrichumformosanum*NTUPPMCC 22-287 is typical of *Cylindrotrichum* in having straight to flexuous, brown, subcylindrical conidiophores, terminal or intercalary conidiogenous cells with flared collarettes, and subcylindrical, smooth, hyaline conidia (Fig. 22; Réblová et al. 2011; Crous et al. 2022). However, our strains (NTUPPMCC 22-286 and NTUPPMCC 22-287) can be easily differentiated from its closely related species based on both phylogeny and morphology (Fig. 23). *C.formosanum* present a distinct clade with a strongly support (96%/1.00) as a sister group to *C.parahennebertii* in our multi-locus phylogenetic analysis. Moreover, the ex-type strain of *C.formosanum* (NTUPPMCC 22-287) exhibits significant genetic divergence from the ex-type strain of *C.parahennebertii* (CBS 148282) , with 84.2% identity in the ITS region (378/449 bp, including 41 gaps) and 97.3% identity in the LSU region (783/805 bp, including 2 gaps). Morphologically, *C.formosanum* produces larger conidia than *C.parahennebertii* (x̄ = 16.3 × 4.6 µm versus 13.5 × 3.8 µm). Additionally, conidia of *C.formosanum* are 0- to 3-septate, whereas *C.parahennebertii* consistently produces distinctly 3-septate conidia (Fig. 22; Crous et al. 2022). Beyond commonly observed morphological features, this study also reports a novel characteristic for *Cylindrotrichum* species: the presence of rusty copper-brown, circular to slightly ellipsoidal chlamydospores observed in culture (Fig. 22L). Based on these molecular and morphological differences, we propose our two strains (NTUPPMCC 22-286 and NTUPPMCC 22-287) as a novel species, *Cylindrotrichumformosanum*.

#### ﻿*Hypocreales* Lindau

***Sarocladiaceae* L. Lombard**

##### ﻿ Parasarocladium

Taxon classificationAnimaliaHypocrealesSarocladiaceae

R.C. Summerbell, J.A. Scott, J. Guarro & P.W. Crous

3FC3B98B-71A8-5ACC-B149-B634F0371C4D

###### Notes.

The genus *Parasarocladium* was first introduced by Summerbell et al. (2018) to accommodate three soil borne, acremonium-like species, and is typified by *Pa.radiatum*, which was isolated from soil in India. Currently, 15 species epithets are recognized for *Parasarocladium* in Mycobank (Accession date: March 10, 2025). Conidiophores of *Parasarocladium* species are solitary or aggregated, arising from aerial or substratal mycelium, erect, aseptate or septate, smooth, hyaline. Conidiogenous cells are phialidic, hyaline, smooth, lateral or terminal, straight or irregularly curved, monophialides or polyphialides. The conidia are hyaline, smooth, ellipsoidal to ovate, or bacilliform to fusiform, aseptate, sometimes slightly curved, forming slimy heads on the phialides (Summerbell et al. 2018; Lee et al. 2025). *Parasarocladium* has a global distribution and has been isolated from soil and plants as a soil borne fungus, plant pathogen, and endophyte. (Summerbell et al. 2018; Hou et al. 2023).

##### ﻿ Parasarocladium formosum

Taxon classificationAnimaliaHypocrealesSarocladiaceae

K.W. Cheng & H.A. Ariyaw. sp. nov.

E5999C2B-8014-521E-BE1F-4DA3293C22AF

MB858711

Fig. 24

###### Typification.

TAIWAN • Guanshan Township, Taitung County,23°02'17.6"N, 121°11'26.3"E, serpentine soil in rice field, 3^rd^ November 2022, K.W. Cheng, holotype, NTUPPMH 22-221 (Permanently preserved in a metabolically inactive state), ex-holotype NTUPPMCC 22-288.

###### Etymology.

Named after Formosa, the former name of Taiwan, where the type specimen was collected.

###### Description.

***Sexual morph*** undetermined. ***Asexual morph*** Conidia were observed on WA. ***Conidiophores*** mostly solitary, phialidic, straight but some curved, smooth, hyaline, arising directly from aerial or substratal hyphe, unbranched, mono-phialides or adelophialides predominant, 7–18 × 2–3 μm. ***Conidia*** ellipsoidal, sometimes fusoid, hyaline, aseptate, smooth-walled, 1-celled, several tiny guttules, arranged in slimy heads, 3.7–7.4 µm × 2.0–3.9 µm (x̄ = 4.9 × 2.6 µm, L/W ratio = 1.94, n = 50).

###### Culture characteristics.

Colony exhibits slow growth, reaching 35 mm diam with creamy white, radially folded, slightly rugose at center region, smooth margin, reverse pale yellow.

###### Notes.

**Figure 24.**
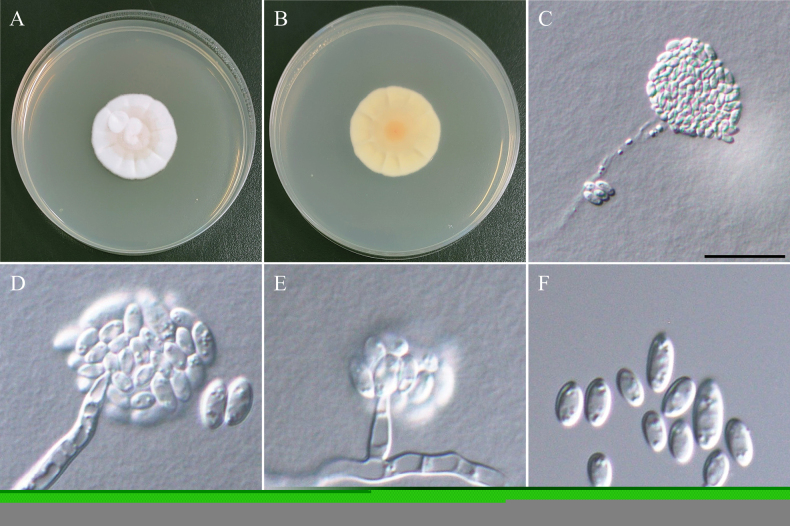
Morphology of *Parasarocladiumformosum*NTUPPMCC 22-288. **A, B** 14-days-old colony on PDA; **C–E** Conidiophores, phialides, and conidiogenous cells; **F** Conidia. Scale bars: 20 µm (**C**); 10 µm (**D–F**).

**Figure 25.**
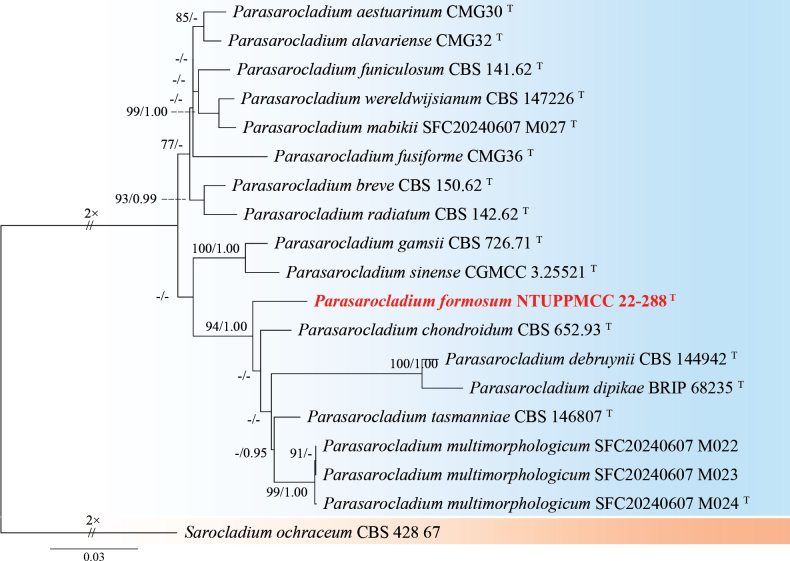
Maximum likelihood (ML) phylogenetic tree based on a concatenated dataset of ITS, LSU, and *tef-1*. In total, 19 strains representing 17 taxa were included in the concatenated dataset, with 2159 characters (ITS 505 bp, LSU 809 bp, and *tef-1* 845 bp) including alignment gaps. The tree was rooted with *Sarocladiumochraceum* CBS 428.67. MLB ≥ 70% and BPPs ≥ 0.95 were shown at each node; values lower than these thresholds are indicated by a hyphen (–). The scale bar indicates the number of estimated substitutions per site. The strains introduced in this study are in red and novel species are in bold. The ex-type strains are marked with ^T^.

*Parasarocladiumformosum* forms a distinct clade with strong support (94%/1.00), as a sister taxon to *Pa.chondroidum* in our multi-locus phylogenetic analysis (Fig. 25). Moreover, the ex-type strain of *Pa.formosum* (NTUPPMCC 22-288) shows significant genetic divergence from its closest relative, the ex-type strain of *Pa.chondroidum* (CBS 652.93), with 96.7% identity in the ITS region (438/453 bp, including 4 gaps) and 93.1% identity in the *tef-1* gene (752/808 bp). It is worth noting that *Pa.chondroidum* has been reported as an endophyte in *Gramineae*, whereas *Pa.formosum*NTUPPMCC 22-288, although isolated from a paddy field, was recovered from soil and has not been identified as an endophyte (Hou et al. 2023). Moreover, the conidia of our new species are slightly larger and straighter compared to the original description of *Pa.chondroidum* CBS 652.93, which exhibited relatively smaller and curved conidia. (3.7–7.4 µm × 2.0–3.9 µm versus 3.4–8 µm × 1.2–2.5 µm) (Fig. 24; Hou et al. 2023). Based on these molecular and morphological differences, we propose our strain (NTUPPMCC 22-288) as a novel species, *Parasarocladiumformosum*.

##### ﻿ Sarocladium

Taxon classificationAnimaliaHypocrealesSarocladiaceae

W. Gams & D. Hawksworth

ACBEFFC5-7D9F-5A05-A4DF-822D4E11A664

###### Notes.

The genus *Sarocladium* was introduced by Gams and Hawksworth (1975) to accommodate two rice (*Oryzasativa*) pathogens, *S.oryzae* and *S.attenuatum*, with the former as type species. Currently, 38 species epithets are recognized for *Sarocladium* in Mycobank (Accession date: March 10, 2025). Conidiophores of *Sarocladium* are mononematous, hyaline, arising from aerial mycelium, submerged hyphae or hyphal ropes. They are straight or slightly curved, mono-, poly- or adelophialidic with smooth-walls. Conidia are hyaline to subhyaline, smooth-walled, and highly variable in shape—ranging from cylindrical, bacilliform, oblong, ovoid, fusoid, and limoniform to subglobose or irregular. They are typically produced in slimy heads or dry chains. Additionally, recent studies have reported the occasional presence of crystals and chlamydospores in some species (Giraldo et al. 2015; Hou et al. 2023). *Sarocladium* has a global distribution (Ou et al. 2020). Species in *Sarocladium* have been reported as plant pathogens of rice and apple fruit and some species reported as opportunistic human pathogens, and saprophytic fungi in soil or plant debris. Furthermore, recent studies have reported them as endophytes in tropical grasses, coastal grass, and crops (Yeh and Kirschner 2014; Giraldo et al. 2015; Gonzáles-Teuber et al. 2017; Hou et al. 2019; Anjos et al. 2020; Ou et al. 2020).

##### ﻿ Sarocladium formosanum

Taxon classificationAnimaliaHypocrealesSarocladiaceae

K.W. Cheng & H.A. Ariyaw. sp. nov.

AC19729E-0137-562D-BB7C-A8BF51D12454

MB858715

Fig. 26

###### Typification.

TAIWAN • Guanshan Township, Taitung County, 23°02'17.6"N, 121°11'26.3"E, serpentine soil in rice field, 3^rd^ November 2022, K.W. Cheng, holotype, NTUPPMH 22-222 (Permanently preserved in a metabolically inactive state), ex-holotype NTUPPMCC 22-289.

###### Etymology.

Named after Formosa, the former name of Taiwan, where the type specimen was collected.

###### Description.

***Sexual morph*** undetermined. ***Asexual morph*** Conidia were observed on WA. ***Conidiophores*** solitary, hyaline, straight to slightly flexuous, smooth-walled, arising from hyphal ropes or vegetative hyphae. ***Phialides*** subulate, hyaline, wide at the base, with, 13–30 µm long. ***Adelophialides*** and ***schizophialides*** not observed. ***Conidia*** unicellular, cylindrical with rounded ends, hyaline, smooth-walled, 1-celled, few with inconspicuous 1 to 2 guttules on the end(s), sometimes aggregated in clusters forming a slimy head, 3.5–5.3 µm × 1.1–2.1 µm (x̄ = 4.4 × 1.5 µm, L/W ratio = 3.08, n = 50).

###### Culture characteristics.

Colony exhibit slow growth, reaching 35 mm diam with flat, pale orange, wrinkled in the center, slimy, and smooth margin. The reverse side of the colony displayed similar characteristics.

###### Notes.

**Figure 26.**
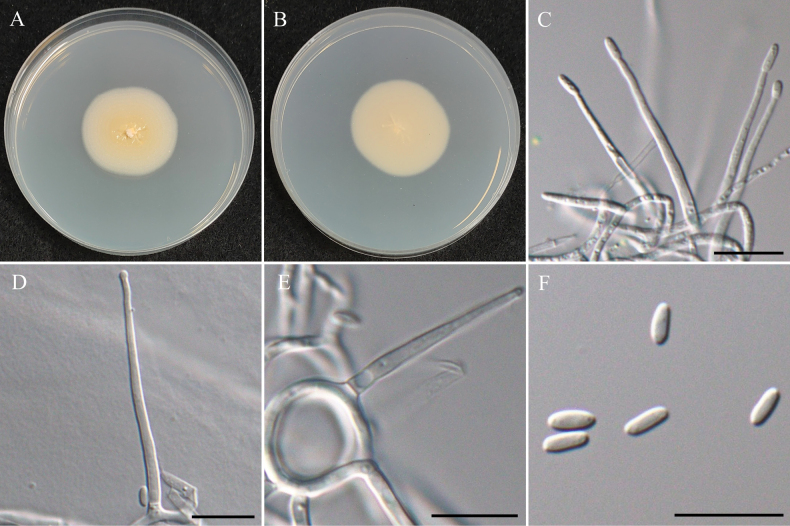
Morphology of *Sarocladiumformosanum*NTUPPMCC 22-289. **A, B** 14-days-old colony on PDA; **C–E** Conidiophores and phialides; **F** Conidia. Scale bars: 10 µm (**C–F**).

**Figure 27.**
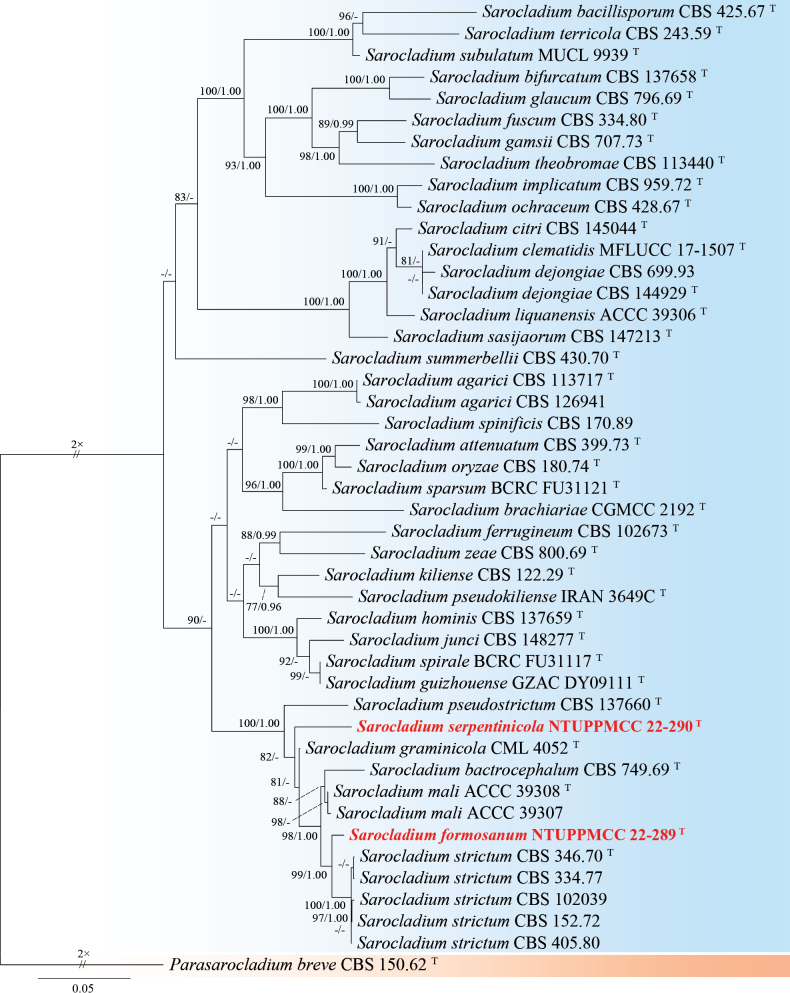
Maximum likelihood (ML) phylogenetic tree based on a concatenated dataset of ITS, LSU, *rpb2*, and *tef-1*. In total, 45 strains representing 38 taxa were included in the concatenated dataset, with 2821 characters (ITS 497 bp, LSU 779 bp, *rpb2* 734 bp, and *tef-1* 811 bp) including alignment gaps. The tree was rooted with *Parasarocladiumbreve* CBS 150.62. MLB ≥ 70% and BPPs ≥ 0.95 were shown at each node; values lower than these thresholds are indicated by a hyphen (–). The scale bar indicates the number of estimated substitutions per site. The strains introduced in this study are in red and novel species are in bold. The ex-type strains are marked with ^T^.

In the present study, *Sarocladiumformosanum* forms a distinct clade with strong support (99%/1.00) based on multi-locus phylogenetic analysis (Fig. 27). Moreover, *S.formosanum* shows significant genetic divergence from its closest relatives, ex-type strain of *S.strictum* (CBS 346.70) and *S.bactrocephalum* (CBS 749.69) with 95.5% identity to S.strictum (677/710 bp) and 93.1% identity to *S.bactrocephalum* (661/710 bp) in the *rpb2* gene. Morphologically, *S.formosanum* lacks adelophialides, schizophialides, and chlamydospores, which distinguishes it from *S.mali* (Fig. 26; Hou et al. 2019). Several *Sarocladium* species have been recorded in Taiwan. *S.spinificis* was reported as an endophyte of the coastal grass *Spinifexlittoreus* (Yeh and Kirschner 2014), while *S.attenuatum*, *S.oryzae*, *S.sparsum*, and *S.spirale* were isolated from rice grains and leaf sheaths (Ou et al. 2020). However, our species, *S.formosanum* and *S.serpentinicola*, were isolated from serpentine environments and formed distinct clades from these previously reported species in the phylogeny. While this study establishes *S.formosanum* as a distinct species, future studies should aim to recover additional isolates from similar environments to further validate its phenotypic variation and ecological distribution.

##### ﻿ Sarocladium serpentinicola

Taxon classificationAnimaliaHypocrealesSarocladiaceae

K.W. Cheng & H.A. Ariyaw. sp. nov.

3E11D41B-25ED-505D-96BA-609E70B9D02F

MB858716

Fig. 28

###### Typification.

TAIWAN • Guanshan Township, Taitung County, 23°02'14.8"N, 121°11'22.6"E, serpentine soil in rice field, 3^rd^ November 2022, K.W. Cheng, holotype, NTUPPMH 22-223 (Permanently preserved in a metabolically inactive state), ex-holotype NTUPPMCC 22-290.

###### Etymology.

Named after the serpentine soil from which the species was isolated.

###### Description.

***Sexual morph*** undetermined. ***Asexual morph*** Conidia were observed on WA. ***Conidiophores*** solitary, hyaline, straight to slightly flexuous, smooth-walled, arising from hyphal ropes or vegetative hyphae. ***Phialides*** subulate, hyaline, wide at the base, with, 23–32 µm long. ***Schizophialides*** not observed. ***Conidia*** unicellular, cylindrical with rounded ends, hyaline, smooth-walled, 1-celled, few with inconspicuous 1 to 2 guttules on the end(s), sometimes aggregated in clusters forming a slimy head, 3.2–5.5 µm × 1.2–1.8 µm (x̄ = 4.3 × 1.5 µm, L/W ratio = 2.92, n = 30). ***Adelophialides*** observed, 3.6–8.8 µm long and sporulated obvious guttules and larger conidia, 3.8–7.0 µm × 1.6–2.5 µm (x̄ = 5.3 × 2.0 µm, L/W ratio = 2.73, n = 30).

###### Culture characteristics.

Colony exhibit slow growth, reaching 30 mm diam with flat, pale orange, slightly wrinkled in the center, slimy, and smooth margin. The reverse side of the colony displayed similar characteristics.

###### Notes.

**Figure 28.**
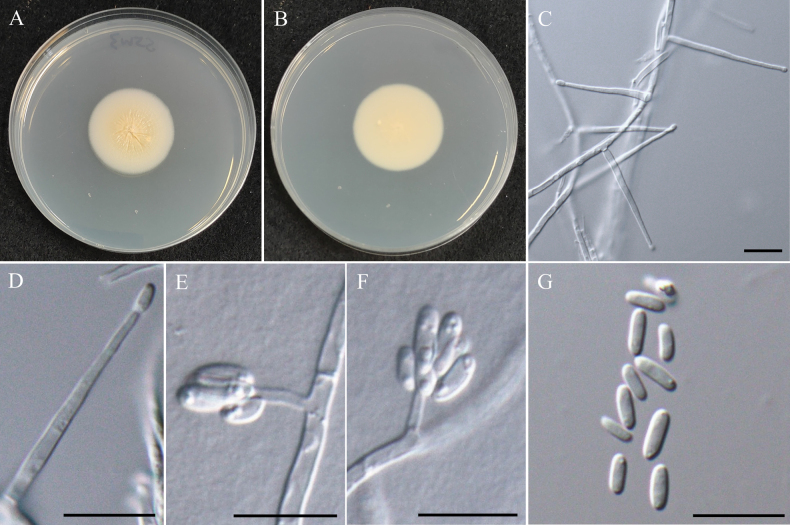
Morphology of *Sarocladiumserpentinicola*NTUPPMCC 22-290. **A, B** 14-days-old colony on PDA; **C, D** Conidiophores and phialides; **E, F** Adelophialides; **G** Conidia. Scale bars: 10 µm (**C–G**).

*Sarocladiumserpentinicola* introduced in this study forms a distinct clade with moderately support (82%/0.91) based on multi-locus phylogenetic analysis (Fig. 27). Moreover, *S.serpentinicola* forms significant genetic divergence to its closer relatives, ex-type strain of *S.pseudostrictum* (CBS 137660) with 96.0% identity in the ITS region (460/474 bp, including 5 gaps) and 96.2% identity in the *tef-1* gene (777/808 bp). It also exhibits notable divergence from *S.formosanum*, another novel species described in this study, with 91.3% identity in the *rpb2* gene (648/710 bp) and 96.9% identity in the *tef-1* gene (783/808 bp). *S.serpentinicola*NTUPPMCC 22-290 can be distinguished from its close relatives *S.pseudostrictum* and *S.graminicola* by the presence of adelophialides and the production of larger conidia from these structures (Fig. 28; Giraldo et al. 2015; Anjos et al. 2020).

#### ﻿*Stachybotryaceae* L. Lombard & P.W. Crous

##### ﻿ Dimorphiseta

Taxon classificationAnimaliaHypocrealesStachybotryaceae

L. Lombard & P.W. Crous

AF60A24D-3204-58FB-8334-73C4D8B121D7

###### Notes.

The genus *Dimorphiseta* was introduced to place *D.terrestris*, a strain originally isolated from soil habit in USA by Lombard and Crous (2016), and three species are recognized in MycoBank (Accession date: March 10, 2025). Conidiomata of these species are superficial, oval to elongate or irregular in outline, sporodochial, stromatic, cupulate, scattered or gregarious, and are covered by an olivaceous green mucoid layer. Three distinct types of setae are present: type I – hyaline, thin-walled, flexuous to circinate, verrucose, tapering to an obtuse apice; type II – hyaline, thick-walled, septate, smooth, tapering to a sharp apice; type III – hyaline, thin-walled, straight, terminating in an obtuse apex. Conidiophores are irregular, macronematous and smooth-walled. Conidiogenous cells are hyaline, cylindrical, phialidic, smooth, with conspicuous collarettes while conidia are hyaline, cylindrical, fusiform, smooth, aseptate, funnel-shaped mucoid apical appendage (Lombard et al. 2016; Liang et al. 2019). The species of *Dimorphiseta* have been reported from China, Taiwan, and USA occurring on soil and also as saprobes in plant species belonging to *Poaceae* and *Cannabaceae* families (Lombard et al. 2016; Liang et al. 2019; Tennakoon et al. 2021).

##### ﻿ Dimorphiseta formosana

Taxon classificationAnimaliaHypocrealesStachybotryaceae

K.W. Cheng & H.A. Ariyaw. sp. nov.

2B3F825A-F4D0-5C84-B24F-041C6E4EA0CF

MB858708

Fig. 29

###### Typification.

TAIWAN • Wanrung Township, Hualien County, 23°42'40.3"N, 121°24'48.2"E, serpentine soil in rice field, 2^nd^ November 2022, K.W Cheng, holotype, NTUPPMH 22-224 (Permanently preserved in a metabolically inactive state), ex-holotype NTUPPMCC 22-291.

###### Etymology.

Named after Formosa, the former name of Taiwan, where the type specimen was collected.

###### Description.

***Sexual morph*** undetermined. ***Asexual morph*** No sporulation on PDA and MEA, conidiomata produced very few on carnation leaves and surface of WA. ***Conidiomata*** 260–460 µm diam, randomly scattered, superficial, sporodochial, stromatic, subglobose to irregular in outline, dark green to black, agglutinated slimy mass of conidia. ***Setae*** thick-walled, hyaline, smooth, septate, straight to slightly curved, tapering to sharp apices, 170–240 µm long, 4–6 µm wide at broadest part. ***Conidiophores*** unbranched, hyaline to green, smooth to lightly verrucose, arising from basal stroma. ***Conidiogenous cells*** phialidic, hyaline, cylindrical, smooth, with collarette at tip, 17.2–22.7 µm × 1.7–2.3 µm (x̄ = 20 × 1.9 µm, n = 25). ***Conidia*** aseptate, hyaline, fusiform, smooth, few with funnel-shaped apical appendage, 7.8–9.0 µm × 2.1–3 µm (x̄ = 8.4 × 2.6 µm, L/W ratio = 3.32, n = 50).

###### Culture characteristics.

Colony reaching 60 mm diam with white, fluffy, cotton-like mycelium in center that gradually thinned toward the edges with a slightly irregular margin. A slight yellowish-green pigment diffused in PDA and the reverse side of the medium appeared canary yellow.

###### Notes.

**Figure 29.**
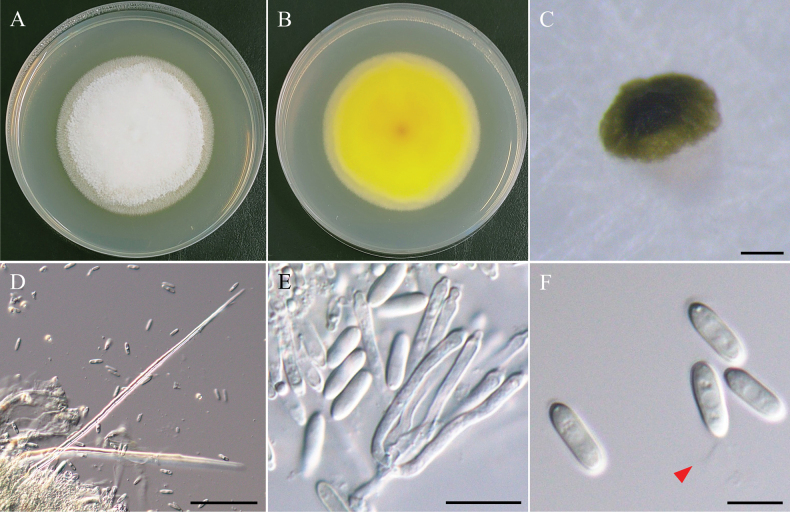
Morphology of *Dimorphisetaformosana*NTUPPMCC 22-291. **A, B** 14-days-old colony on PDA; **C** Conidiomata; **D** Setae; **E** Conidiophores and conidiogenous cells; **F** Conidia (red arrow: funnel-shaped appendage). Scale bars: 0.2 mm (**C**); 50 µm (**D**); 10 µm (**E, F**).

**Figure 30.**
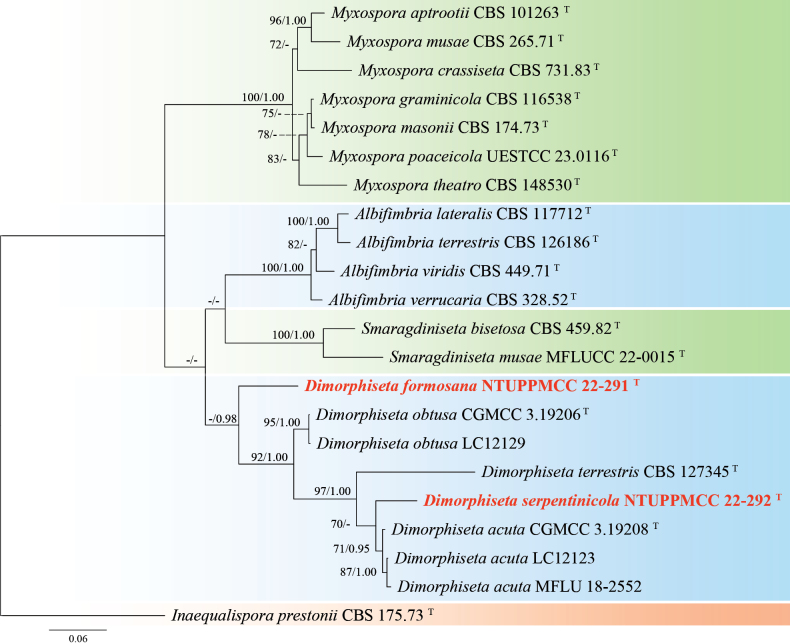
Maximum likelihood (ML) phylogenetic tree based on a concatenated dataset of ITS, *cmd*A, *rpb2*, and *tub2*. In total, 22 strains representing 19 taxa were included in the concatenated dataset, with 2424 characters (ITS 569 bp, *cmd*A 709 bp, *rpb2* 721 bp, and *tub2* 425 bp) including alignment gaps. The tree was rooted with *Inaequalisporaprestonii* CBS 175.73. MLB ≥ 70% and BPPs ≥ 0.95 were shown at each node; values lower than these thresholds are indicated by a hyphen (–). The scale bar indicates the number of estimated substitutions per site. The strains introduced in this study are in red and novel species are in bold. The ex-type strains are marked with ^T^.

This study introduces *Dimorphisetaformosana* as a new species, described from a single strain obtained from serpentine soil. *D.formosana* forms a distinct clade with moderately high statistical support (68%/0.98) based on multi-locus phylogenetic analysis (Fig. 30). Furthermore, *D.formosana* exhibits significant genetic divergence from its closest relative, the ex-type strain of *D.obtusa* (CGMCC 3.19206), across four loci: ITS (491/516 bp, identities 95.2%, including 1 gap), *cmd*A (501/572 bp, identities 87.6%, including 12 gaps), *rpb2* (608/721 bp, identities 84.3%), and *tub2* (254/307 identities, 82.7%, including 1 gap). Furthermore, our new species can be differentiated from *D.obtusa* (CGMCC 3.19206) by its smaller conidia, measuring 8–9 µm × 2–3 µm compared to 9–11 µm × 2–4 µm as reported by Liang et al. (2019) (Fig. 29). Even though its conidiogenous cells and conidia closely resemble those of the ex-type strain of *D.terrestris* (CBS 127345), our species lacks any Type I setae (Lombard et al. 2016). Additionally, while both *D.formosana* and *D.serpentinicola* were isolated from serpentine environments, *D.formosana* demonstrated a faster growth rate on PDA and produced a more vibrant yellow on the reverse side of the culture.

##### ﻿ Dimorphiseta serpentinicola

Taxon classificationAnimaliaHypocrealesStachybotryaceae

K.W. Cheng & H.A. Ariyaw. sp. nov.

BB4BCCB1-B2C2-5C1B-92CB-224F574DA617

MB858710

Fig. 31

###### Typification.

TAIWAN • Wanrung Township, Hualien County, 23°42'40.3"N, 121°24'48.2"E, serpentine soil in rice field, 2^nd^ November 2022, K.W Cheng, holotype, NTUPPMH 22-225 (Permanently preserved in a metabolically inactive state), ex-holotype NTUPPMCC 22-292.

###### Etymology.

Named for the serpentine soil from which the species was isolated.

###### Description.

***Sexual morph*** undetermined. ***Asexual morph*** No sporulation on PDA and MEA, conidiomata produced on carnation leaves. ***Conidiomata*** 258–548 µm diam, 70–225 µm deep, randomly scattered, superficial, sporodochial, stromatic, globose to subglobose, smooth outline, dark green to black, agglutinated slimy, mucoid mass of conidia. ***Setae*** thick-walled, hyaline, smooth, septate, straight to slightly curved, tapering to sharp apices, 180–280 µm long, 5–7 µm wide at broadest part. ***Conidiophores*** unbranched, hyaline to green, smooth to lightly verrucose, arising from basal stroma. ***Conidiogenous cells*** phialidic, hyaline, cylindrical, smooth, with collarette at tip, 18.3–23.5 µm × 1.6–2.2 µm (x̄ = 21.0 × 1.9 µm, n = 25). ***Conidia*** aseptate, hyaline, fusiform, smooth, funnel-shaped apical appendage, 7.4–8.7 µm × 2.2–3.1 µm (x̄ = 8.1 × 2.7 µm, L/W ratio = 3.07, n = 50).

###### Culture characteristics.

Colony reaching 38 mm diam with abundant white, cotton-like mycelium and slightly irregular margin. A mustard pigment developed, and the reverse side of the medium appeared pale yellow.

###### Notes.

**Figure 31.**
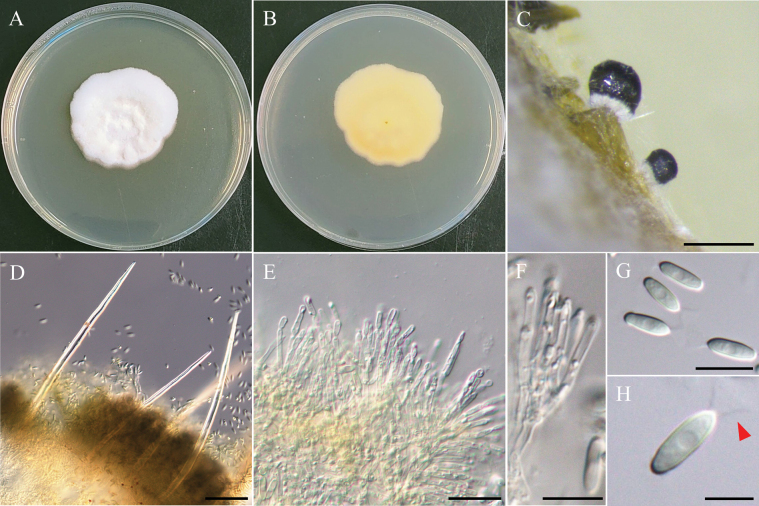
Morphology of *Dimorphisetaserpentinicola*NTUPPMCC 22-292. **A, B** 14-days-old colony on PDA; **C** Conidiomata; **D** Setae; **E, F** Conidiophores and conidiogenous cells; **G, H** Conidia (red arrow: funnel-shaped appendage). Scale bars: 0.5 mm (**C**); 50 µm (**D**); 20 µm (**E**); 10 µm (**F, G**); 5 µm (**H**).

The new taxon *D.serpentinicola* proposed in the present study forms a distinct clade with strong statistical support (97/1.00) based on multi-locus phylogenetic analysis (Fig. 30). Moreover, *D.serpentinicola* exhibits significant genetic divergence from its closest relative, the ex-type strain of *D.acuta* (CGMCC 3.19208), with 90.7% identity in the *cmd*A gene (526/580 bp, including 11 gaps) and 93.8% identity in the *rpb2* gene (676/721 bp). The colony color and texture on PDA were similar to *D.acuta* CGMCC 3.19208, but pigment diffusion into the medium was observed in PDA (Fig. 31; Liang et al. 2019). *D.acuta* was previous recorded in Taiwan, isolated from the dead leaves of *Celtisformosana* by (Tennakoon et al. 2021). However, our species exhibited smaller conidia (8.1 × 2.7 µm versus 10.5 × 2.5 µm) and longer setae (up to 280 µm versus 150 µm) compared to *D.acuta* (Tennakoon et al. 2021).

#### ﻿*Sordariales* Chadefaud ex D. Hawksworth & O.E. Eriksson

***Chaetomiaceae* G. Winter**

##### ﻿ Pseudothielavia

Taxon classificationAnimaliaSordarialesChaetomiaceae

X.W. Wang & Houbraken

01CF4D5B-2CC5-5FA2-AC2A-1CDB71E24306

###### Notes.

The genus *Pseudothielavia* was initially proposed by Wang and Houbraken (2019), to place *Coniothyriumterricola*, which is initially isolated from soil habitat. In recent years several species were introduced in this genus and currently four species epithets are listed in MycoBank (Accession date: March 10, 2025) for *Pseudothielavia*. Ascomata of these species are solitary to aggregated, globose or subglobose, superficial or submerged. They are typically non-ostiolate, though some species develop an ostiole at maturity. Peridium is brown, composed of textura epidermoidea, and may appear semi-translucent or translucent. Asci are clavate to pyriform, eight-spored and evanescent. Ascospores are 1-celled, olivaceous brown at maturity, smooth-walled, fusiform in shape and possess an apical, oblique or lateral germ pore (Wang et al. 2019). Species reported for *Pseudothielavia* are widely distributed and have been reported from Chile, China, Egypt, Japan, Papua New Guinea, and USA mainly from soil habitats (Wang et al. 2019; Zhang et al. 2021; Noguchi et al. 2022; Hussien et al. 2023).

##### ﻿ Pseudothielavia terricola

Taxon classificationAnimaliaTrichosphaerialesChaetomiaceae

X.W. Wang & Houbraken (2019)

0D8F09CD-4A1D-5EE3-B594-5D0B17AD81DB

MB829876

Fig. 32

###### Description.

***Sexual morph Cleistothecia*** 105–145 µm diam, non-ostiolate, globose, glabrous, black when mature, solitary to aggregated, mostly superficial, some submerged in PDA, aerial mycelium covered. ***Peridium*** brown, semi-translucent, membranous, textura epidermoidea. ***Asci*** subglobose to pyriform, hyaline when immature, eight-spored, 23.7–27.0 µm × 20.5–23.5 µm (x̄ = 25.5 × 22.5 µm, L/W ratio = 1.13, n = 10). ***Ascospores*** 1 celled, olivaceous brown when mature, subglobose to ellipsoidal, some smooth, apical germ pore, 9.3–11.7 µm × 6.7–9.0 µm (x̄ = 10.5 × 7.6 µm, L/W ratio = 1.39, n = 20). ***Asexual morph*** undetermined.

###### Culture characteristics.

Colony reaching 50 mm diam with thick white aerial mycelium, fluffy, edge irregularly, wavy margin, and similar to reverse side of the colony.

###### Material examined.

TAIWAN • Guanshan Township, Taitung County, 23°02'12.8"N, 121°11'22.0"E, serpentine soil in rice field, 2^nd^ November 2022, K.W Cheng, living culture NTUPPMCC 22-293 and NTUPPMCC 22-294.

###### Notes.

**Figure 32.**
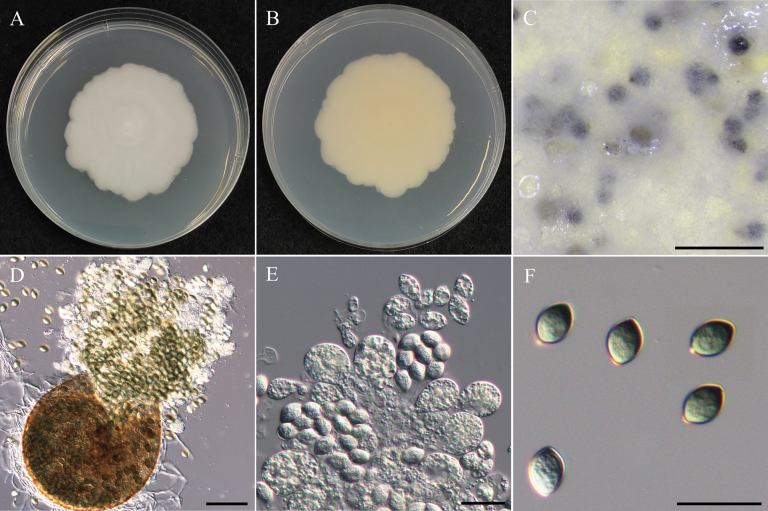
Morphology of *Pseudothielaviaterricola*NTUPPMCC 22-294. **A**, **B** 14-days-old colony on PDA; **C** Ascomata; **D** Squashed ascomata; **E** Immature ascus; **F** Ascospores. Scale bars: 1 mm (**C**); 50 µm (**D**); 20 µm (**E, F**).

**Figure 33.**
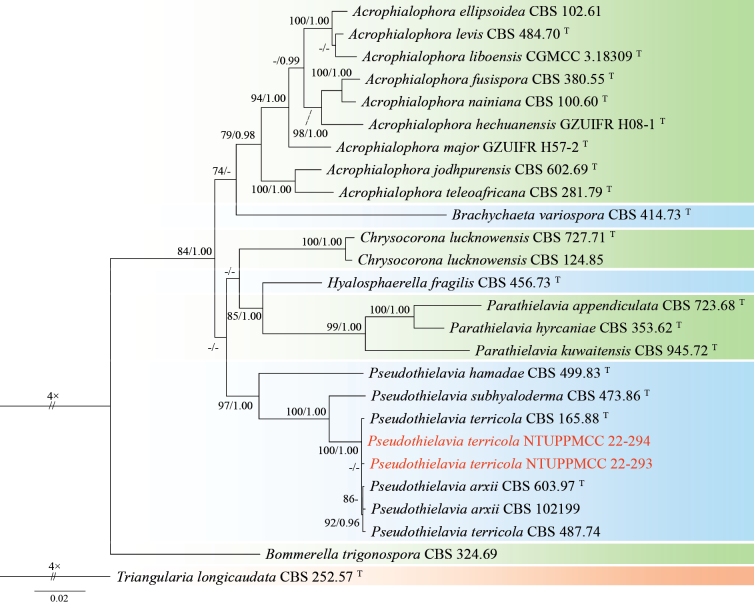
Maximum likelihood (ML) phylogenetic tree based on a concatenated dataset of ITS, LSU, *rpb2*, and *tub2*. In total, 26 strains representing 21 taxa were included in the concatenated dataset, with 2315 characters (ITS 531 bp, LSU 556 bp, *rpb2* 809 bp, and *tub2* 419 bp) including alignment gaps. The tree was rooted with *Triangularialongicaudata* CBS 252.57. MLB ≥ 70% and BPPs ≥ 0.95 were shown at each node; values lower than these thresholds are indicated by a hyphen (–). The scale bar indicates the number of estimated substitutions per site. The strains introduced in this study are in red and novel species are in bold. The ex-type strains are marked with ^T^.

Two strains (NTUPPMCC 22-293 and NTUPPMCC 22-294) isolated in this study clustered within the clade containing ex-type strains, along with other representative strains of *Pseudothielaviaarxii* and *Pse.terricola*, with strong statistical support (100%/1.00) (Fig. 33). Although both *Pse.arxii* and *Pse.terricola* exhibited no clear phylogenetic variation in our tree or in previous studies, they can be distinguished by differences in ascospore morphology (Wang et al. 2019). The morphology of our strain (NTUPPMCC 22-294) exhibited an apical germ pore, similar to that of *Pse.terricola* (CBS 165.88) (Fig. 32). According to its original description (Wang et al. 2019; Noguchi et al. 2022), *Pse.arxii* lacks an apical germ pore and instead possesses an oblique to lateral germ pore. Therefore, based solely on morphological similarity, we identified our strain as *Pse.terricola*. However, further studies are required to determine whether *Pse.arxii* and *Pse.terricola* represent two distinct species or a single species. This study represents the first discovery of a *Pseudothielavia* species in Taiwan.

#### ﻿*Naviculisporaceae* Y. Marín & Stchigel

##### ﻿ Pseudorhypophila

Taxon classificationAnimaliaSordarialesNaviculisporaceae

Y. Marín & Stchigel

069BB2A8-25C3-5931-B48B-276E83451003

###### Notes.

The genus *Pseudorhypophila* was proposed accommodate four *Zopfiella* species namely, *Zopfiellamangenotii*, *Z.marina*, *Z.pilifera*, and *Z.submersa*, which form a well-supported monophyletic clade within the family *Navicularisporaceae* in Harms et al. (2021). At present, four species are recognized in MycoBank (Accession date: March 10, 2025). Species in this genus are characterized in their sexual stage by immersed to erumpent ascomata that are non-ostiolate or ostiolate, and globose to subglobose or ovate to pyriform in shape. Asci are clavate to cylindrical, stipitate, and contain 4–8 spores, with a small apical ring that may sometimes be indistinct. Ascospores are biseriate and two-celled, often enclosed in gelatinous sheaths. They are hyaline and thin-walled. The upper cell is olivaceous brown to dark brown, usually narrowly conical with an acuminate apex and a rounded base; occasionally ovoid to limoniform, bearing an apical or subapical germ pore, and sometimes a distinct apical appendage. The lower cell remains hyaline, though it may occasionally appear pale olivaceous brown, pale brown, or even dark brown. It is straight and cylindrical, but may also be curved, hemispherical, or initially broadly obconical, later becoming flattened at the apex (Guarro et al. 1997; Harms et al. 2021). In the asexual stage *Pseudorhypophila* species produce holoblastic hyaline conidia that are spherical to subspherical, or ovate to elongate, smooth-walled, sessile, borne singly along the vegetative hyphae (Harms et al. 2021; Liu et al. 2025). *Pseudorhypophila* has been reported from Egypt, France, Iraq, Japan, South Korea and Taiwan, occurring on substrates such as freshwater, plant debris, soil, and mud (Guarro et al. 1997; Chang et al. 2010; Marin-Felix et al. 2020; Hussien et al. 2023; Liu et al. 2025).

##### ﻿ Pseudorhypophila formosana

Taxon classificationAnimaliaTrichosphaerialesNaviculisporaceae

K.W. Cheng & H.A. Ariyaw. sp. nov.

C97BC4B5-3894-56EA-AECA-84F0FDECD4B4

MB858713

Fig. 34

###### Typification.

TAIWAN • Guanshan Township, Taitung County, 23°02'14.8"N, 121°11'22.6"E, serpentine soil in rice field, 3^rd^ November 2022, K.W. Cheng, holotype, NTUPPMH 22-226 (Permanently preserved in a metabolically inactive state), ex-holotype NTUPPMCC 22-297, ex-isotype NTUPPMCC 22-295, 296, 298 to 300.

###### Etymology.

Named after Formosa, the former name of Taiwan, where the type specimen was collected.

###### Description.

***Sexual morph Ascomata*** 342–504 µm diam, non-ostiolate, globose, dirty gray when immature, black when mature, submerged in PDA. ***Peridium*** multi-layered, brown, translucent, membranous, angular cells. ***Asci*** clavate to cylindrical, hyaline when immature, eight-spored, 74–111 µm × 14–21 µm (x̄ = 90.9 × 17.8 µm, L/W ratio = 5.13, n = 20). ***Ascospores*** two-celled; ***upper cell*** ellipsoidal to slightly fusiform, smooth, brown, multiple guttules, subapical germ pore, 18.3–24.6 µm × 8.8–12.5 µm (x̄ = 21.3 × 11.0 µm, L/W ratio = 1.95, n = 50), lower cell cylindrical with slightly tapering or rounded end, hyaline to pale brown, thin-walled, 4.0–8.0 µm × 3.1–5.8 µm (x̄ = 5.8 × 4.3 µm, L/W ratio = 1.36, n = 50). ***Asexual morph*** undetermined.

###### Culture characteristics.

Colony exhibit rapid growth, reaching 80 mm diam with flat, sparse aerial mycelium, white, surface and margins slight smooth. The reverse exhibited blackish-gray center, with a gradient radiating outward into lighter gray tones.

###### Notes.

**Figure 34.**
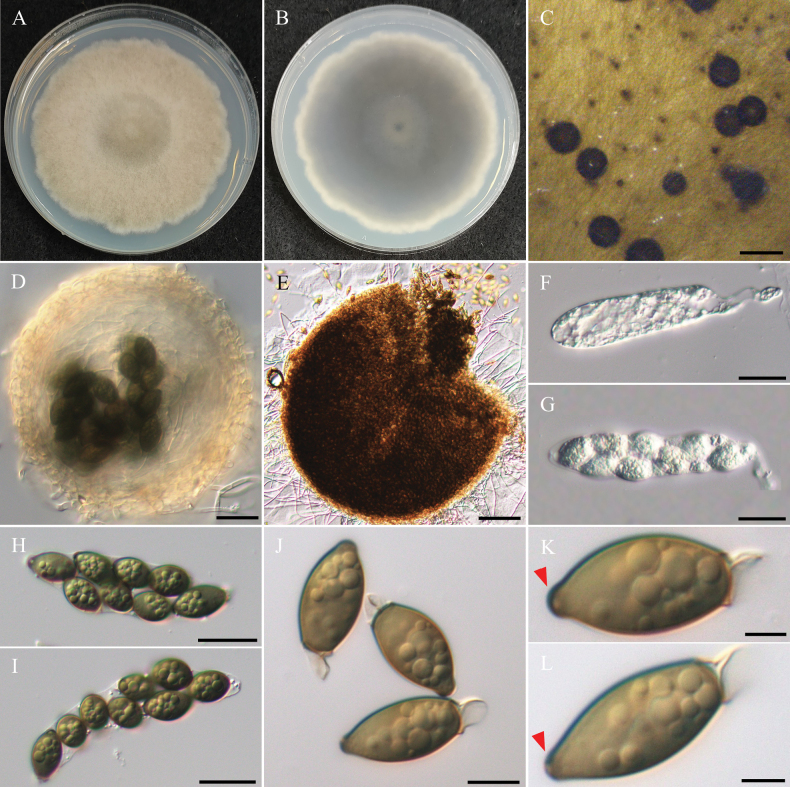
Morphology of *Pseudorhypophilaformosana*NTUPPMCC 22-297. **A, B** 14-days-old colony on PDA; **C** Ascomata; **D** Immature ascomata; **E** Squashed ascomata; **F, G** Immature ascus; **H, I** Ascus; **J** Ascospores; **K, L** Ascospores (red arrows: subapical germ pores). Scale bars: 0.5 mm (**C**); 100 µm (**E**); 20 µm (**D, F–I**); 10 µm (**J**); 5 µm (**K–L**).

**Figure 35.**
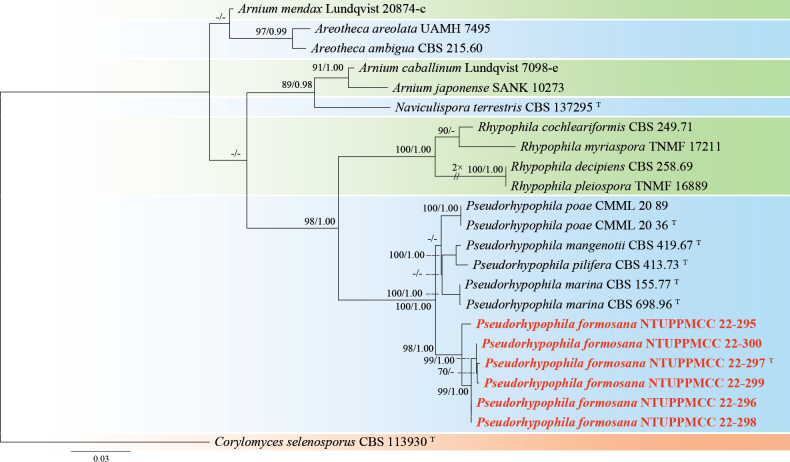
Maximum likelihood (ML) phylogenetic tree based on a concatenated dataset of ITS, LSU, and *rpb2*. In total, 23 strains representing 16 taxa were included in the concatenated dataset, with 2444 characters (ITS 569 bp, LSU 869 bp, and *rpb2* 1006 bp) including alignment gaps. The tree was rooted with *Corylomycesselenosporus* CBS 113930. MLB ≥ 70% and BPPs ≥ 0.95 were shown at each node; values lower than these thresholds are indicated by a hyphen (–). The scale bar indicates the number of estimated substitutions per site. The strains introduced in this study are in red and novel species are in bold. The ex-type strains are marked with ^T^.

In the present study, six *Pseudorhypophila* strains (NTUPPMCC 22-295 to 300) formed a distinct clade with a strong statistical support (100%/1.00), clearly separating from known *Pseudorhypophila* species in the multi-locus phylogenetic analysis (Fig. 35). Furthermore, the ex-type strain of *Pseudorhypophilaformosana* (NTUPPMCC 22-297) exhibits significant genetic divergence from its closest relatives, the ex-type strains of *P.mangenotii* (CBS 419.67) and *P.poae* (CMML 20-36), with 94.0% (936/996 bp) and 94.2% (938/996 bp) identity, respectively, in the *rpb2* gene. In line with earlier research on *P.marina* and *P.pilifera*, our isolate produces ascomata lacking ostioles and bearing 2-celled ascospores, a distinctive trait of the *Pseudorhypophila* (Harms et al. 2021). However, *P.formosana*NTUPPMCC 22-297 is distinguished by its lower cell lengths, which are noticeably smaller than those of the type species, *P.marina* CBS 698.96 (4–8 µm × 3–6 µm versus 6–13 µm × 3–5 µm) (Fig. 34; Guarro et al. 1997). Moreover, *P.poae* has been recorded only in its asexual morph, which was not observed in our strains (NTUPPMCC 22-295 to 300). Notably, the other representative strain of *P.marina* (*Zopfiellamarina* CBS 155.77) was previously reported from Taiwan. However, it also differs from *P.formosana*NTUPPMCC 22-297 in morphology, phylogeny and habitat (marine mud versus terrestrial serpentine soil) (Overy et al. 2014; Harms et al. 2021).

#### ﻿*Trichosphaeriales* M.E. Barr

***Trichosphaeriaceae* G. Winter**

##### ﻿ Phialoparvum

Taxon classificationAnimaliaTrichosphaerialesTrichosphaeriaceae

Giraldo López & Crous

99AFEC00-6021-5D96-99B0-EB42DC3C508D

###### Notes.

The genus *Phialoparvum* was introduced by Giraldo and Crous (2019) to place *Phialoparvumbifurcatum*, which was isolated from soil habitat in Belgium. To date, three species are recognized in MycoBank (Accession date: March 10, 2025) for *Phialoparvum*. The genus was introduced solely based on the asexual morph and characterized by its erect, originating directly from vegetative hyphae or hyphal ropes, which can be either unbranched or poorly branched conidiophores (Giraldo and Crous 2019). Conidiogenous cell of this genus is enteroblastic, hyaline, mono-, poly-, and adelophialides, subulate to ampulliform while forming conspicuous collarette. Conidia are cylindrical, hyaline, unicellular, smooth-walled, and aggregated in slimy heads (Giraldo et al. 2019).

##### ﻿ Phialoparvum formosanum

Taxon classificationAnimaliaTrichosphaerialesTrichosphaeriaceae

K.W. Cheng & H.A. Ariyaw. sp. nov.

0C2FB4C5-0827-5A60-9BF0-1601A0C4538C

MB858712

Fig. 36

###### Typification.

TAIWAN • Guanshan Township, Taitung County, 23°02'14.8"N, 121°11'22.6"E, serpentine soil in rice field, 3^rd^ November 2022, K.W. Cheng, holotype, NTUPPMH 227 (Permanently preserved in a metabolically inactive state), ex-holotype NTUPPMCC 22-301.

###### Etymology.

Named after Formosa, the former name of Taiwan, where the type specimen was collected.

###### Description.

***Sexual morph*** undetermined. ***Asexual morph*** Conidia were observed on WA. ***Conidiophores*** solitary, hyaline, straight to slightly flexuous, arising from hyphal ropes or vegetative hyphae. ***Phialides*** subulate to ampulliform, hyaline, terminal or lateral, with conspicuous periclinal thickening and cylindrical collarette, 4–18 µm long. Mono-phialides or adelophialides predominant, few poly-phialides with two conidiogenous loci. ***Conidia*** cylindrical, hyaline, smooth, thick-walled, 1-celled, with 1 to 2 guttules, sometimes aggregated in clusters forming a slimy head, 3.6–4.6 µm × 1.7–2.4 µm (x̄ = 4.2 × 2.1 µm, L/W ratio = 2.04, n = 50).

###### Culture characteristics.

Colony exhibit slow growth, reaching 30 mm diam with flat, creamy white, and velvety to powdery at the center, gradually thinning toward the edges with a smooth margin. The reverse side of the colony displayed similar characteristics.

###### Notes.

**Figure 36.**
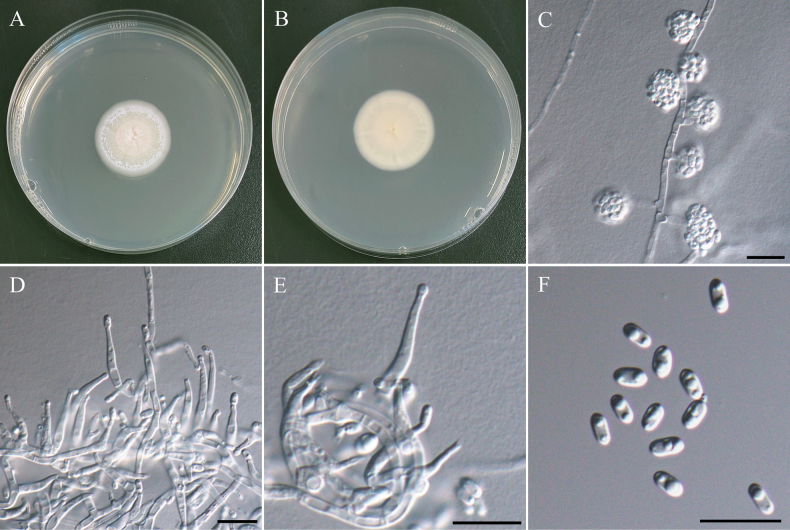
Morphology of *Phialoparvumformosanum*NTUPPMCC 22-301. **A, B** 14-days-old colony on PDA; **C–E** Conidiophores, phialides, and conidiogenous cells; **F** Conidia. Scale bars: 10 µm (**C–F**).

**Figure 37.**
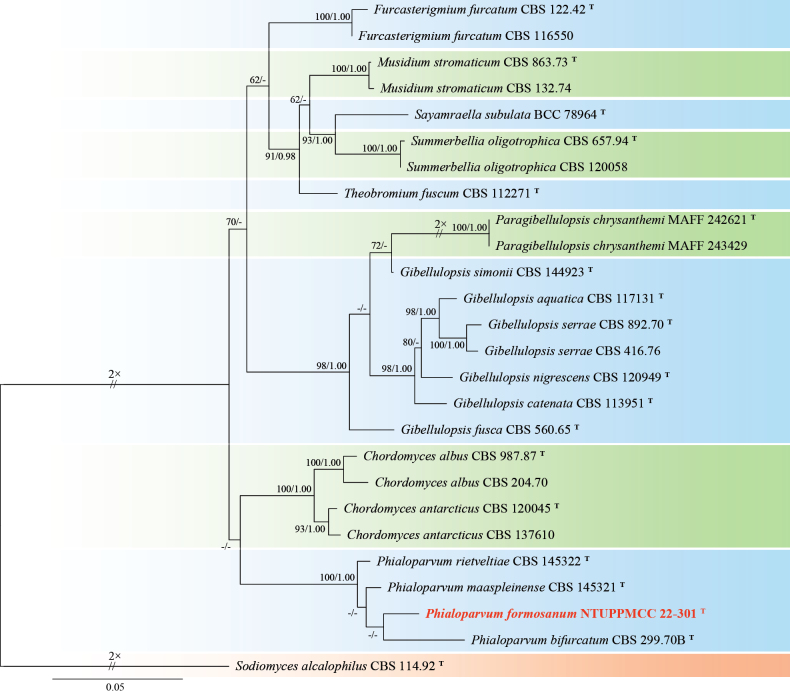
Maximum likelihood (ML) phylogenetic tree based on a concatenated dataset of ITS, LSU, *rpb2*, and *tef-1*. In total, 26 strains representing 19 taxa were included in the concatenated dataset, with 2810 characters (ITS 477 bp, LSU 798 bp, *rpb2* 748 bp, and *tef-1* 787 bp) including alignment gaps. The tree was rooted with *Sodiomycesalcalophilus* CBS 114.92. MLB ≥ 70% and BPPs ≥ 0.95 were shown at each node; values lower than these thresholds are indicated by a hyphen (–). The scale bar indicates the number of estimated substitutions per site. The strains introduced in this study are in red and novel species are in bold. The ex-type strains are marked with ^T^.

In our multi-locus phylogenetic assessment, *Phialoparvumformosanum* forms a distinct branch with low statistical support, sister to the clade containing the ex-type strain of *Ph.bifurcatum* (CBS 299.70B). Despite the weak nodal support, these taxa exhibit substantial genetic divergence across three loci: ITS (440/455 bp, identities 96.7%, including 7 gaps), *rpb2* (259/279 bp, identities 92.8%), and *tef-1* (761/787 bp, identities 96.7%) (Fig. 37 and Suppl. material 2: figs S3–S5). *Ph.formosanum*NTUPPMCC 22-301 shares typical characteristics of the genus *Phialoparvum*, including hyaline, solitary, arising from hyphal ropes or vegetative hyphae of conidiophores and subulate to ampulliform phialides. However, the conidia of *Ph.formosanum*NTUPPMCC 22-301 are broader than the type strain of *Ph.bifurcatum* CBS 299.70B (3.6–4.6 µm × 1.7–2.4 µm versus 2.8–4.4 µm × 1.2–1.8 µm) (Fig. 36; Giraldo and Crous 2019). Additionally, *Ph.formosanum* exhibits prominent guttules in its conidia, a feature that differentiates it from the other three *Phialoparvum* species (Fig. 36; Giraldo and Crous 2019; Giraldo et al. 2019).

## ﻿Discussion

The present study provides the first comprehensive analysis of culturable fungal flora in serpentine-characterized paddy soils of eastern Taiwan. The results of the present study reveal a unique mycobiota dominated by *Dothideomycetes* (60% of isolates), with *Westerdykella*, *Pyrenochaetopsis*, and *Talaromyces* as the most frequently isolated genera. This contrasts with previous studies of serpentine soils, where *Aspergillus*, *Cladosporium*, and *Penicillium* were frequently isolated as dominant taxa (Pal et al. 2007; Daghino et al. 2012). The absence of these genera in our study may indicate ecological specialization related to agricultural practices. For example, paddy soils are periodically flooded, creating anaerobic conditions that favor fungal groups such as *Westerdykella* and *Pyrenochaetopsis*, which are frequently reported from waterlogged or plant-associated environments (Goh et al. 2021; Surono et al. 2023). Moreover, the use of half-strength media to suppress fast-growing fungi may have selectively enriched slower-growing, stress-adapted species, further shaping community composition (Su et al. 2012). Especially, 12 genera including *Dimorphiseta*, *Parasarocladium*, *Phialoparvum*, *Poaceascoma*, *Pseudorhypophila*, *Pseudothielavia*, *Pseudoxylomyces*, *Pyrenochaetopsis*, *Reticulascus*, *Sarocladium*, *Setophaeosphaeria*, and *Westerdykella* as a new record in serpentine environment are reported for the first time in serpentine environments, highlighting the ecological uniqueness of these metal-rich soils. It is noteworthy that *Poaceascomaserpentinum* represents the first documented asexual morph within its genus, suggesting adaptive strategies such as reduced sexual reproduction in response to heavy metal stress (Ali 2007; Gajewska et al. 2022; Zhang et al. 2024). The discovery of 11 novel species and 11 new records for Taiwan highlights serpentine ecosystems as reservoirs of fungal diversity and endemism, consistent with the high species abundance observed in other extreme environments (Oze et al. 2008).

Notably, in our study, 35% of the strains belong to *Westerdykella*. It is worth conducting further research to understand the role this genus plays in serpentine paddy fields. As mentioned earlier in this manuscript, we encountered several challenges in determining the species boundaries of *Westerdykelladispersa*. One possible reason for this is that only the ex-type strain (CBS 297.56) of *W.dispersa*, along with the strains identified as *W.dispersa* in this study, have complete sequence data for ITS, LSU, and *tub2*. In contrast, all other *W.dispersa* strains available in public databases are represented only by ITS and LSU sequences. This lack of complete genetic data may hinder accurate species delimitation and raises uncertainty about whether *W.dispersa* strains in Clades A and C represent distinct species or belong to the same species population. Additionally, most previous reports of *W.dispersa* lack detailed morphological descriptions, further complicating taxonomic resolution. Therefore, to clarify the species status of the two clades, it is essential to obtain the missing β-tubulin (*tub2*) sequences and conduct detailed morphological comparisons between the reference strains and the strains analyzed in this study. In a previous study, *Westerdykellaaquatica* P71, a phylogenetically close relative of the strains isolated in this study, demonstrated mercury phytotoxicity mitigation in maize (Senabio et al. 2023). This suggests that the isolates identified in our study could serve as promising candidates for bioremediation, particularly in agricultural systems affected by serpentine-derived heavy metals. Future studies should quantify their metal uptake capacities and evaluate their symbiotic potential with crops to enhance phytostabilization.

The genus *Sarocladium* is commonly associated with rice pathogens (Pramunadipta et al. 2020). Several *Sarocladium* species, including *S.attenuatum*, *S.oryzae*, *S.sparsum*, and *S.spirale*, have been reported in Taiwan paddy fields prior to this study (Ou et al. 2020). In our study, we have identified two novel species of *Sarocladium*. However, because our strains were isolated from soil, further examination is necessary to determine whether these species have pathogenicity towards rice. Additionally, *Sarocladium* species have been isolated from coal mine soil and have exhibited high levels of resistance to cadmium (Zhang et al. 2024). Thus, further research is essential to investigate whether these strains have the ability to develop as a potential bio-remediation agent against cadmium.

It is worth noting that while introducing *Pseudorhypophilaformosana* in the present study, we observed that one of the strains, NTUPPMCC 22-295, formed a clade basal to the main *P.formosana* clade, which includes strains NTUPPMCC 22-296 to 22-300, with strong statistical support (98%/1.00) (see Fig. 35). However, upon examining the morphology of NTUPPMCC 22-295, we did not observe any significant differences in culture characteristics or micromorphological features compared to the other strains. Furthermore, sequence identities across three single-loci (ITS, LSU, and *rpb2*) all exceeded 97%, indicating no substantial genetic divergence. Therefore, we consider all six strains to be conspecific and assign them to *Pseudorhypophilaformosana*.

In the present study, we proposed several novel species based on single strains obtained from serpentine soil (*Dimorphisetaformosana*, *D.serpentinicola*, *Parasarocladiumformosum*, *Phialoparvumformosanum*, *Poaceascomaserpentinum*, *Sarocladiumformosanum*, and *S.serpentinicola*). For instance, phylogenetic analyses revealed significant genetic divergence in *Poaceascomaserpentinum*, which showed less than 88% ITS sequence identity with its closest relatives, supporting its classification as a distinct species. However, relying on single strains for some descriptions highlights a common limitation in studies of rare environmental fungi (Crous et al. 2018a; Hurdeal et al. 2022). Although each species is based on a single isolate, detailed morphological and robust phylogenetic evidence support their recognition as distinct taxa. Ideally, multiple strains should be used to understand intraspecific variation and ensure taxonomic stability (Aime et al. 2021). However, collecting additional isolates was challenging due to the fungi’s specialized ecological niches and the limited availability of suitable microhabitats in serpentine soils. While our findings provide strong evidence for the uniqueness of these species, future studies should aim to sample more extensively from similar habitats to investigate their morphological variability, ecological range, and potential cryptic diversity.

A key limitation of this study is its focus on culturable fungi, which likely underestimates the total fungal diversity. Metagenomic approaches, such as ITS amplicon sequencing, could uncover non-culturable taxa and provide comprehensive insights into community functional profiles (Koner et al. 2023). Additionally, linking fungal diversity to soil physicochemical parameters (e.g., Ni/Cr concentrations, pH) would help elucidate the relative impacts of metal toxicity and edaphic factors in shaping microbial community structure. In conclusion, our research expands the frontiers of fungal ecology by uncovering the remarkable adaptive capabilities of microorganisms in serpentine ecosystems. The predominance of asexual morphs and exceptional metal tolerance reveal the significant evolutionary strategies of fungal communities under extreme stress. By integrating fundamental research with multi-omics approaches and targeted field trials, we open promising avenues for understanding microbial adaptation with potential applications in phytoremediation, sustainable agriculture, and ecosystem management.

## Supplementary Material

XML Treatment for Poaceascoma

XML Treatment for Poaceascoma serpentinum

XML Treatment for Pseudoxylomyces

XML Treatment for Pseudoxylomyces aquaticus

XML Treatment for Pyrenochaetopsis

XML Treatment for Pyrenochaetopsis paucisetosa

XML Treatment for Pyrenochaetopsis oryzicola

XML Treatment for Setophaeosphaeria

XML Treatment for Setophaeosphaeria microspora

XML Treatment for Westerdykella

XML Treatment for Westerdykella aquatica

XML Treatment for Westerdykella capitulum

XML Treatment for Westerdykella dispersa

XML Treatment for Westerdykella formosana

XML Treatment for Westerdykella globosa

XML Treatment for Talaromyces

XML Treatment for Talaromyces adpressus

XML Treatment for Talaromyces taiwanensis

XML Treatment for Talaromyces thailandensis

XML Treatment for Cylindrotrichum

XML Treatment for Cylindrotrichum formosanum

XML Treatment for Parasarocladium

XML Treatment for Parasarocladium formosum

XML Treatment for Sarocladium

XML Treatment for Sarocladium formosanum

XML Treatment for Sarocladium serpentinicola

XML Treatment for Dimorphiseta

XML Treatment for Dimorphiseta formosana

XML Treatment for Dimorphiseta serpentinicola

XML Treatment for Pseudothielavia

XML Treatment for Pseudothielavia terricola

XML Treatment for Pseudorhypophila

XML Treatment for Pseudorhypophila formosana

XML Treatment for Phialoparvum

XML Treatment for Phialoparvum formosanum

## ﻿Additional information

### Adherence to national and international regulations

All the fungal strains used in this study have been legally obtained, respecting the Convention on Biological Diversity (Rio Convention).

### Author ORCIDs

Jiue-in Yang https://orcid.org/0000-0002-2254-4151

Piroonporn Srimongkol https://orcid.org/0000-0002-0319-1573

Marc Stadler https://orcid.org/0000-0002-7284-8671

Aphichart Karnchanatat https://orcid.org/0000-0002-6598-013X

Hiran A. Ariyawansa https://orcid.org/0000-0001-8526-7721

### Data availability

All of the data that support the findings of this study are available in the main text or Supplementary Information.

## Supplementary materials

Supplementary material 1 Supplementary tables S1–S4 Data type xlsx Explanation note **table S1.** List of primers used in this study; **table S2.** Genbank accession numbers for the strains in this study (type strains in bold and new strains in red); **table S3.** The nucleotide substitution models used in the phylogenetic analyses. **table S4.** List of *Westerdykella* species morphology. https://binary.pensoft.net/file/1362349This dataset is made available under the Open Database License (http://opendatacommons.org/licenses/odbl/1.0/). The Open Database License (ODbL) is a license agreement intended to allow users to freely share, modify, and use this Dataset while maintaining this same freedom for others, provided that the original source and author(s) are credited.Kai-Wen Cheng, Jiue-in Yang, Piroonporn Srimongkol, Marc Stadler, Aphichart Karnchanatat, Hiran A. Ariyawansa

Supplementary material 2 Supplementary figures S1–S5 Data type docx Explanation note **figure S1.** Maximum likelihood (ML) phylogenetic tree based on *tub2*; **figure S2.** Maximum likelihood (ML) phylogenetic tree based on ITS; **figure S3.** Maximum likelihood (ML) phylogenetic tree based on ITS; **figure S4.** Maximum likelihood (ML) phylogenetic tree based on *rpb2*; figure S5. Maximum likelihood (ML) phylogenetic tree based on *tef-1*. https://binary.pensoft.net/file/1362350This dataset is made available under the Open Database License (http://opendatacommons.org/licenses/odbl/1.0/). The Open Database License (ODbL) is a license agreement intended to allow users to freely share, modify, and use this Dataset while maintaining this same freedom for others, provided that the original source and author(s) are credited.Kai-Wen Cheng, Jiue-in Yang, Piroonporn Srimongkol, Marc Stadler, Aphichart Karnchanatat, Hiran A. Ariyawansa
