# Supplementary material for: ﻿Fungal frontiers in toxic terrain: Revealing culturable fungal communities in Serpentine paddy fields of Taiwan
Source: IMA Fungus. 2025 Jun 27;16:e155308. doi: 10.3897/imafungus.16.155308 (PMC12355036; doi:10.3897/imafungus.16.155308)
Supplement: Supplementary material 2 — Supplementary figures S1–S5 [file imafungus-16-e155308-s002.docx]

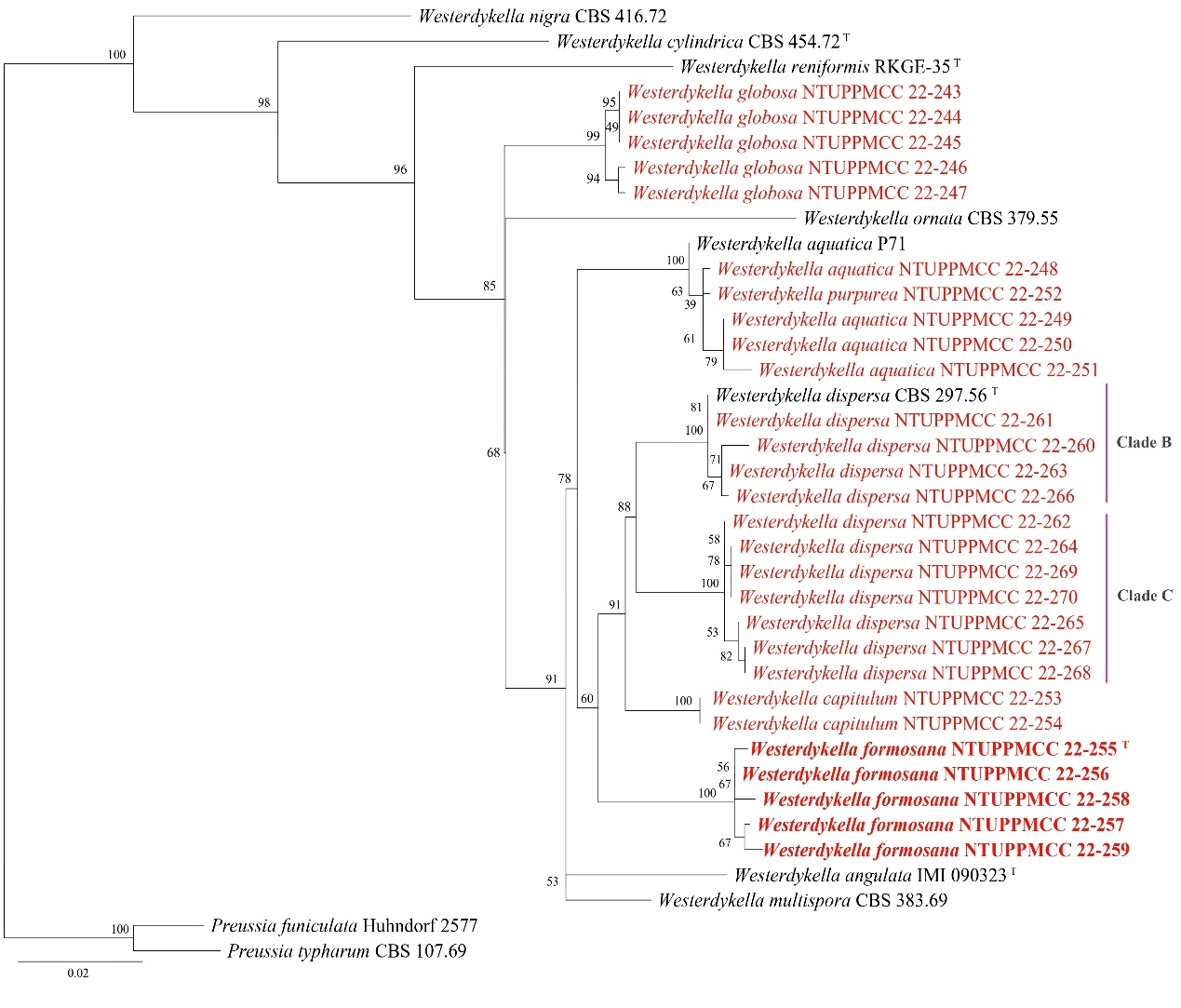


**Supplementary Figure S1.** Maximum likelihood (ML) phylogenetic tree based on *tub2*. The tree was rooted with *Preussia funiculata* Huhndorf 2577 and *P. typharum* CBS 107.69. The scale bar indicates the number of estimated substitutions per site. The strains introduced in this study are in red and novel species are in bold. The ex-type strains are marked with ^T^.


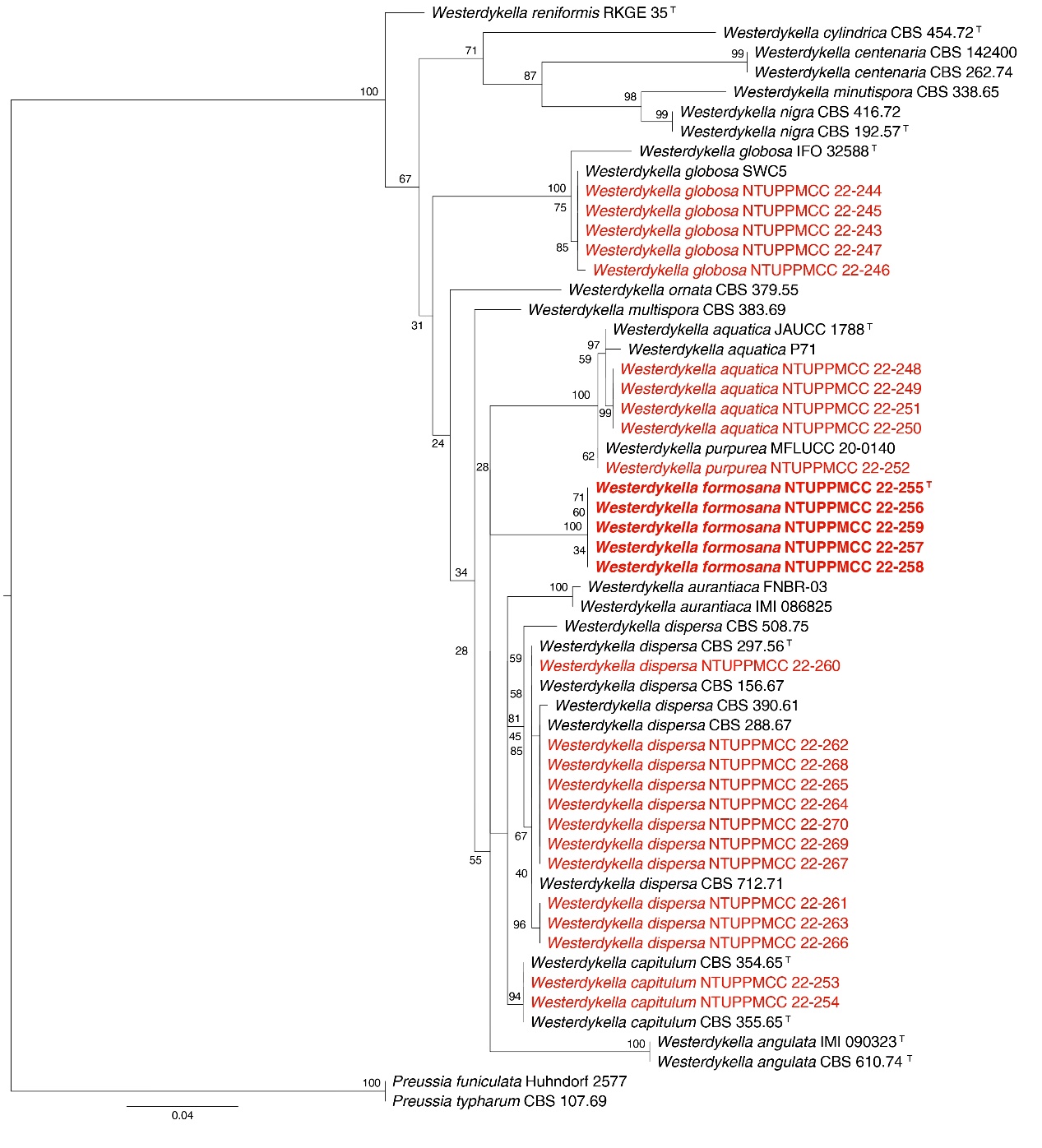


**Supplementary Figure S2.** Maximum likelihood (ML) phylogenetic tree based on ITS. The tree was rooted with *Preussia funiculata* Huhndorf 2577 and *P. typharum* CBS 107.69. The scale bar indicates the number of estimated substitutions per site. The strains introduced in this study are in red and novel species are in bold. The ex-type strains are marked with ^T^.


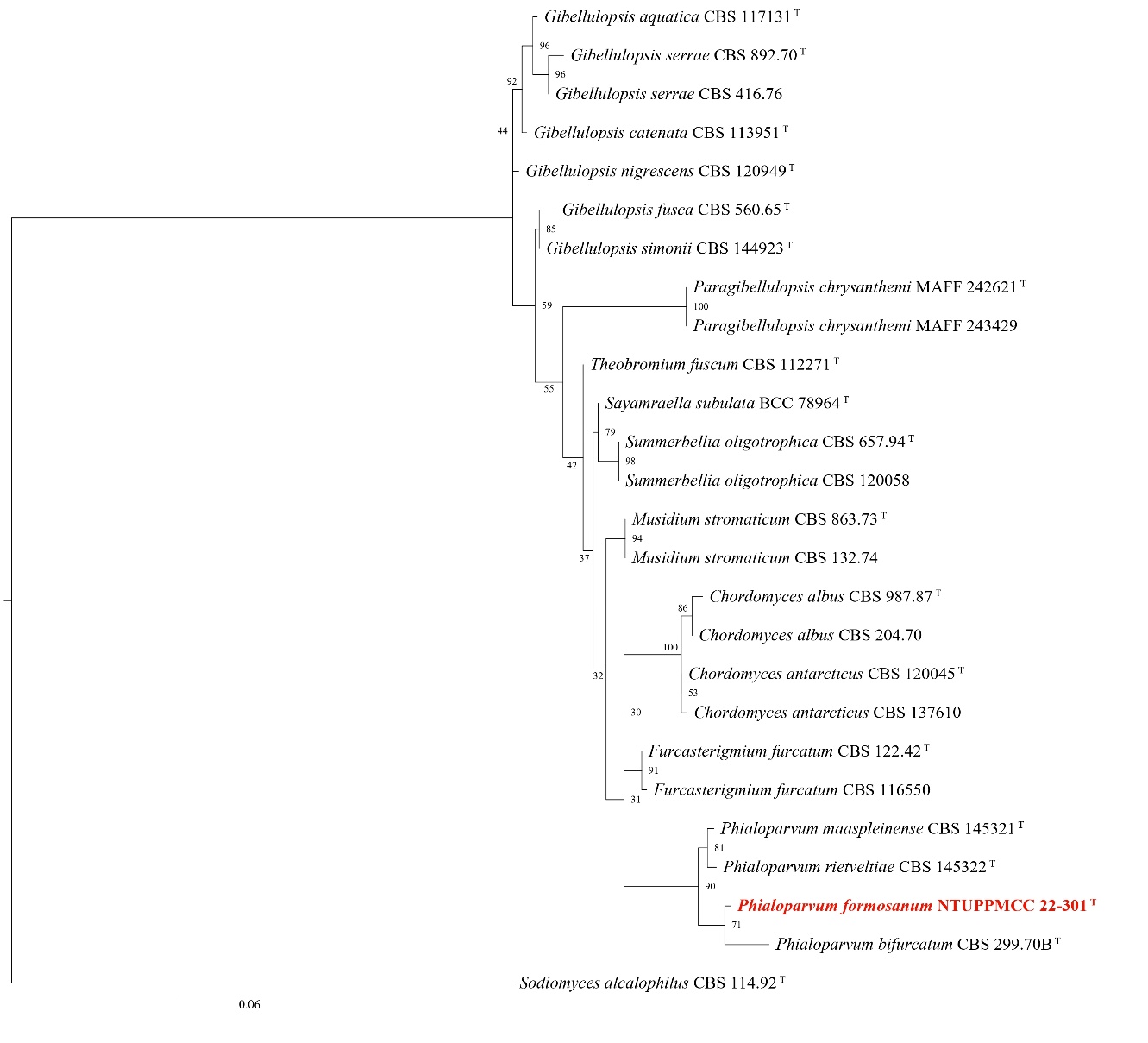


**Supplementary Figure S3.** Maximum likelihood (ML) phylogenetic tree based on ITS. The tree was rooted with *Sodiomyces alcalophilus* CBS 114.92. The scale bar indicates the number of estimated substitutions per site. The strains introduced in this study are in red and novel species are in bold. The ex-type strains are marked with ^T^.


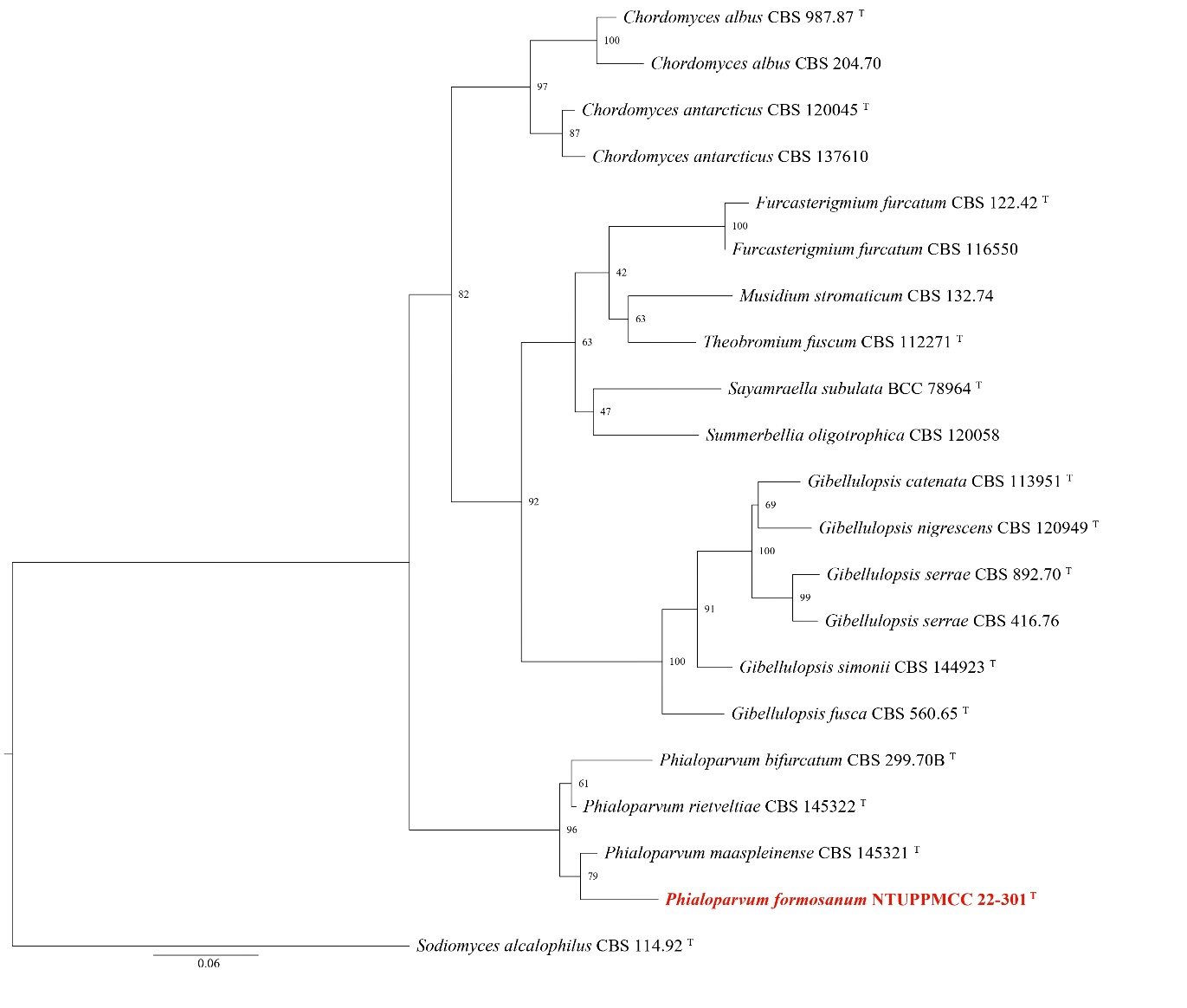


**Supplementary Figure S4.** Maximum likelihood (ML) phylogenetic tree based on *rpb2*. The tree was rooted with *Sodiomyces alcalophilus* CBS 114.92. The scale bar indicates the number of estimated substitutions per site. The strains introduced in this study are in red and novel species are in bold. The ex-type strains are marked with ^T^.


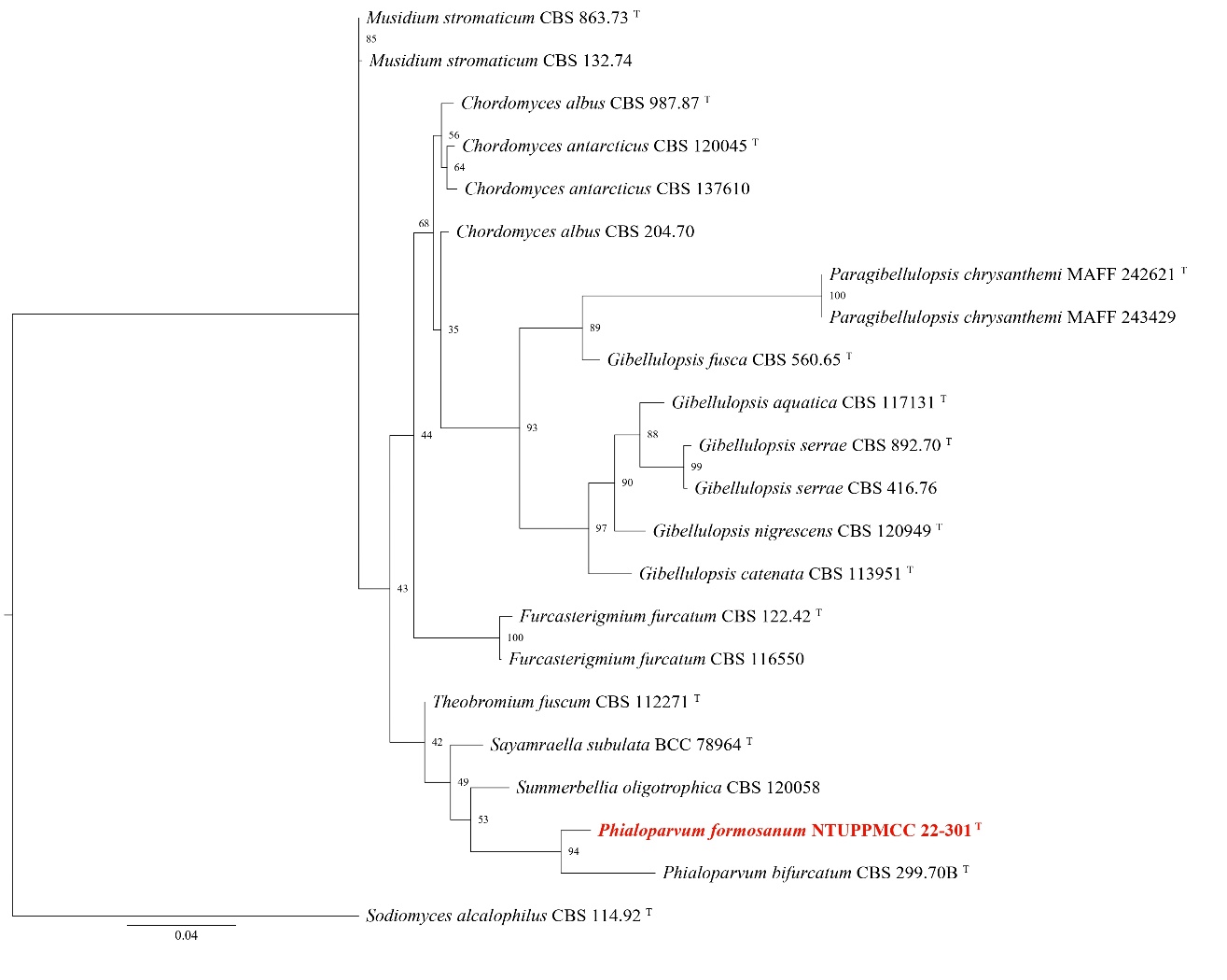


**Supplementary Figure S5.** Maximum likelihood (ML) phylogenetic tree based on *tef-1*. The tree was rooted with *Sodiomyces alcalophilus* CBS 114.92. The scale bar indicates the number of estimated substitutions per site. The strains introduced in this study are in red and novel species are in bold. The ex-type strains are marked with ^T^.
